# Tissue-specific regulation of a sugar transporter mediates both resistance and tolerance responses to attack from a leafminer in potato

**DOI:** 10.1126/sciadv.adx5951

**Published:** 2025-10-29

**Authors:** Zhiyao Mao, Asim Munawar, Xuan Chen, Jian Zhong, Shuzhen Wang, Yali Sang, Han Guo, Zengrong Zhu, Pengjun Zhang, Ian T. Baldwin, Wenwu Zhou

**Affiliations:** ^1^ State Key Laboratory of Rice Biology, Ministry of Agricultural and Rural Affairs Key Laboratory of Molecular Biology of Crop Pathogens and Insect Pests, Institute of Insect Sciences, Zhejiang University, Hangzhou, 310058, China.; ^2^ Hainan Institute, Zhejiang University, Sanya, 572000, China.; ^3^ College of Life and Environmental Sciences, Hangzhou Normal University, Hangzhou, China.; ^4^ Department of Economic Plants and Biotechnology, Yunnan Key Laboratory for Wild Plant Resources, Kunming Institute of Botany, Chinese Academy of Sciences, Kunming, 650201, China.; ^5^ The State Key Laboratory of Plant Trait Design, CAS Center for Excellence in Molecular Plant Sciences, Shanghai Institute of Plant Physiology and Ecology, Chinese Academy of Sciences, Shanghai, 200032, China.

## Abstract

Plants use two major defense strategies, resistance and tolerance, to counter herbivore attack; how these are coordinated remains poorly understood. Here, we demonstrate that damage by a specialist leafminer (*Phthorimaea operculella*) triggers tissue-specific responses in potato plants: compensatory growth in immature leaves, enabling tolerance, while resistance is induced in the mined leaf. The tissue-specific regulation of Sugars Will Eventually Be Exported Transporter 11 (SWEET11) mediates both responses. In unattacked sink leaves, SWEET11 is up-regulated, enhancing sugar import, promoting compensatory growth and herbivore tolerance. In contrast, SWEET11 is rapidly down-regulated in mined source leaves, inhibiting sugar export and increasing cellular sugar levels. The resulting sugar accumulations suppress SNF1-related protein kinase 1 (SnRK1) and thereby enhance herbivore resistance. SWEET11-mediated sugar signaling enhances jasmonic acid (JA)–mediated resistance by degrading MCPI1a and MCPI1b (metallocarboxypeptidase inhibitor 1), two JA-induced herbivory susceptibility factors. These findings reveal a dual role for sugar transport in regulating tissue-specific defense responses to herbivory.

## Teaser

Potato plants differentially regulate SWEET11 in sink and source leaves, which mediates two distinct responses to herbivory.

## INTRODUCTION

Resistance and tolerance are two major ecological strategies that all organisms use to combat biological threats (*1*, *2*) and are also the two primary defense responses that plants use to counter herbivory (*3*). Resistance refers to plant traits that minimize damage by herbivores, such as physical structures like thorns (*4*) and thick cuticles (*5*) that prevent herbivores from feeding and attaching, and chemical defenses, such as tannins (*6*) and alkaloids (*7*) that poison herbivores or thwart their assimilation of plant tissues. Tolerance refers to the ability of plants to regenerate or recover from herbivore damage (*8*). Originating from population genetic theory, the concept of plant tolerance was initially used by agronomists to describe yield losses of crops in response to pest damage and later adopted by ecologists to describe the reaction norms of specific plant genotypes to variations in herbivore load (*9*). Plants that tolerate herbivore damage use diverse ways of minimizing the fitness consequences of herbivore damage, for example, by redirecting damage to tissues with lower fitness value, such as senescent leaves, while simultaneously shifting resources to young tissues resulting in compensatory growth (*10*, *11*). Tolerance engages traits that influence whole-plant growth and the timing of life history events, such as when to reproduce (*12*), senesce (*13*), and enter dormancy (*14*). Hence, when compared with resistance traits, tolerance-related traits are not readily studied in high-throughput bioassays (*15*). However, when resistance and tolerance traits are understood at a molecular level and their expression can be manipulated genetically, in-depth investigations of their consequences for individual plant performance are possible.

Whole-plant carbohydrate mobilization (or reallocation), regulated by the well-known source (photoassimilate exporter)–sink (photoassimilate importer) theory (*16*), plays a central role in understanding the physiological mechanisms of herbivory tolerance (*17*). Two main patterns of carbohydrate reallocation have been observed in tolerance responses. First, in response to herbivores that are less mobile (leafminers and stem borers), plants tend to reallocate carbohydrate for compensatory growth during herbivore feeding. For instance, root attack by *Diabrotica virgifera virgifera* caused maize plants to allocate new carbon from source leaves to stems and roots, which was associated with increased growth of stem-born roots (*18*). Second, in response to voracious herbivores of high mobility, plants tend to reallocate carbohydrate for compensatory growth after herbivore feeding. For instance, attack by *Manduca sexta* led *Nicotiana attenuata* plants to move new carbon away from attacked sites to roots, preserving resources that allow plants to extend flower production or regrowth postattack (*11*, *19*). Similar mechanisms are likely at play in the iconic scarlet gilia plant (*Ipomopsis aggregata*), which overcompensates reproductively after ungulate browsers have moved to higher altitude feeding sites (*20*).

Carbohydrate mobilization is also implicated in plant defense response in attacked tissues by regulating sugar availability (*17*, *21*). The role of sugar mobilization in plant pathogen defense is becoming increasingly clear, suggesting that sugars function not only as energy or substrates for chemical defenses but also as signaling molecules in plant defense responses (*21*). In *Arabidopsis*, the activity of SUGAR TRANSPORTER PROTEIN 13 is directly enhanced by an immune receptor, which strengthens antibacterial defense by elevating cellular sugar levels (*22*). This process further coordinates pathogen defense signaling through sugar sensors including hexokinases (HXK) (*23*) and SNF1-related protein kinase 1 (SnRK1) (*24*). In response to herbivore attack, sugar transport activities in local source leaves are also altered, modulating sugar levels in attacked leaves (*17*). While evidence suggests that herbivory-induced changes in soluble sugars can modulate a plant’s herbivore resistance (*25*, *26*), the responsible mechanisms of this modulation remain largely unexplored.

Leaf-mining insects (leafminers), with more than 10,000 species identified globally, occupy a prominent position among insect herbivores due to their unique feeding behavior of consuming the internal tissues of plants (*27*, *28*). Evidence of leaf-mining dates to the Late Carboniferous period (~300 million years) (*29*). *Phthorimaea operculella* is a leafminer likely coevolved with the ancestors of potato (*30*). Despite the foliar damage caused by this pest, it rarely results in significant yield losses (*31*), reflecting potato’s tolerance of the attack of this leafminer. Here, we observed a tissue-specific defense response in potato plants to the attack of *P*. *operculella*: compensatory growth in unattacked immature leaves, enabling tolerance to herbivory, while resistance is induced in attacked leaves. A key sugar transporter, Sugars Will Eventually Be Exported Transporter 11 (SWEET11), was found to mediate this tissue-specific defense response. SWEET11 is regulated in a tissue-specific manner in potato’s source and sink tissues, which mediates carbohydrate reallocation and coordinates the resistance and compensatory responses. Furthermore, the interplay between sugar signaling and jasmonic acid (JA) signaling in attacked leaves, involving previously identified herbivory susceptibility factors MCPI1s (metallocarboxypeptidase inhibitor 1) (*32*, *33*), is highlighted. The work provides insights into plant defense against leafminers and establishes a mechanistic framework for synergies between different defense responses via source-sink regulation.

## RESULTS

### Potato plants deploy tissue-specific responses to *P. operculella* damage

**Fig. 1.**
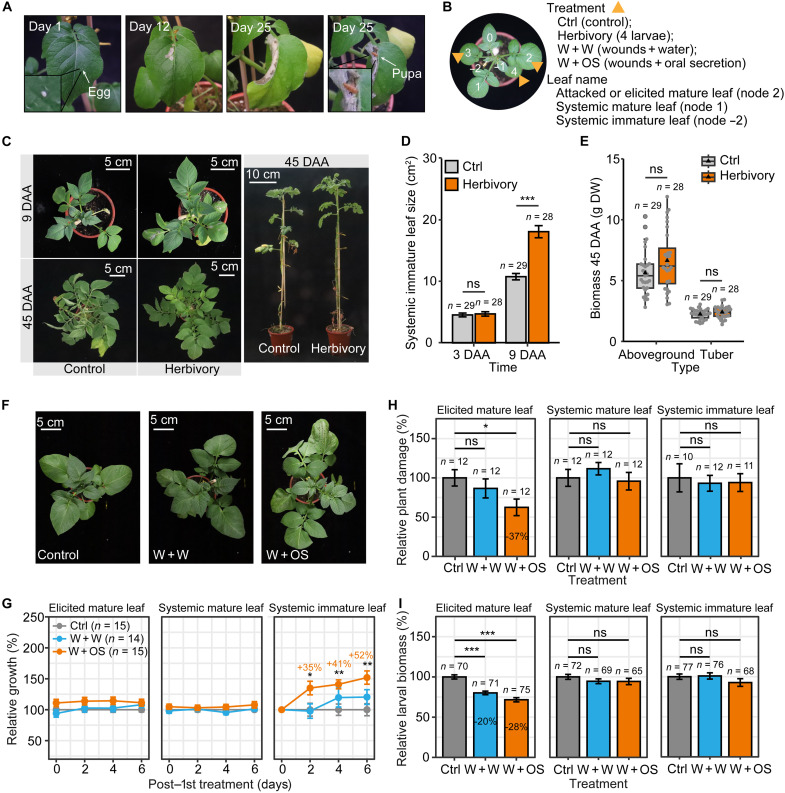
Local and systemic defense responses of potato plants to *Phthorimaea operculella* damage. (**A**) *P. operculella*’s larval stage occurs entirely within a single potato leaf. After oviposition on day 1, larval leaf mining is readily observed on day 12, and its larvae pupate within the leaf mine by day 25. (**B**) Plant treatments and leaf labeling. Leaves were numbered sequentially from youngest to oldest with node 0 undergoing the sink-source transition and node 1 designating the youngest mature leaf. Nodes 2, 3, and 4 of 28-day potato plants were treated as shown. (**C** to **E**) Potato plants compensate for herbivory. Herbivory of mature leaves (nodes 2, 3, and 4) promoted the growth of systemic immature leaves (node −2) 9 days after start of attacks (DAA; D; mean ± SEM, *n* = 28 to 29). Herbivory did not affect aboveground or tuber biomass 45 DAA (E; boxplot, *n* = 28 to 29). Triangle refers to the mean value, black circle refers to outlier, and gray point refers to data point. (**F** and **G**) Herbivory elicitation (W + OS) faithfully elicited plant responses to leafminers. W + OS of mature leaves (nodes 2, 3, and 4) induced compensatory growth in systemic immature leaves (node −2) 2, 4, and 6 days after the first treatment (G; mean ± SEM, *n* = 14 to 15). Representative plants were photographed at 6 days after the first treatment (F). (**H** and **I**) Herbivory elicitation (W + OS) induced resistance in elicited mature leaves. W + OS reduced leaf damage (in square centimeters) by larvae in elicited mature leaves (node 2) (H; mean ± SEM, *n* = 10 to 12). W + W and W + OS reduced larval biomass in bioassays performed on elicited mature leaves (I; mean ± SEM, *n* = 65 to 77). Asterisks indicate significant difference between treatment and control groups (Student’s *t* test or Mann-Whitney *U* test, *P < 0.05, **P < 0.01, and ***P < 0.001; ns, not significant). DW, dry weight; FW, fresh weight.

As is typical for leafminers, *P. operculella*’s larval stages are mostly completed entirely within an individual potato leaf (Fig. 1A). We observed significant changes in the growth of potato plants in response to the damage of *P. operculella* (Fig. 1C). *P. operculella* attack rapidly induced compensatory growth in systemic immature leaves at 9 days after the start of damage (Fig. 1, C and D), while it did not affect the biomass of both the aboveground parts and tubers at 45 days after the start of damage (Fig. 1E). We infer that this compensatory growth is induced by the herbivore damage signals because herbivory elicitation [mechanical wounds (W) + larval oral secretions (OS), W + OS] to mature leaves also induces compensatory growth (by up to 52%) in systemic immature leaves (Fig. 1, F and G). Moreover, herbivory stimulations suppress root growth (fig. S1, A and B), indicating that this compensatory shoot growth occurs at the expense of root growth.

To determine whether *P. operculella* damage also elicits resistance traits in addition to inducing compensatory growth, we elicited plants with *P. operculella* herbivory and conducted larval bioassays on different leaf types. Herbivory elicitation (W + OS) to mature leaves significantly reduced leaf damage (up to 37.5 ± 10.1%, *P* = 0.035) and larval growth (up to 28.4 ± 2.6%, *P* = 8.7 × 10^−12^), while it did not affect resistance traits in systemic mature and immature leaves of the same plants (Fig. 1, H and I). Moreover, the accumulation of JA and JA-Ile, key defense phytohormones, in these leaf types showed a consistent increase to leaf damage and larval OS (fig. S2, B and C). From these data, we infer that potato plants use tissue-specific responses against *P. operculella* damage that coordinate compensatory growth in systemic immature leaves with induced resistance responses in attacked mature leaves.

### Sugar transport is involved in the tissue-specific defense responses

**Fig. 2.**
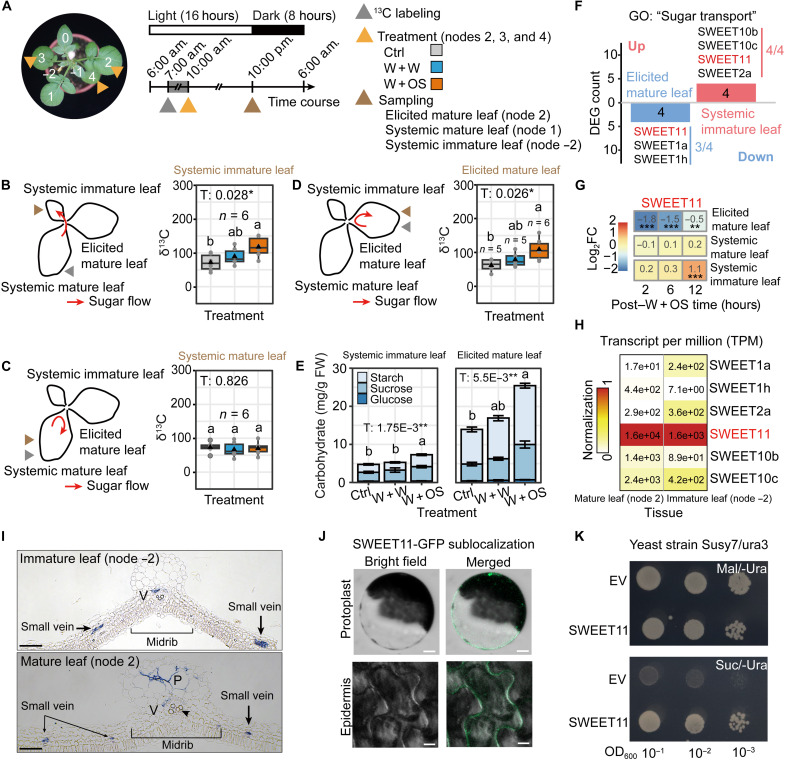
Potato plants likely differentially regulate sugar transport and a key sugar transporter SWEET11 following herbivory elicitation. (**A**) Schematic of ^13^C labeling experimental time course. A mature leaf was ^13^C-labeled before treatments, and ^13^C levels (δ^13^C) of target node were quantified after sampling. (**B** and **C**) W + OS of mature leaves (nodes 2, 3, and 4) increased systemic mature leaf (node 1)–derived ^13^C in systemic immature leaves (node −2) (B; boxplot, *n* = 6) but did not affect ^13^C in systemic mature leaves (C; boxplot, *n* = 6). (**D**) When elicited mature leaf (node 2) was ^13^C-labeled before W + OS, retained ^13^C increased (boxplot, *n* = 5 to 6). (**E**) W + OS of mature leaves (nodes 2, 3, and 4) increased carbohydrates in both systemic immature leaf (node −2) and elicited mature leaf (node 2) 12 hours after treatment (mean ± SEM, *n* = 5). (**F** to **H**) RNA-seq reveals the tissue-specific regulation of SWEET11. Differentially expressed gene (DEG) analysis identified SWEET genes responsive to W + OS in mature leaves (nodes 2, 3, and 4) (F). SWEET11 was up-regulated in systemic immature leaves (node −2) and down-regulated in elicited mature (node 2) leaves (G). Asterisks indicate DEG significance (**Padj < 0.01 and ***Padj < 0.001). Transcript abundances of OS-elicited SWEET genes in both mature and immature leaves (H). GO, gene ontology; FC, fold change. (**I**) In situ hybridization of SWEET11 mRNA in cross sections of immature leaf (node −2) and mature leaf (node 2). Scale bar, 100 μm. P, parenchyma-like; V, vasculature. (**J**) Sublocalization of SWEET11-GFP in planta. Scale bars, 10 μm. (**K**) SWEET11 restored growth of sucrose transport–deficient yeast in sucrose (Suc) medium. Mal, maltose; Ura, uracil; EV, empty vector. In boxplot, triangle and gray point respectively refer to mean value and data point. Different letters indicate significant differences between treatments [least significant difference (LSD) post hoc multiple comparisons following one-way analysis of variance (ANOVA), *P < 0.05, **P < 0.01, and ***P < 0.001]. Experiment (K) was repeated independently twice with similar results.

Previous studies suggest that compensatory or tolerance responses to herbivory are linked to resource allocation (*11*, *18*). We used stable isotope (^13^C) tracing to investigate whether potato plants reallocate sugars in response to *P. operculella* damage (Fig. 2A). Systemic mature leaves were ^13^C-labeled before treatment. Herbivory elicitation (W + OS) significantly increased δ^13^C levels in systemic immature leaves, but not in systemic mature leaves, at 12 hours posttreatment (Fig. 2, B and C), indicating enhanced sugar import into the former. Unexpectedly, herbivory elicitation (W + OS) also inhibited sugar export from the elicited mature leaves, as evidenced by significant increases in their δ^13^C levels (Fig. 2D). Moreover, changes in total carbohydrate content corresponded with the δ^13^C shifts, because carbohydrate levels in both systemic immature leaves and elicited mature leaves increased 12 hours post–W + OS treatments of mature leaves (Fig. 2E). In addition, W + OS treatment of mature leaves resulted in decreased root carbohydrate contents, indicating reduced sugar allocation to roots (fig. S1, C to E). These results suggest that potato plants use tissue-specific sugar transport responses that allocate sugars to different tissues in response to *P. operculella* damage.

To understand how potato plants regulate tissue-specific sugar transport, we analyzed the expression of genes in different tissues following herbivory elicitation (W + OS) treatments using RNA sequencing (RNA-seq; fig. S2, D to F, and data S2). The key genes for sugar transport, the SWEET genes, were down-regulated in elicited mature leaves and up-regulated in systemic immature leaves following herbivory elicitation (W + OS) (Fig. 2F). Among these, the transcripts of SWEET11 were dominantly expressed in both leaf types (Fig. 2, G and H, and figs. S3 and S4). In situ hybridization of SWEET11 mRNA also suggested its roles of sugar transport in both immature and mature leaves (Fig. 2I and fig. S5). In mature leaves (node 2), SWEET11 is expressed in the vasculature (V), which suggested its role in phloem sugar loading in mature leaves (*34*, *35*). SWEET11 is also expressed in parenchyma (P)–like tissues (large cells lacking chloroplast; fig. S5A) adjacent to the vasculature in midribs. In immature leaves (node −2), SWEET11 mRNA is primarily expressed in the vasculature, consistent with its role in phloem sugar unloading in distant tissues (*36*). In addition, the fusion protein SWEET11–green fluorescent protein (GFP) was predominantly localized to cell membranes (Fig. 2J), and expression of SWEET11 in the sucrose transport–deficient yeast strain, Susy7/ura3, restored growth on sucrose media, consistent with SWEET11’s function in sucrose transport (Fig. 2K). From these results, we infer that SWEET11 is involved in sugar export in mature leaves and sugar import into immature leaves in potato plants damaged by *P. operculella* larvae.

### SWEET11 mediates sugar import and compensation in sink leaves

**Fig. 3.**
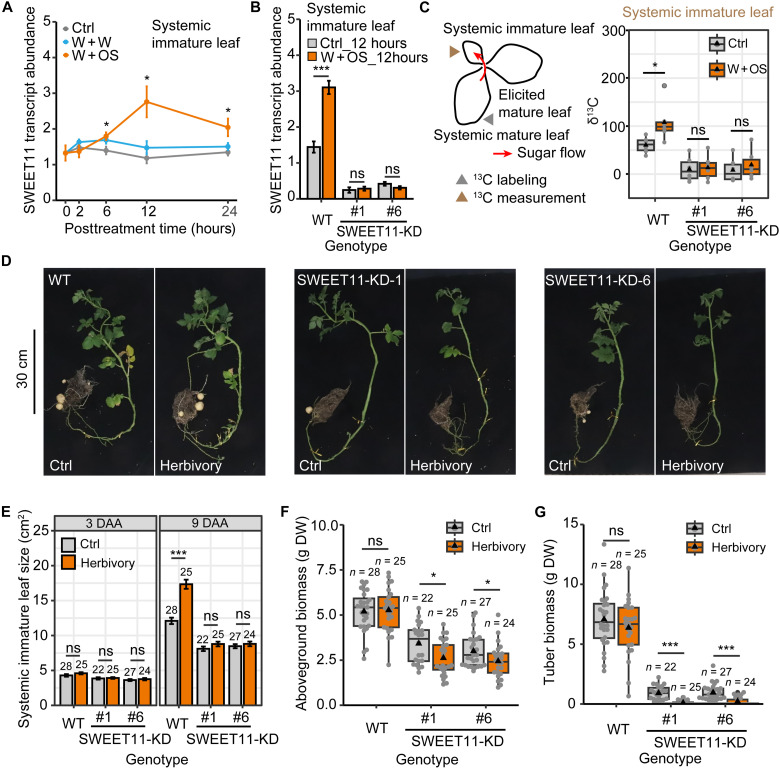
The roles of SWEET11 in sugar import and growth compensation (tolerance) in systemic immature leaf following herbivory elicitation. (**A**) qRT-PCR verification of SWEET11 transcriptional responses in immature leaves (node −2) following W + W and W + OS treatments of mature leaves (nodes 2, 3, and 4; mean ± SEM, *n* = 4 to 5). (**B** and **C**) SWEET11 mediated sugar import into immature leaves following herbivory elicitation (W + OS) of mature leaves. In SWEET11-KD plant, herbivory elicitation (W + OS) of mature leaves (nodes 2, 3, and 4) failed to up-regulate SWEET11 transcript abundance in systemic immature leaves (node −2) 12 hours after elicitation (B; mean ± SEM, *n* = 4). In SWEET11-KD plant, herbivory elicitation of mature leaves did not increase systemic mature leaves (node 1)–derived ^13^C in systemic immature leaves 12 hours after elicitation (C; boxplot, *n* = 6). (**D** to **G**) SWEET11-KD impaired potato plant’s tolerance of *P. operculella* herbivory. Representative plants (D) were photographed 45 days after the start of herbivory (nodes 2, 3, and 4 attacked by four larvae respectively). Herbivory failed to induce compensatory growth in immature leaves (node −2) of SWEET11-KD plants 9 days after the start of attacks (E; mean ± SEM; *n* = 22 to 28, *n* was displayed on bars). In addition, in contrast to WT plants, herbivory on SWEET11-KD plants resulted in biomass loss in both aboveground parts (F) and tubers (G) 45 days after the start of herbivory (boxplot, *n* = 22 to 28). In boxplot, triangle refers to mean value, and gray point refers to data point. Asterisks indicate significant difference between the treatment and control groups (Student’s *t* test or Mann-Whitney *U* test, *P < 0.05 and ***P < 0.001). Experiments (F) and (G) were repeated independently twice with similar results.

Quantitative reverse transcription polymerase chain reaction (qRT-PCR) analysis confirmed that W + OS treatment of mature leaves induced SWEET11 transcript accumulations in systemic immature leaves (Fig. 3A). To explore the role of SWEET11 in the observed regulation of sugar transport and compensatory growth, we knocked down its expression using RNA interference (RNAi; fig. S6) and ectopically overexpressed it with a 35*S* promotor (fig. S7). In SWEET11–knockdown (KD) plants, SWEET11 transcript levels were constitutively repressed in systemic immature leaves with or without herbivory elicitation (W + OS) (Fig. 3B). In ^13^C tracing assays, herbivory elicitation (W + OS) of mature leaves failed to increase sugar import into systemic immature leaves in SWEET11-KD plants (Fig. 3C). In SWEET11–overexpression (OE) plants, SWEET11 transcript levels were constitutively elevated, but its induction in systemic immature leaves was either weakened or not significant, and sugar import into systemic immature leaves was not significantly changed by W + OS treatment of mature leaves (fig. S8, A and B). These results suggested that SWEET11 mediates enhanced sugar import into systemic immature leaves following herbivory elicitation of mature leaves.

Consistently, *P. operculella* damage on mature leaves induced compensatory growth in systemic immature leaves in wild-type (WT) plants but not in SWEET11-KD plants (Fig. 3E). In addition, *P. operculella* damage resulted in biomass losses both in the aboveground parts (Fig. 3F) and tubers (Fig. 3G) in SWEET11-KD plants, but not in WT plants (Fig. 3D). From these results, we infer that SWEET11 mediates compensatory growth in systemic immature leaves following *P. operculella* herbivory.

### SWEET11 regulates sugar export and resistance in source leaves

**Fig. 4.**
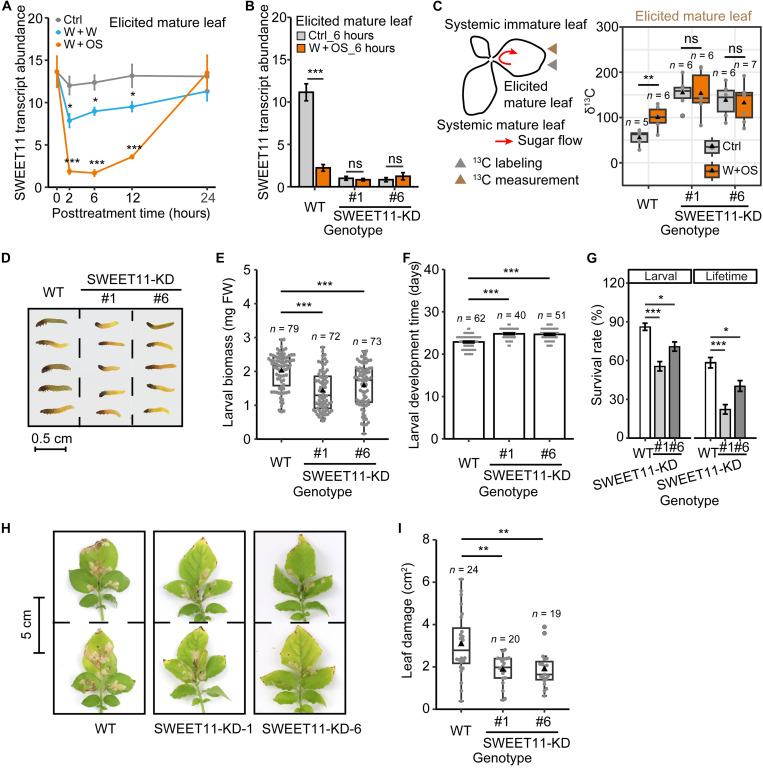
The impacts of SWEET11 repression on sugar export and herbivore resistance in mature leaves. (**A**) qRT-PCR verification of SWEET11 transcriptional response in mature leaf (node 2) to W + W or W + OS treatments (mean ± SEM, *n* = 4). (**B** and **C**) SWEET11 mediated sugar export in elicited mature leaves following herbivory elicitation (W + OS). In SWEET11-KD plants, herbivory elicitation (W + OS) failed to repress SWEET11 relative transcript accumulations in elicited mature leaves (node 2) 6 hours after elicitation (B; mean ± SEM, *n* = 4). Consequently, sugar export of mature leaves was constitutively inhibited in SWEET11-KD plants, and herbivory elicitation (W + OS) did not further inhibit its sugar export in SWEET11-KD plants within 12 hours after elicitation (C; boxplot, *n* = 5 to 7). (**D** to **I**) SWEET11-KD enhanced resistance in mature leaves. Representative larvae from bioassays performed on SWEET11-KD plants (D). SWEET11-KD reduced larval biomass (E; boxplot, *n* = 72 to 79), prolonged larval development time (F; mean ± SEM, *n* = 40 to 62), and decreased survival rates (G; mean ± SEM, *n* = 8). Representative leaves of different genotypes damaged by *P. operculella* larvae (H). SWEET11-KD reduced leaf damage after 12-day *P. operculella* herbivory (I; boxplot, *n* = 19 to 24). In boxplot, triangle refers to mean value, and gray point refers to data point. Asterisks indicate significant difference between treatments or genotypes (Student’s *t* test or Mann-Whitney *U* test, *P < 0.05, **P < 0.01, and ***P < 0.001). Experiment (E) was repeated independently twice with similar results.

qRT-PCR analysis confirmed that SWEET11 transcript abundance is rapidly down-regulated (by up to 86.4 ± 2.5%, *P* = 2.5 × 10^−5^) in W + OS–treated mature leaves (Fig. 4A). The transcriptional responses of SWEET11 to herbivory elicitation (W + OS) in different tissues are independent of JA signaling, as evidenced by their unaltered responses in plants silenced in allene oxide cyclase (AOC) expression (fig. S9). To explore the role of SWEET11 in herbivore resistance in attacked leaves, we assessed its function for sugar export and herbivore resistance in the mature leaves of SWEET11-KD plants. SWEET11 transcript levels in mature leaves are constitutively repressed in SWEET11-KD plants with or without herbivory elicitation (W + OS) (Fig. 4B). Moreover, SWEET11-KD plants showed higher δ^13^C levels in mature leaves than those in the WT plants with or without W + OS treatments, and the characteristic W + OS elicited an increase in δ^13^C levels observed in WT plants was absent in SWEET11-KD plants (Fig. 4C).

Feeding on SWEET11-KD plants significantly reduced *P. operculella* larval biomass (Fig. 4, D and E) and prolonged larval development (Fig. 4F). Moreover, when fed SWEET11-KD plants, both larval survival and lifetime survival rates were significantly reduced (Fig. 4G). In addition, SWEET11-KD significantly reduced the amount of leaf damage from *P. operculella* (Fig. 4, H and I). From these results, we infer that repression of SWEET11 enhances herbivore resistance in mature leaves.

### SWEET11-mediated sugar accumulation enhances herbivore resistance in attacked leaves

**Fig. 5.**
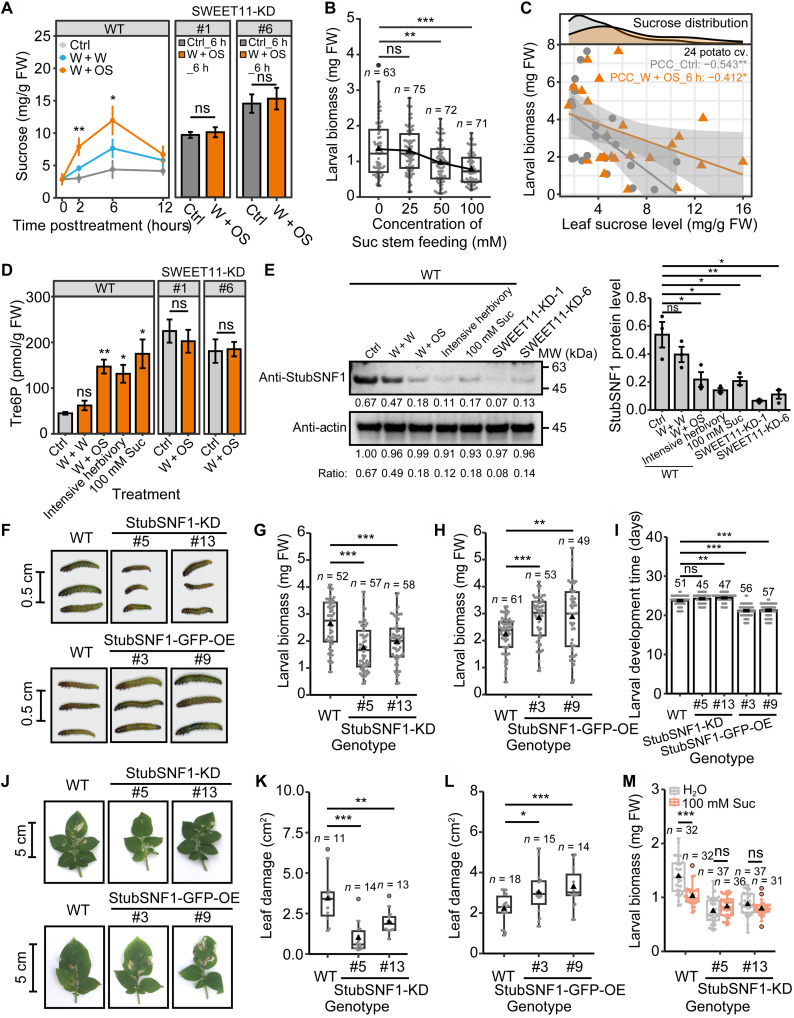
SWEET11-mediated sugar accumulation enhances herbivore resistance by suppressing sugar sensor, SnRK1α (StubSNF1). (**A**) W + OS rapidly elevated leaf sucrose in mature leaves (node 2) through SWEET11 (mean ± SEM, *n* = 5). h, hours. (**B**) Exogenous sucrose (Suc) reduced larval performance inmature (nodes 1 and 2) leaves (boxplot, *n* = 63 to 75). (**C**) Pearson correlation coefficient (PCC) between mature leaf (node 2) sucrose levels (*n* = 4 to 5) and larval biomass (*n* = 30 to 71) (*P < 0.05 and **P < 0.01; ribbons indicate 95% confidence intervals). (**D**) Herbivory elicitation elevated mature leaf (node 2) trehalose-6-phophate (Tre6P) through SWEET11 (mean ± SEM, *n* = 5). Tre6P was measured 6 hours after W + OS in WT and SWEET11-KD plants and 6 hours after intensive herbivory (30 fourth-instar larvae feeding for 1 hour) or sucrose supplementation (100 mM Suc) in WT plants. (**E**) Herbivory elicitation suppressed StubSNF1 through SWEET11 in mature leaves (node 2). StubSNF1 protein was quantified by immunoblotting 6 hours after herbivory elicitation treatments in WT plants and in untreated SWEET11-KD plants. Left: Representative image. Right: Quantification (mean ± SEM, *n* = 3). StubSNF1 negatively regulates herbivore resistance. MW, molecular weight. Representative larvae (**F**) and leaves (**J**) from bioassays on different genotypes. StubSNF1-KD reduced larval biomass (**G**; boxplot, *n* = 52 to 58) and leaf damage (**K**; boxplot, *n* = 11 to 14) after herbivory. StubSNF1 overexpression increased larval biomass (**H**; boxplot, *n* = 49 to 61), accelerated larval development (**I**; mean ± SEM, *n* = 45 to 57), and increased leaf damage (**L**; boxplot, *n* = 14 to 18) after herbivory. (**M**) Exogenous sucrose (100 mM Suc) did not reduce larval performance in StubSNF1-KD mature leaves (nodes 1 and 2) as it did in WT mature leaves (boxplot, *n* = 31 to 37). In boxplot, triangle and gray point respectively refer to mean value and data point. Asterisks indicate significant difference between treatments or genotypes (Student’s *t* test or Mann-Whitney *U* test, *P < 0.05, **P < 0.01, and ***P < 0.001). Experiments (G) and (H) were repeated independently twice with similar results.

SWEET11 mediates a marked increase in carbohydrate contents (of both starch and soluble sugars) in mature leaves following herbivory elicitation (W + OS) (Fig. 5A and figs. S10A and S11). Previous studies have shown that an imbalanced protein-to-carbohydrate ratio in diets negatively affects caterpillar growth and development (*26*). To determine whether the repression of SWEET11 enhances herbivore resistance by inducing dietary imbalances in *P. operculella* larvae, we conducted larval bioassays with semiartificial diets (semi-ADs) prepared from mature leaves of WT and SWEET11-KD plants and by supplementing these diets with additional sugars. *P. operculella* larvae showed similar survival rates and weight gains after feeding on different semi-ADs within each treatment group, indicating that increasing carbohydrate content in the diet does not affect the performance of *P. operculella* larvae (fig. S10).

Recent studies have shown that sugars serve not only as energy sources and biosynthetic substrates but also as signals that modulate plant defense responses (*23*). To evaluate whether sugar affects the herbivore defense response in potato plants, we supplemented sugars to mature leaves by stem feeding and conducted larval bioassays. Sucrose supplementation to mature leaves significantly reduced larval biomass (Fig. 5B). In addition, sucrose levels in mature leaves were negatively correlated with larval performance (larval biomass) across 24 potato cultivars [Fig. 5C; Pearson correlation coefficient (PCC)]. Similarly, hexose (glucose and fructose) supplementation in stem feeding assays also reduced larval performance, and leaf hexose levels showed negative correlations with larval performance (fig. S12). From these data, we inferred that SWEET11-mediated sugar accumulations in mature leaves enhance herbivore resistance.

### Sugar enhances herbivore resistance through StubSNF1 in attacked leaves

Plants sense their cellular sugar status and trigger a cascade of physiological responses through sugar signaling pathways, in which trehalose-6-phosphate (Tre6P) functions as a key proxy for cellular sugar levels (*37*). In WT plants, herbivory elicitation (W + OS), intensive herbivory (30 fourth-instar larvae feeding for 1 hour), and sucrose supplementation all significantly increased Tre6P levels in mature leaves at 6 hours posttreatment (Fig. 5D). In SWEET11-KD plants, Tre6P levels were constitutively elevated in mature leaves, and herbivory elicitation (W + OS) did not further alter Tre6P levels, indicating that SWEET11 plays a critical role in regulating cellular sugar accumulation levels (Fig. 5D).

SnRK1 is a conserved sugar sensor that plays a pivotal role in sugar signaling pathway in planta (*24*). In potato, StubSNF1 is the closest ortholog of *Arabidopsis* SnRK1α (AKIN10), exhibiting high sequence conservation, particularly in the catalytic kinase domain (fig. S13). In WT plants, herbivory elicitation (W + OS), intensive herbivory, or sucrose supplementation all significantly decreased StubSNF1 protein levels in mature leaves at 6 hours posttreatment (Fig. 5E), whereas these treatments all increased cellular sugar accumulations (Fig. 5D). However, in SWEET11-KD plants, StubSNF1 protein levels were constitutively repressed (Fig. 5E). Furthermore, herbivory elicitation (W + OS) reduced StubSNF1 transcript levels and protein abundances through the action of SWEET11 (fig. S14); herbivory elicitation inhibited StubSNF1 activation in part through elevated sugar levels (fig. S15). These findings demonstrate that herbivory elicitation elevates cellular sugar status, amplifies sugar signaling, and suppresses StubSNF1 signaling via SWEET11 regulation.

To investigate the role of StubSNF1 in herbivore resistance, we constructed StubSNF1-KD (fig. S16) and StubSNF1-GFP-OE (fig. S17) plants and conducted larval bioassays with these plants. Compared to larvae feeding on WT plants, larvae feeding on StubSNF1-KD plants had reduced biomass (Fig. 5, F and G), while those feeding on StubSNF1-OE plants had increased biomass (Fig. 5, F and H). Larvae feeding on StubSNF1-OE plants had significantly accelerated development rates (Fig. 5I). In addition, compared to WT plants, StubSNF1-KD plants had reduced amounts of leaf damage, while StubSNF1-OE plants were damaged more heavily (Fig. 5, J to L). In contrast to WT leaves, exogenous sugar did not reduce larval biomass in StubSNF1-KD leaves (Fig. 5M), consistent with the inference that sugar enhances herbivore resistance through the action of StubSNF1. These results suggested that StubSNF1 mediates sugar-induced herbivore resistance in *P. operculella*–damaged mature leaves.

### StubSNF1 regulates two herbivore susceptibility factors, MCPI1a and MCPI1b

**Fig. 6.**
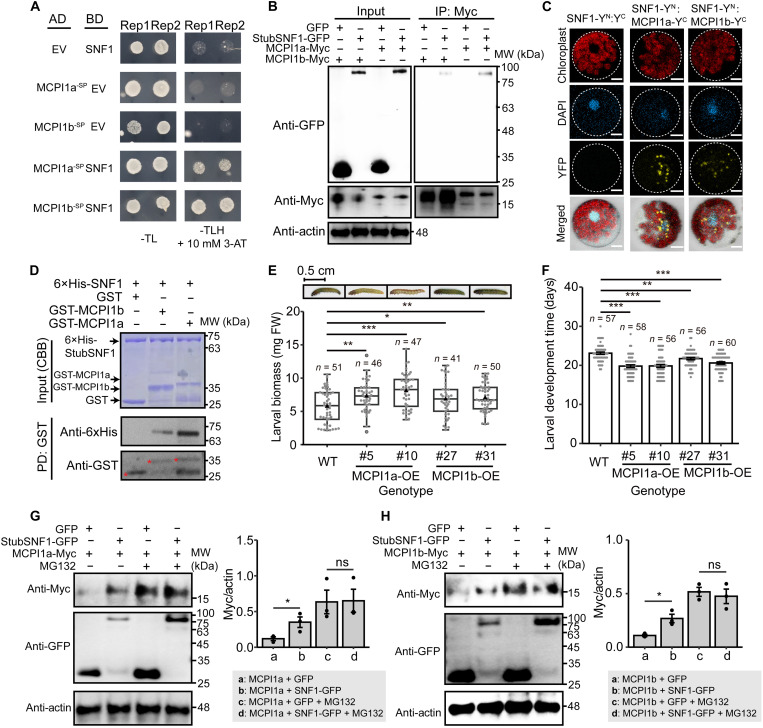
StubSNF1 stabilizes two herbivore susceptibility factors, MCPI1a and MCPI1b. (**A**) StubSNF1 interacted with MCPI1a and MCPI1b in Y2H assays. -SP, N-terminal signal peptide removed. (**B**) Co-IP assay was performed to investigate interaction between StubSNF1 and MCPI1a/1b in planta. Co-IP was performed in *N. benthamiana* protoplasts with StubSNF1-GFP and MCPI1a/1b-Myc coexpression. Proteins were detected by immunoblotting. (**C**) Confocal images of BiFC assays revealed that StubSNF1 interacts with MCPI1a and MCPI1b in planta. Scale bars, 10 μm. YFP: signals from BiFC. 4′,6-Diamidino-2-phenylindole (DAPI): signals from DNA. (**D**) GST-pulldown (PD:GST) assays were performed to evaluate the interaction between StubSNF1 and MCPI1a/1b in vitro. The 6×His-StubSNF1 protein was mixed with either GST (negative control) or GST-MCPI1a/1b and incubated with GST agarose beads. Input proteins were visualized by Coomassie blue (CBB) staining after mixing. Proteins were detected by immunoblotting after pulldown. (**E** and **F**) MCPI1a and MCPI1b negatively regulated herbivore resistance. MCPI1a/1b overexpression significantly increases larval biomass (E; boxplot, *n* = 41 to 51; top: representative larvae) and accelerated larval development (F; mean ± SEM, *n* = 56 to 60). In boxplot, triangle and gray point respectively refer to mean value and data point. Asterisks indicate significant difference between genotypes (Student’s *t* test or Mann-Whitney *U* test, *P < 0.05, **P < 0.01, and ***P < 0.001). (**G** and **H**) StubSNF1 stabilizes MCPI1a and MCPI1b in planta. MCPI1a-Myc (G) and MCPI1b-Myc (H) proteins were analyzed by immunoblotting after coexpression with StubSNF1-GFP for 72 hours in *N. benthamiana* leaf, with or without treatment of 26*S* proteasome inhibitor MG132. Coexpression with GFP served as the control group, and actin served as an internal standard. Left: Representative image. Right: Quantification (mean ± SEM, *n* = 3). Asterisks indicate significant difference between StubSNF1 and GFP groups (Student’s *t* test, *P < 0.05, **P < 0.01, and ***P < 0.001). SNF1, StubSNF1. Experiments (A) to (E) were repeated independently twice with similar results.

Given that StubSNF1 can regulate herbivore resistance in *P. operculella*–damaged mature leaves, we screened for StubSNF1 interacting factors using yeast two-hybrid (Y2H) library screening. Among the identified candidate interacting factors (see data S3), the MCPI showed a strong interaction with StubSNF1. Genome-wide identification revealed at least five MCPI genes in the potato genome, with two paralogs of solanaceae-specific MCPI1 predominantly expressed in mature leaves (fig. S18). Y2H assays confirmed that StubSNF1 can interact with MCPI1a and MCPI1b in yeast when their signal peptide sequences are removed (Fig. 6A). Coimmunoprecipitation (co-IP) assays also demonstrated that StubSNF1 was immunoprecipitated with MCPI1a/1b in *Nicotiana benthamiana* leaf protoplasts (Fig. 6B). In bimolecular fluorescence complementation (BiFC) assays, yellow fluorescent protein (YFP) signals were observed outside the nuclear region when coexpressing StubSNF1-nYFP and MCPI1a/1b-cYFP (Fig. 6C). These results demonstrated that StubSNF1 interacts with MCPI1a/1b in plant cells. In addition, glutathione *S*-transferase (GST)–pulldown assays showed a direct interaction in vitro (Fig. 6D). These findings indicate that StubSNF1 interacts with MCPI1a and MCPI1b both in vivo and in vitro.

Potato MCPI1a, which encodes the precursor of potato carboxypeptidase inhibitor, has previously been identified as an herbivore susceptibility factor. Its heterologous expression in tomato or rice significantly compromises herbivore resistance (*32*, *33*). To examine its role in potato, we overexpressed MCPI1a/1b in potato plants (fig. S19). Overexpression of either MCPI1a or MCPI1b significantly increased larval biomass (Fig. 6E) and accelerated the development of larvae feeding on these plants (Fig. 6F). These findings demonstrate that both MCPI1a and MCPI1b impair herbivore resistance in potato plants.

Previous research in tomato suggested that MCPI1 undergoes robust posttranslational regulation. Leaf wounding induces a marked increase in MCPI1 mRNA levels but without a concomitant rise in mature MCPI1 protein (*38*). To investigate whether StubSNF1 regulates MCPI1a and MCPI1b at the posttranslational level, we performed assays in *N*. *benthamiana* leaves by coexpressing MCPI1a/1b with either StubSNF1-GFP or GFP. Coexpression of StubSNF1 significantly increased protein levels of both MCPI1a-Myc and MCPI1b-Myc at 72 hours post–*Agrobacterium tumefaciens* infiltration (Fig. 6, G and H). However, treatment with the 26*S* proteasome inhibitor MG132 led to a constitutive increase in the protein levels of MCPI1a-Myc and MCPI1b-Myc, with coexpression of StubSNF1 failing to further elevate these levels (Fig. 6, G and H). In addition, cell-free degradation assays reveal that recombinant MCPI1a and MCPI1b proteins degraded more rapidly in protein extracts from StubSNF1-KD mature leaves (fig. S20). From these results, we infer that StubSNF1 promotes the stability of MCPI1a and MCPI1b.

### JA induces transcript accumulations of MCPI1a/1b in attacked leaves

**Fig. 7.**
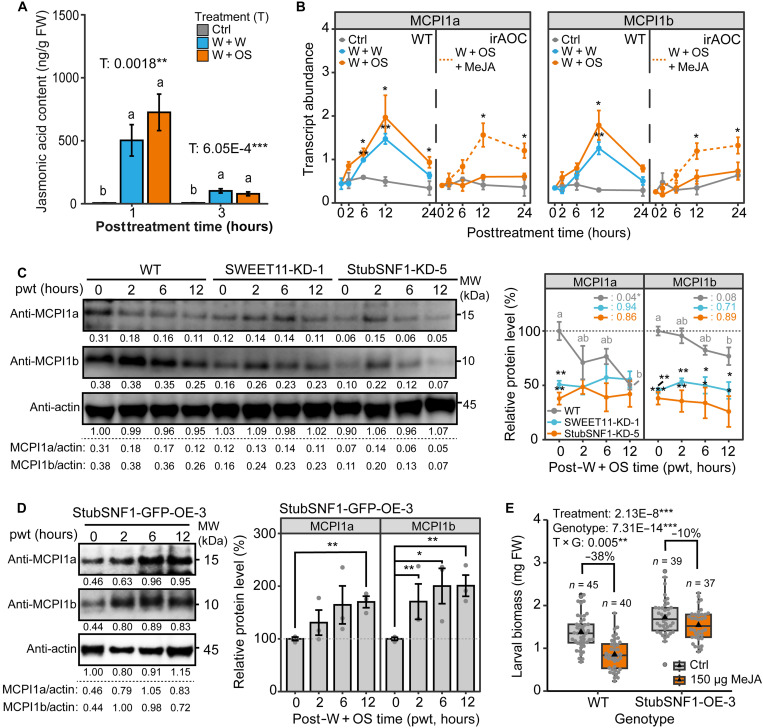
Herbivory-induced sugar signaling cooperates with JA signaling by degrading MCPI1a and MCPI1b. (**A**) W + W and W + OS induced JA accumulations of mature leaves (node 2) 1 and 3 hours after treatments (mean ± SEM, *n* = 5). Different letters indicate significant difference between treatments (LSD post hoc multiple comparisons following one-way ANOVA, *P < 0.05, **P < 0.01, and ***P < 0.001). (**B**) MCPI1a/1b transcript abundances were induced by JA signaling following herbivory elicitation. MCPI1a/1b transcript abundances of mature leaves (node 2) were measured within 24 hours after W + W and W + OS treatments in WT or JA biosynthesis–deficient (irAOC) plant (mean ± SEM, *n* = 3). MeJA was applied to irAOC leaves to complement JA deficiencies. (**C**) Plants degraded MCPI1a/1b proteins following herbivory elicitation though sugar signaling. MCPI1a/1b proteins of mature leaves (node 2) were measured by immunoblotting within 12 hours following W + OS in WT, SWEET11-KD-1, and StubSNF1-KD-5 plants, with actin as an internal standard. Left: Representative image. Right: Quantification (mean ± SEM, *n* = 3). Asterisks on error bars indicate significant difference between WT and KD plants at each time point after W + OS elicitation. Different letters indicate significant difference between time points in WT plants (LSD post hoc multiple comparisons following one-way ANOVA, *P < 0.05, **P < 0.01, and ***P < 0.001). (**D**) MCPI1a/1b proteins of mature leaves (node 2) were increased within 12 hours following W + OS in StubSNF1-GFP-OE-3 plants. Left: Representative image. Right: Quantification (mean ± SEM, *n* = 3). Asterisks indicate significant difference between treatment and control (time 0). (**E**) StubSNF1-GFP-OE-3 mature leaves (nodes 1 and 2) showed weaker herbivore resistance enhancement in response to MeJA compared to WT leaves (two-way ANOVA, *P < 0.05, **P < 0.01, and ***P < 0.001). pwt, Post-W + OS time. Triangle and gray point respectively refer to mean value and data point. Student’s *t* test was used for two-group comparisons (*P < 0.05, **P < 0.01, and ***P < 0.001).

JA is a crucial phytohormone in plant’s responses to biotic stresses (*39*, *40*). Our previous study has shown that silencing JA biosynthesis reduces leafminer resistance in potato plants (*41*). Here, we found that JA signaling induced transcript accumulations of two herbivore susceptibility factors, MCPI1a and MCPI1b, in W + OS–treated mature leaves. In WT plants, W + OS rapidly increased JA levels (Fig. 7A) and MCPI1a and MCPI1b transcript accumulations (Fig. 7B) in mature leaves. In JA biosynthesis–deficient [inverted repeat Allene Oxide Cyclase (irAOC)] plants, however, W + OS treatment did not induce transcript accumulations of MCPI1a and MCPI1b (Fig. 7B). Moreover, supplementation with methyl jasmonate (MeJA) to mature leaves restored the induction of MCPI1a and MCPI1b transcripts following W + OS in irAOC plants (Fig. 7B). These results suggest that JA induces the accumulations of MCPI1a and MCPI1b transcripts in herbivory-elicited leaves.

### Sugar signaling enhances JA-mediated resistance by degrading MCPI1a/1b

Given that JA signaling induces MCPI1a/1b transcript accumulations in herbivory-elicited leaves, whereas the sugar sensor, StubSNF1, regulates the stability of MCPI1a/1b proteins, we hypothesized that sugar signaling and JA signaling interact when *P. operculella* larvae damage leaves. To test this hypothesis, we first measured the changes of MCPI1a and MCPI1b protein levels within 12 hours post–herbivory elicitation, when their transcript abundances peak. Unexpectedly, in WT plants, W + OS treatment did not increase but rather decreased MCPI1a and MCPI1b protein levels (Fig. 7C). In SWEET11-KD and StubSNF1-KD plants, in which sugar signaling is amplified, MCPI1a and MCPI1b protein levels were constitutively reduced, and herbivory elicitation did not further decrease their levels (Fig. 7C). In addition, inhibition of bovine carboxypeptidase A (CPA) by leaf extracts closely mirrored the changes in MCPI1a and MCPI1b protein levels observed following W + OS treatment (fig. S21). These results demonstrate that herbivory elicitation induces the degradation of MCPI1a and MCPI1b through SWEET11-mediated sugar signaling.

Next, we used StubSNF1-OE plants and MeJA treatments to investigate the interaction between induced sugar and JA signaling in mediating herbivore resistance. In StubSNF1-OE plants, W + OS treatment resulted in accumulations of MCPI1a and MCPI1b proteins (Fig. 7D), accompanied by a concomitant increase in CPA inhibition by leaf extracts (fig. S21). In addition, a significant interactive effect between treatment and genotype (**P = 0.005) indicated that overexpression of StubSNF1 significantly reduced the effects of MeJA on larval performance (Fig. 7E). In WT plants, MeJA treatment reduced larval biomass by 38%, whereas with StubSNF1-OE plants, MeJA treatment reduced larval biomass by only 10% (Fig. 7E). From these results, we infer that StubSNF1 attenuates JA-mediated herbivore resistance in potato plants.

## DISCUSSION

**Fig. 8.**
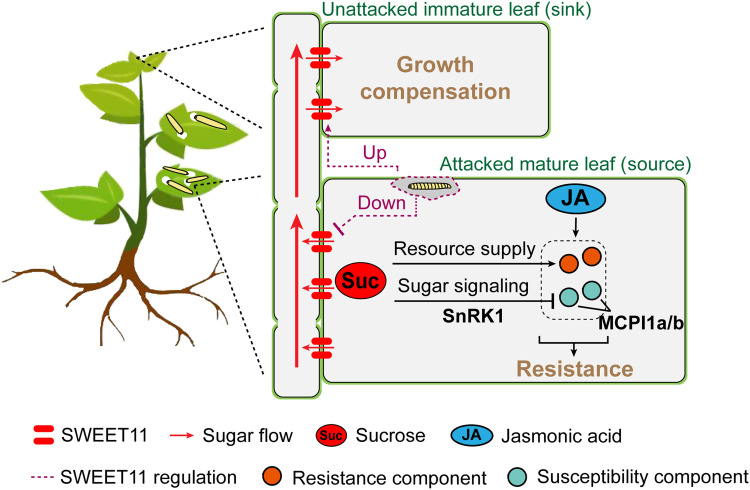
A model illustrates the proposed tissue-specific regulation of SWEET11, which mediates two distinct defense strategies against a specialist leafminer. Tissue-specific regulation of SWEET11 orchestrates two responses to herbivory in potato plants: tolerance and resistance. When mature leaves are attacked by *P. operculella* larvae, plants up-regulate SWEET11 in systemic immature leaves while rapidly repressing SWEET11 in attacked mature leaves. The differential regulation of SWEET11 leads to tissue-specific control of sugar transport. Increased SWEET11 expression in systemic immature leaves facilitates sugar import, directly promoting growth which can compensate for leaf loss caused by herbivore damage. Conversely, repressed SWEET11 in elicited mature leaves inhibits sugar export, rapidly elevating intracellular sugar levels that enhance herbivore resistance. The accumulation of sugar in attacked leaves serves dual roles. First, it may provide energy that supports the production of antiherbivore defense responses. Second, the rapid increase in intracellular sugar amplifies sugar signaling, which cooperates with JA signaling to promote previously unknown herbivore resistance traits. Specifically, herbivory-induced sugar signaling was shown to suppress JA-induced susceptibility components, MCPI1a and MCPI1b, through the sugar sensor SnRK1α (StubSNF1). This coordinated regulation of sugar transport in attacked mature leaves and unattacked systemic immature leaves ensures an effective balance of tolerance (growth compensation) and resistance, optimizing plant fitness in response to herbivory from a miner that mines mature leaves and is unable to move freely on plants to attack young leaves.

While much is known about the individual tolerance and resistance responses by which plants respond to herbivore attack (*3*, *42*), little is known about how plants integrate these two strategies at a whole-plant level. Here, we demonstrate that potato plants use a spatially segregated defense strategy against a specialist leafminer, inducing resistance in attacked leaves while promoting compensatory growth and tolerance in sink tissues. These responses are coordinated by a SWEET11-dependent carbohydrate reallocation that engages cross-talk between sugar and JA signaling pathways in herbivore-attacked leaves (Fig. 8).

Potato plants are known to exhibit significant tolerance to the attack of many insect species, and source-sink regulation is known to vary according to the particular tissues damaged. When attacked by *Tecia solanivora*, which exclusively feeds on tubers, unattacked tubers on attacked plants accumulate higher levels of starch and have elevated sink strength, while source leaves increase their sucrose synthesis and likely their export (*43*, *44*). Many potato cultivars can tolerate the *Leptinotarsa decemlineata* Say (*45*–*47*) and *Empoasca fabae* (*48*) damage to their leaves, and their tuber yields remain unaffected. In addition to potato, many herbaceous and woody plants also exhibit considerable tolerance to leafminer damage, primarily through aboveground vegetative compensation (*49*–*53*). In rice, attack by the striped stem borer (*Chilo suppressalis*) leads to increased tiller numbers when plants are grown under low fertilization rates (*54*), and attack by the western corn rootworm (*D. virgifera virgifera*) increases crown root production in maize (*18*). Future research may reveal if the SWEET family proteins are modulating carbohydrate reallocation patterns in these different plant–herbivorous insect systems. In addition, while we observed an overall yield compensation in tuber production in response to *P. operculella* attack to leaves, it remains to be seen if this compensation was a direct result of carbohydrate reallocation to tubers or an indirect effect of enhanced growth of immature leaves. In addition to mediating the aboveground source-sink relationships, SWEET11 is also involved in the carbohydrate allocation in stolons (*36*), while potatoes are stem tubers. Subsequent studies could clone stolon-specific promoters to spatially control tuberization (*55*), to specifically manipulate SWEET11 and investigate compensation responses in stolons.

While studies have demonstrated that SWEETs respond to biotic stresses primarily at the transcriptional level (*21*), their transcriptional regulators remain largely unknown. SWEETs are transcriptionally regulated in response to both pathogens (*56*–*59*) and herbivores (*60*), and these regulations are highly flexible depending on host plant species and insect feeding characteristics (e.g., mouthparts and feeding sites). This study and recent work (*60*) indicate that the transcriptional regulation of SWEETs in response to herbivore attack is largely independent of JA signaling. A recent study found a plant-specific transcription factor, DNA BINDING WITH ONE FINGER (DOF), to directly control the transcription of SWEETs, OsSWEET11 and OsSWEET14, in rice (*61*). Moreover, plant DOFs are also known to respond to abscisic acid (ABA) signaling (*62*–*65*). Since *P. operculella* damage can induce endogenous ABA levels in potato plants (*41*, *66*), we hypothesize that the ABA-DOF pathways transcriptionally regulate SWEETs in potato plants under herbivore attack. In addition to their transcriptional regulation, the activities of two SWEETs in *Arabidopsis*, SWEET11 and SWEET12, were found to be directly regulated by ABA signaling through SnRK2’s phosphorylation on their C terminus (*67*). It will be interesting to investigate how the tissue-specific transcription and function of SWEET are regulated by herbivore attack.

Sugars play different roles in resistance responses in insect attacked tissues. On one hand, sugars can directly function as energy sources and substrates for chemical defense compounds (*68*). On the other hand, sugars can act as signaling molecules that directly modulate local defense responses (*23*, *24*). While our study does not distinguish these two roles for the sugars differently allocated to the *P. operculella* attacked leaves, it identifies a sugar-mediated defense signaling. In this signaling, elevated sugars suppress transcripts, protein levels, and activity of SnRK1, which further destabilized MCPI1a/b and improves plant defense. Recently, sugar signaling has been found to regulate defense responses to plant pathogens: An HXK in *Arabidopsis* functions as the sugar sensor activating upstream immune responses, including pathogen-associated molecular pattern–triggered and effector-triggered immunity (*23*, *69*). Elucidating the downstream targets of SnRK1 may reveal the role of sugar signaling in herbivore resistance.

MCPI1 is a type of PR-6 (pathogenesis-related 6) protein with antimicrobial activity (*70*) that accumulates following pathogen infection (*71*) and is also known to increase a plant’s susceptibility to insect herbivores (*32*, *33*, *72*). During pathogen infection, mature MCPI1 proteins enter pathogen cells and inhibit their growth (*33*). Here, we show that potato MCPI1 is regulated by both JA and sugar signaling after herbivore attack and the two signaling systems have opposite effects on MCPI1 accumulations, with JA signaling increasing transcript accumulations and sugar signaling destabilizing its protein. As overexpression of this protein may disrupt flowering (*73*), a plant’s need to avoid overaccumulating MCPI1 proteins during a defense response may explain the evolution of this dual-control system. A previous study in tomato also found that the accumulation of this protein is well balanced in response to wounding (*38*). Moreover, this regulation may also help plants to minimize the resource costs of producing MCPI1 proteins.

The tradeoff between defense and growth, due to their high cost when photosynthetic outputs are limited, is commonly believed to be a principal determinant of fitness optimization when plants are attacked by insect herbivores (*74*, *75*). Here, we enrich this perspective with evidence that potato plants can balance investments in resistance and growth with the same sugar molecule in different tissues. As a result, potato plants achieve fitness through well-controlled whole-plant carbohydrate mobilization. SWEETs have been previously found to be involved in the defense in other plant species, although their regulation of carbohydrate mobilization and utilization can vary (*76*–*79*). In potato plants, the tissue-specific regulation of SWEETs by herbivore damage cues is likely a result of long-term coevolution with *P. operculella* (*30*, *31*). As mature and immature leaves both have their respective systems that coordinate with the function of these sugar transport channels, it is likely that specific herbivory signals engage the differences in these systems. Furthermore, coevolution may also have shaped the biological characteristics of *P. operculella* to promote the coordination of these two defense strategies in potato plants. As a leafminer, its low mobility may “prevent” its attack of new growth. Meanwhile, the damage of leafminers that have colonized plants may protect plants from further damage, since this damage elicits the release of a bouquet of plant volatile compounds that repels adults from further oviposition (*80*, *81*).

## MATERIALS AND METHODS

### Plant materials, growth conditions, and treatments

Potato (*Solanum tuberosum* L.) cultivar E-Potato-3 (cv. E3) was used as WT and for plant transformation and subsequent experiments. All transformants generated for this study, namely, SWEET11-KD, SWEET11-OE, StubSNF1-KD, StubSNF1-GFP-OE, MCPI1a-OE, and MCPI1b-OE (see the Supplementary Materials) and the JA biosynthesis–deficient (irAOC) plants previously reported (*41*) were constructed using cv. E3. All plants were tissue-cultured on Murashige-Skoog (MS) medium at 20° ± 1°C under a 16-hour light/8-hour dark cycle (100 μmol m^−2^ s^−1^, LED T5 21W). Three-week-old seedlings were transplanted into pots (Φ 9 cm) and grown in glasshouses (~22°C, light:dark = 16 hours:8 hours) for 28 days.

The following plant treatments were conducted: wounding plus water (W + W), herbivory elicitation or wounding plus oral secretions (W + OS), herbivore damage, and intensive herbivore damage. Herbivory elicitation (W + OS) treatments were performed using fabric tracing wheels and diluted *P. operculella* oral secretions. Briefly, mature leaves were mechanically wounded along the main vein using a fabric tracing wheel at 0.5-cm intervals; either pure water (W + W) or 1:5 diluted OS (W + OS) was applied to the wounds. Herbivory elicitation (W + OS) has been shown to efficiently and accurately elicit herbivory defense responses in potato plants (*66*). For herbivore damage, four newly hatched larvae were placed on three mature leaves (nodes 2, 3, and 4) and allowed to feed for one generation. For intensive herbivore damage, 30 fourth-instar larvae were placed on a single leaf and allowed to feed for 1 hour to induce defense responses (fig. S22).

### Vector constructs

All plasmids were constructed using the ClonExpress II One Step Cloning Kit (catalog no. C112; Vazyme, Nanjing, China). Coding sequence (CDS) was cloned from the *S. tuberosum* group Tuberosum RH 89-039-16 cDNA library. For stable gene overexpression in potato plants, the CDS of SWEET11, StubSNF1-eGFP, MCPI1a, and MCPI1b were cloned into pBI121 vector at the Xba I restriction site under the CaMV 35*S* promoter (p35*S*). For gene KDs, sense and antisense CDS fragments of SWEET11 (618 to 817 bp) and StubSNF1 (911 to 1191 bp) were cloned into pHELLSGATE8 vectors in inverted-repeat orientations to construct RNAi plasmids. For transient gene expression, the CDS of SWEET11-eGFP, StubSNF1-eGFP, MCPI1a-Myc, and MCPI1b-Myc were cloned into pCAMBIA1300s vector at the Kpn I restriction site under p35*S*. For complementation assays in yeast, the CDS of SWEET11 was cloned into pDR196 vector at the Eco RI restriction site under the phorbol 12-myristate 13-acetate (PMA) promoter. For Y2H assays, bait sequences were cloned into pGBKT7 vector at the Eco RI restriction site; prey sequences were cloned into the pGADT7 vector at the Eco RI restriction site. For BiFC, the CDS (with stop codon removed) of StubSNF1 was cloned into the pUC-SPYNE(173) vector at the Kpn I restriction site under p35S; the CDS (with stop codon removed of MCPI1a and MCPI1b) was cloned into the pUC-SPYCE(M) vector at the Kpn I restriction site under p35*S*. For recombinant protein expression and purification, the CDS of optimized StubSNF1 was cloned into pET28a vector at the Xho I restriction site with additional “GC” at 5′ end, and the CDS of optimized MCPI1a and MCPI1b were cloned into the pGEX-4T-1 vector at the Eco RI restriction site.

### Plant transformation

*A. tumefaciens*–mediated transformation was used to construct transgenic potato plants. E3 potato tubers were cleaned, peeled, and sterilized. Sterilized tubers were cut into 0.5 cm–by–0.5 cm pieces and incubated on 1/2 MS medium for 6 to 8 days until shoots emerged. Leaves were detached and incubated on precultivation medium [MS (4.43 g/liter), 3% sucrose, 0.2% casein hydrolysate, 2,4-D (1 mg/liter), and kinetin (0.5 mg/liter) (pH 5.8)] for 2 days. *A. tumefaciens* strain GV3101 harboring the recombinant transferred DNA (T-DNA) vector was cultured at 28°C in LB liquid medium with antibiotics until OD_600_ (optical density at 600 nm) reached 0.5. Bacterial cells were resuspended in an equal volume of LB liquid medium containing 50 μM acetosyringone. Explants were incubated in the *Agrobacterium* suspension for 5 min with gentle shaking. After inoculation, explants were dried on sterile filter paper and transferred to cocultivation medium [MS (4.43 g/liter), 3% sucrose, naphthaleneacetic acid (2 mg/liter), and 6-benzylaminopurine (6-BA; 2 mg/liter) (pH 5.8)] and incubated at 28°C for 2 days in the dark. Explants were then transferred to differentiation medium [MS (4.43 g/liter), 2% sucrose, 6-BA (0.5 mg/liter), indole-3-acetic acid (0.2 mg/liter), gibberellic acid (GA; 0.2 mg/liter), zeatin (2 mg/liter), cefotaxime (200 mg/liter), vancomycin (200 mg/liter), and kanamycin (15 mg/liter) (pH 5.8)] until shoots emerged, with medium refreshed every 2 weeks. Once shoots emerged, they were transferred to rooting medium [MS (4.43 g/liter), 3% sucrose, cefotaxime (200 mg/liter), vancomycin (200 mg/liter), and kanamycin (15 mg/liter) (pH 5.8)]. Transformed plants were confirmed by detecting the neomycin phosphotransferase II gene using qRT-PCR.

### Transient gene expression

*A. tumefaciens*–mediated transient expression was conducted in *N. benthamiana* leaves. *A. tumefaciens* strain GV3101, containing the recombinant T-DNA vector, was cultured at 28°C in LB liquid medium with antibiotics until the OD_600_ reached 0.5. The bacterial cells were then resuspended in an equal volume of infiltration buffer [10 mM MES, 10 mM MgCl_2_, and 200 μM acetosyringone (pH 5.6)] and incubated at room temperature for 3 hours. The bacterial suspension was infiltrated into the second fully expanded leaf using a syringe. After infiltration, the plant was kept in the dark for 12 hours before being returned to normal growth conditions. At 72 hours postinfiltration, the leaves were sampled or prepared for further assays. For coexpression of two proteins, bacterial cells were resuspended in infiltration buffer to OD_600_ = 1.0, mixed with equal volumes, and incubated. For MG132 treatment, as described previously (*82*), 20 μM MG132 or an equivalent dilution was infiltrated into the protein-expressed leaf 24 hours before sampling.

Polyethylene glycol (PEG)–mediated transfection was used for transient gene expression in *N*. *benthamiana* leaf protoplasts. True leaves from 2-week-old *N. benthamiana* plants were cut into 0.5-mm strips along the main vein. Plant cell walls were digested in an enzymatic digestion solution [20 mM MES, 1.5% cellulase R-10, 0.4% macerozyme R-10, 0.1% pectinase Y-23, 0.4 M mannitol, 20 mM KCl, 10 mM CaCl_2_, and 0.1% bovine serum albumin (pH 5.7)] for 4 hours in the dark. Protoplasts were isolated using a 100-μm cell strainer, and the vector was transfected into the protoplasts via the PEG-mediated method (see Supplementary Methods). The transfected protoplasts were incubated in the dark at room temperature for 16 hours before being used in assays.

### Measuring plant compensation (tolerance)

#### *Measuring compensation* (*tolerance*) *after herbivory*

Four newly hatched *P. operculella* larvae were inoculated onto three mature leaves (nodes 2, 3, and 4) of 4-week-old potato plants with similar growth (±2 cm in height). The leaf area of systemic immature leaves was measured using a portable leaf area meter (YMJ-A; HENGMEI, China) 3 and 9 days after herbivory. At 45 days postherbivory, the biomass (dry weight) of the aboveground parts and tubers was measured to assess plant tolerance.

#### Measuring compensation after W and OS elicitations

Three mature leaves (nodes 2, 3, and 4) of 4-week-old potato plants (with similar growth) were treated with W + W or herbivory elicitation (W + OS) every other day for a total of three times. The area of elicited mature leaves, systemic mature leaves, and systemic immature leaves was measured before each treatment and 2 days after the final treatment using a portable leaf area meter.

### *P. operculella* bioassays and plant damage measurement

#### Bioassays on plants

Four newly hatched *P. operculella* larvae were inoculated onto two mature leaves (nodes 1 and 2) of potato plants. The inoculated plants were kept under normal growth conditions. Larval biomass was recorded 12 days postinoculation, and leaf damage size was quantified using ImageJ software. To assess larval development time and survival rates, four newly hatched larvae were inoculated onto three mature leaves (nodes 1, 2, and 3). Fourteen days postinoculation, the infested leaves were enclosed in mesh bags to retain mature larvae. Larval development time (time to pupation), larval survival rate (pupation rate), and lifetime survival rate (adult eclosion rate) were recorded from days 15 to 30 postinoculation.

#### Bioassays on elicited leaves and systemic leaves

Three mature leaves (nodes 2, 3, and 4) of 4-week-old plants were treated with W + W or herbivory elicitation (W + OS). At 72 hours posttreatment, elicited leaves (node 2), systemic mature leaves (node 1), and systemic immature leaves (node −2) were detached and inoculated with four newly hatched larvae. The inoculated leaves were maintained under normal growth conditions, with water supplied through the petiole using wet cotton balls. Larval biomass was recorded 5 days after inoculation, and leaf damage size was quantified using ImageJ software.

#### Bioassays on sugar-supplied or MeJA-treated leaves

Mature leaves (nodes 1 and 2) were supplied with exogenous sugar or treated with MeJA before the bioassays. Four newly hatched larvae were inoculated onto leaves 18 hours after sugar supplementation or 48 hours after MeJA treatment. Inoculated leaves were kept under normal growth conditions, and water was supplied via the petiole using wet cotton balls. Larval biomass was recorded 7 days after inoculation.

### Exogenous sugar supplementation and MeJA treatment

#### Exogenous sugar supplementation

Leaves were supplemented with sugars using a stem feeding method as previously reported (*83*). Briefly, mature potato leaves (nodes 1 and 2) were excised at 3 cm from the petiole base and placed in a 2-ml sugar solution for 6 hours. The sugar solution was prepared using high-purity grade (≥99%) sugar (Sigma-Aldrich). After the sugar feeding period, the leaves were returned to normal growth conditions and continuously supplied with water via the petiole using wet cotton balls.

#### MeJA treatment

MeJA (392707, Sigma-Aldrich) was dissolved in lanolin at a concentration of 5 mg/ml. Approximately 0.03 ml of the lanolin paste (~150 μg of MeJA) was applied to the mature leaves at the petiole (*84*), 0.5 cm from the leaf base.

### ^13^C tracing assays

Mature leaves were ^13^C-labeled using a previously reported method with few modifications (*85*). Mature leaves were enclosed in 200-ml transparent chambers containing 8.5 mg of NaH^13^CO_3_ (372382, Sigma-Aldrich) in a reactor. To generate 100 μM ^13^CO_2_, 500 μl of 1 M H_2_SO_4_ was injected into the reactor. The injection port was sealed immediately after the injection. Leaves were exposed to ^13^CO_2_-rich air in glasshouse for 3 hours, while residual ^13^CO_2_ was absorbed by Ca(OH)_2_. Following ^13^C labeling, the mature leaves were immediately treated with W + W and herbivory elicitation (W + OS). At 12 hours after treatment, different tissue types were sampled, dried, and ground into a fine powder. Dried aliquots (0.5 to 1 mg) were transferred to tin capsules, and their δ^13^C signatures were analyzed using elemental analyzer–isotope ratio mass spectrometry (Isoprime 100, Elementar Analysensysteme).

### In situ RNA hybridization

#### Probe synthesis

Digoxigenin (DIG)–labeled SWEET11 mRNA probes were synthesized from a SWEET11 CDS fragment (69 to 468 nt) using the DIG RNA Labeling Kit (Promega). The reverse primer was designed with an attached T7 promoter core sequence, while the forward primer contained an SP6 promoter core sequence (see data S3).

#### Hybridization

In situ hybridization was performed according to a previously reported method (*86*) with modifications. Briefly, immature (node −2) and mature (node 2) leaves from potato plants were collected and fixed in 4% paraformaldehyde prepared in phosphate-buffered saline buffer overnight at 4°C. The samples were then sequentially dehydrated through an ethanol gradient, cleared through a dimethylbenzene series, and infiltrated with molten paraffin for 3 to 4 days. Following paraffin infiltration, the samples were embedded in paraffin and sectioned transversely into 7-μm slices. The sections were mounted on ribonuclease-free glass slides and dried at 42°C. Before hybridization, the tissue sections were deparaffinized with dimethylbenzene and rehydrated through a graded ethanol series. The tissues were then subjected to proteinase K treatment and prehybridized in hybridization buffer for 4 hours at 55°C. After 17 hours of hybridization at 55°C with DIG-labeled probes, the slides were washed and incubated with anti–DIG alkaline phosphatase conjugate (Roche) overnight at 4°C. Last, the hybridization signals were visualized using bromochloroindolyl phosphate–nitro blue tetrazolium substrate solution, and the stained tissues were examined under a light microscope. Hybridization with sense probe served as a negative control.

### RNA sequencing

Following the experimental design (fig. S2A), local and systemic tissues from herbivory elicitation (W + OS)–treated plants were sampled at three time points. Total RNA was extracted using TRIzol reagent (Thermo Fisher Scientific), and high-quality RNA samples were subjected to paired-end sequencing on the HiSeq NovaSeq 6000 platform (Illumina), with at least three biological replicates. Differential gene expression analysis was performed using the R package “DESeq2,” with |log_2_ fold change| > 1 and *P* adjusted < 0.05 (*87*). Results of RNA-seq are showed in data S2.

### Confocal observation

Seventy-two hours after *Agro* infiltration in *N*. *benthamiana* leaves or 16 hours after vector transfection into plant protoplasts, fluorescence signals were detected and imaged using a confocal laser scanning microscope (LSM 800; ZEISS, Germany). GFP or YFP fluorescence was observed at an excitation wavelength of 488 nm and emission wavelength of 509 nm. The 4′,6-diamidino-2-phenylindole signal was observed at an excitation wavelength of 353 nm and emission wavelength of 465 nm. Chloroplast signals were observed at an excitation wavelength of 280 nm and emission wavelength of 618 nm. The mCherry signal was observed at an excitation wavelength of 587 nm and emission wavelength of 610 nm.

### Functional analysis of SWEET11 in yeast

The yeast strain Susy7/ura3 (sugar transport deficient) was used to analyze sucrose transport function. pDR196-SWEET11 was transformed into Susy7/ura3 yeast cells using the PEG/LiAC protocol, and growth was monitored after a 3-day culture on synthetic defined (SD) maltose/-Ura medium or SD sucrose/-Ura medium. The empty vector was used as a negative control.

### Y2H assays

A cDNA library was constructed using total cDNA form herbivory-elicited potato leaf (6 hours after W + OS) on the pGADT7 plasmid, following “All-Direct” library construction protocol (Biogene Biotech, Shanghai, China). The cDNA library was transformed into the pGBKT7-StubSNF1–bearing yeast strain Y2HGold using the YeastMaker Yeast Transformation System 2 (catalog no. 630439; Clontech). Transformants were screened on TDO (triple dropout) medium [SD -Leu/-Trp/-His medium with 10 mM 3-AT (3-amino-1,2,4-triazole) to inhibit self-activation of histidine synthase 3], and positive clones were sequenced at the pGADT7 multiple cloning sites.

To further validate the interaction between StubSNF1 and MCPI1a/1b, the coding sequences of MCPI1a and MCPI1b were cloned from potato cDNA library. After putative signal peptides were removed (MCPI1a: 1 to 29 amino acids, MCPI1b: 1 to 17 amino acids), the remaining coding sequences were inserted into the pGADT7 vector, and Y2H assays were performed on triple dropout (TDO) medium. Yeast growth on the double dropout (DDO) medium was observed 3 days postinoculation to confirm the presence of both plasmids, and growth on the TDO medium was monitored 6 days postinoculation.

### Phytohormone measurements

Jasmonates were measured using a liquid chromatography–mass spectrometry–based protocol as previously described (*66*). Briefly, metabolites in ~100-mg aliquots of frozen sample powder were extracted with 80% methanol containing isotope-labeled internal standards (10 ng per sample; D6JA, D6JA-Ile). JA and JA-Ile were analyzed using ultraperformance liquid chromatography coupled to electrospray ionization quadrupol-time-of-flight mass spectrometer (UPLC-ESI-QTOF/MS), with multiple reaction monitoring mode used to monitor precursor fragmentations and quantify metabolites.

### Carbohydrate and Tre6P analysis

Leaf soluble sugars were assayed using a gas chromatography–mass spectrometry–based protocol as previously reported (*41*), and leaf starch was assayed using a hydrolysis method. Starch was acid-hydrolyzed to glucose, and glucose was measured using the anthrone method. Additional details are provided in Supplementary Methods.

Leaf Tre6P levels were quantified using a modified version of the reported UPLC-MS protocol (*88*). Briefly, metabolites (~100 mg of fresh weight) were extracted with 500 μl of ice-cold CHCl_3_/CH_3_OH (3:7, v/v), and water-soluble components were separated from the CHCl_3_ phase by adding water. The aqueous CH_3_OH extract was evaporated to dryness under vacuum at −58°C, redissolved in 200 μl of water, and filtered through Amicon Ultra centrifugal filters (UFC5003; Millipore) to remove high–molecular mass components. Last, Tre6P was analyzed by UPLC-ESI-QTOF/MS. Additional details are provided in Supplementary Methods.

### Expression and purification of recombinant proteins in *E. coli*

The plasmids pGEX4T-1-MCPI1a, pGEX4T-1-MCPI1b, and pET28a-StubSNF1 were transformed into *Escherichia coli* BL21 cells. For the production of GST-MCPI1a/1b, bacteria were cultured in 1 liter of LB medium at 37°C. When the OD_600_ reached 0.4 to 0.5, 1 ml of 1 M IPTG (isopropyl-β-d-1-thiogalactopyranoside; Sangon Biotech) was added to induce recombinant protein expression at 30°C for 3 hours. The bacterial cells were then ultrasonically disrupted, and the protein was purified using GST Sepharose 4FF (Sangon Biotech). For 6×His-StubSNF1, *E. coli* was grown in 100 ml of LB medium at 37°C. When the OD_600_ reached 0.4 to 0.5, 0.1 ml of 1 M IPTG was added to induce protein expression at 16°C for 12 hours. After ultrasonic disruption of the cells, the protein, present in the inclusion body, was purified using HisSep Ni-NTA Agarose Resin (Yeasen), following the manufacturer’s instructions.

### GST pulldown

Twenty microliters of GST beads (G10510, LABLEAD) was washed and equilibrated with 500 μl of tris-HCl buffer [25 mM tris-HCl (pH 7.6)] and then resuspended in 500 μl of the same buffer. Equal amounts (0.4 μg) of bait (GST-MCPI1a/1b) and prey (6×His-StubSNF1) proteins were mixed and incubated with the beads in 500 μl of tris-HCl buffer containing a protease inhibitor cocktail (P1005; Beyotime, Shanghai, China). The mixture was gently rotated at 4°C for 8 hours. After incubation, the supernatant was removed, and the beads were washed five times with 500 μl of tris-HCl buffer. Proteins were analyzed by Coomassie blue staining after protein mixing and by immunoblotting after GST pulldown.

### Coimmunoprecipitation

MCPI1a/1b-Myc was coexpressed with StubSNF1-GFP or GFP in leaf protoplasts. Leaf protoplasts were isolated using a combination of plant cell lysis buffer (P0043; Beyotime, Shanghai, China), protease inhibitor cocktail (P1005; Beyotime, Shanghai, China), and phosphatase inhibitor cocktail (P1081; Beyotime, Shanghai, China). Approximately 2 × 10^5^ leaf protoplasts were mixed with 200 μl of lysis buffer and gently shaken on ice for 45 min. Then, 20 μl of Myc-magnetic beads (LABLEAD) was added to the protein extract, which was rotary-incubated at 4°C for 12 hours. The beads were separated using a magnet and washed five times with 500 μl of cell lysis buffer before being resuspended in 30 μl of the same buffer. Both input and precipitated proteins were analyzed by immunoblotting.

### BiFC assay

The combinations of StubSNF1-nYFP and MCPI1a/1b-cYFP were coexpressed in *N. benthamiana* leaf protoplasts, and YFP fluorescence signals were observed using a confocal laser scanning microscope (LSM 800; ZEISS, Germany). The combination of StubSNF1-nYFP and cYFP was used as a negative control.

### Quantitative reverse transcription polymerase chain reaction

Gene transcript abundance was quantified using qRT-PCR based on SYBR Green chemistry. Total plant RNA was extracted using TRIzol reagent (Thermo Fisher Scientific), and cDNA was synthesized using the Evo M-MLV RT Kit (code no. AG11705; Accurate Biology). The 20-μl reaction contained 10 μl of 2× SYBR Green Taq HS Premix (code no. AG11701, Accurate Biology), 2 μl of cDNA, and 0.4 μM primers. Fluorescence curves were monitored using C1000 thermal cycler (Bio-Rad). Gene transcript abundance was calculated using EF3d as the reference gene. Primer sequences used for qRT-PCR are listed in data S3.

### Immunoblotting

Protein samples were heated at 95°C for 10 min after the addition of SDS–polyacrylamide gel electrophoresis (SDS-PAGE) loading buffer. Proteins were separated by SDS-PAGE and transferred to a 0.45-μm PVDF membrane (Millipore) for immunoblotting. After overnight incubation with primary antibody at 4°C, target proteins were visualized using horseradish peroxidase–conjugated secondary antibodies (Fdbio, Hangzhou, China). Immunoblot signals were detected with the ImageQuant LAS4000 and quantified using ImageJ. Primary and secondary antibodies used in this study are listed in data S3.

### Statistics

Statistical analyses were performed using SPSS 25.0 software (IBM). Levene’s and Shapiro-Wilk tests were used to assess homogeneity of variance and data normality, respectively. For comparisons between two groups, the Student’s *t* test (for normally distributed data; two-sided test) or the Mann-Whitney *U* test (for nonnormally distributed data; two-sided test) was applied. One-way analysis of variance (ANOVA) was used to compare differences among multiple groups, followed by Fisher’s least significant difference (LSD) post hoc tests. Datasets that did not meet the assumptions of Student’s *t* test and ANOVA were transformed before analysis (log transformation; square root transformation; reciprocal transformation; Box-Cox transformation). Two-way ANOVA was used to examine the interactive effects of two variables. The PCC was used to assess the correlation between two variables. Results of statistical analyses are displayed in data S1.

## Supplementary Materials

### The PDF file includes:

Supplementary Methods Figs. S1 to S29 Legends for data S1 to S4 References
