# Supplementary material for: Tissue-specific regulation of a sugar transporter mediates both resistance and tolerance responses to attack from a leafminer in potato
Source: Sci Adv. 2025 Oct 29;11(44):eadx5951. doi: 10.1126/sciadv.adx5951 (PMC12571087; doi:10.1126/sciadv.adx5951)
Supplement: Supplementary file 1 — Supplementary Methods Figs. S1 to S29 Legends for data S1 to S4 References [file sciadv.adx5951_sm.pdf]

Science Advances
AAAS

Supplementary Materials for

Tissue-specific regulation of a sugar transporter mediates both resistance and tolerance responses to attack from a leafminer in potato

Zhiyao Mao *et al.*

Corresponding author: Wenwu Zhou, wenwuzhou@zju.edu.cn; Ian T. Baldwin, baldwin@cemps.ac.cn

Sci. Adv. **11**, eadx5951 (2025)
DOI: 10.1126/sciadv.adx5951

The PDF file includes:

Supplementary Methods
Figs. S1 to S29
Legends for data S1 to S4
References

Other Supplementary Material for this manuscript includes the following:

Data S1 to S4

Supplementary Methods:

Phenotypic characterization of potato plants

Plants were tissue-cultured in MS medium for 3 weeks and transplanted into pots (Φ 9 cm) and grown in a glasshouse (~22 ℃, L:D = 16:8). At an early growth stage (28 days after transplanting), plant heights were recorded. At 75 days, aboveground biomass, root biomass and potted tuber yield was recorded. For SWEET11-KD plants, the chlorophyll contents in young, mature and old leaves were measured, as old leaves displayed a faster rate of yellowing. Chlorophyll was measured using spectrophotometric method. A fresh leaf (~0.2 g) was ground in 5 mL of extraction buffer (acetone: ethanol, 1:1). The mixture was transferred to test tube and extraction buffer was added to a final volume of 10 mL. After 3 h of extraction in the dark, extraction buffer was filtered and absorbance was measured. The absorbance of chlorophyll a was measured at 645 nm wavelength and the absorbance of chlorophyll b was measured at 663 nm wavelength.

Preparation of protoplasts and vector transfection of protoplasts

After separation, protoplasts were centrifuged at 100×g for 4 min, 4 °C. After the supernatant removed, protoplasts were gently resuspended in 10 mL W5 buffer (125 mM $CaCl_2$, 154 mM NaCl, 5 mM KCl, 5 mM glucose, 2 mM MES, pH 5.6) and centrifuged at 100×g for 4 min, 4 °C. Protoplasts were then gently resuspended in 5 mL W5 buffer and kept on an ice-bath for 30 min. After centrifugation, protoplasts were resuspended in MMG buffer (15 mM $MgCl_2$, 4 mM MES, 400 mM mannitol, pH 5.6) and the density was adjusted to 2×10^6/mL. A volume of 10 μL vector (~1g L^{-1}) was gently mixed with 100 μL protoplasts and 110 μL PEG (40 % PEG4000, 200 mM mannitol, 100 mM $CaCl_2$). Transfection was conducted at room temperature for 10 min and terminated by adding 500 μL W5 to the mixture. Mixture was centrifuged at 100×g for 1 min and resuspended in 500 μL W5. Finally, protoplasts were incubated at room temperature in the dark.

Semi-artificial diet (Semi-AD) and larval bioassays

Around 100 g plant leaves were detached, ground into powder in liquid N_2, freeze-dried and prepared for 100 g semi-AD. After freeze-drying, dried leaf powder, 2.5 g agar, 1g Wesson salt mixture, 0.1 g cholesterol, 0.25 g linoleic acid, 0.05g Tween-80, sugar and 75 g water were blended; pH was adjusted to 6.5 using 1 M NaOH. After autoclave sterilization, micronutrients (0.04 g inositol, 0.5 mg vitamin B1, 1 mg vitamin B2, 2 mg vitamin B3, 0.2 g vitamin B4, 2 mg vitamin B5, 0.5 mg vitamin B6, 0.5 mg vitamin B9, 0.004 mg vitamin B12, 0.4 g vitamin C, 0.04 mg vitamin H) were added. Finally, food preservatives (176 mg sorbic acid, 0.003 mg streptomycin, 0.003 mg ampicillin) were added and semi-AD was air-dried in a ventilator to a final weight of 100 g.

Semi-ADs were divided into groups of 10 g each, and placed on sterile 1% agar. Eight 2nd instar larvae were placed on the ADs and allowed to feed for a duration of 6 d. Initial body weight and final body weight were recorded. Weight gain was calculated by subtracting mean initial weight from final body weight.

Measuring soluble sugar and starch

Extraction. Leaf samples were ground in liquid N_2 and aliquots (~50 mg, fresh weight) were prepared for assays. Aliquots were extracted by adding 500 μL of extraction buffer (60 % methanol: 25 % chloroform: 15 % water, 10 ppm adonitol as internal standard) with vigorous shaking at room temperature for 10 min. Water-soluble components were extracted by adding 500 μL of deionized water with vigorous shaking at room temperature for 10 min. After 16, 000 g centrifuge at 4 °C for 10 min, aqueous-CH_3OH phase was transferred to a new tube and used for assaying soluble sugars. Pellets were washed twice using deionized water and used for assaying leaf starch.

Measuring soluble sugar. Aqueous-CH_3OH phase was freeze-dried overnight. After freeze-drying, 25 μL of hydroxylamine hydrochloride (100 mg/mL) was added to the dissolve powders, and the solution was maintained in a water-bath at 80 °C for 5 min. A volume of 25 μL acetic anhydride was added into the solution, which was retained at

room temperature for 5 min. Soluble sugars were then extracted by adding 200 μL of CH_3Cl with vigorous shaking, and washed three times using 500 μL of deionized water. Residual water was removed using sodium sulfate. Soluble sugars were then assayed using gas chromatography-mass spectrometry (GC-MS; 6890N, Agilent) with a HP-5ms capillary column (19091S-433, Agilent) with the following GC-MS parameters: operation time was 26.73 min; oven temperature started from 50 °C, linearly increased to 220 °C within 3 min and then linearly increased to 280 °C within 7 min; solvent delay was set at 9.50 min; SIM mode was used for ion detection; the other parameters were default. For ion detection, SIM group 1 (43.0, 115.0 and 145.0) was used to detect adonitol; SIM group 2 (43.0, 103.0 and 145.0) was used to detect glucose and fructose; SIM group 3 (43.0, 169.0 and 211.0) was used to detect sucrose.

Measuring starch. Pellet (see above) was pre-reacted using 300 μL of deionized water at 100 °C for 15 min. After cooling, 600 μL of 8 M HCl was added, and starch was hydrolyzed to glucose at 100 °C for 15 min. The mixture was centrifuged at 16, 000 g, 22 °C for 15 min. Glucose content of the supernatant was measured using anthrone colorimetry. Starch content was calculated by the following equation:

$$\text{Starch content (mg/g)} = \frac{g \times 1.11}{SW}$$

where g is hydrolysate mass and SW is sample fresh weight.

Measuring Tre6P

Tre6P extraction. Leaf Tre6P was extracted as previous reported with modifications (88). Tre6P in aliquots of leaf frozen powders (~100 mg, fresh weight) were extracted by adding 500 μL of ice-cold $CHCl_3$/CH_3OH (3:7, v/v). The mixture was vigorously shaken and incubated at -20 °C for 2 h with occasional mixing. Water-soluble components were extracted from the $CHCl_3$ phase by adding 500 μL of deionized water and shaking at 4 °C for 20 min. After 1,400 g centrifuge at 4 °C for 10 min, the aqueous-CH_3OH phase was transferred to a new tube and the $CHCl_3$ phase was re-extracted using 500 μL of deionized water as previously described. The second aqueous-CH_3OH

phase was combined with the first and freeze-dried using a freeze vacuum dryer at -58 °C. The dried extract was redissolved in 200 μL of water and filtered through an Amicon Ultra centrifugal filter devices (Millipore) to remove high molecular-mass components by centrifuging at 3,300 g, 4 °C for 30 min. Samples were finally filtered through a 45 μM membrane filter and stored at -20 °C until LC-MS.

Tre6P analysis. Tre6P was assayed using UPLC (ACQUITY UPLC I-Class PLUS System, WATERS) with ACQUITY UPLC CSH C18 Column (186005297, WATERS), coupled to ESI and QTOF MS system (X500R, AB SCIEX). Injection volume is 10 μL. Mobile phase was the mixture of solvent A [Milli-Q water, 0.5 mM methylphosphonic acid, 0.001% (v/v) piperidine] and solvent B (acetonitrile), and column was equilibrated using 30 % solvent A. This mobile phase has been demonstrated to significantly increase intensity of Tre6P chromatographic peaks (89). A gradient elution at a flow rate of 0.3 mL/min and a column temperature of 40 °C was used for a total run time of 20 min: 0 – 15 min, 30 – 70 % A; 15 – 18 min, 70 % A; 18 – 20 min, 70 – 30 % A. System was re-equilibrated with 30 % solvent A. Metabolite was assayed using IDA mode with ESI negative ionization. For TOF MS, mass range: 50 – 1300 *m/z*; collision energy: 10 eV. For TOF MS/MS, mass range: 50 – 1300 *m/z*; collision energy: 42 eV; CE spread: ±5 eV. The other parameters were set by default. Tre6P chromatogram was selected by extracting ion chromatogram of $C_{12}H_{23}O_{14}P$ $[M-H]^-$; Tre6P identity was confirmed by fragment ions m/z 97 and m/z 79; the abundance of Tre6P was quantified using peak area.

Tre6P calibration curve and recovery assay. Stock solution of Tre6P (1 mM) was prepared in ultra-pure water using trehalose-6-phosphate dipotassium salt (Sigma-Aldrich) and was stored at -20 °C. A calibration curve containing 7 different Tre6P concentrations (0.01, 0.05, 0.1, 0.5, 1, 5, 10 μM) was prepared using Tre6P stock solution. To calculate Tre6P recovery rate from plant samples, 1 μL of Tre6P stock solution was added to an aliquot of frozen leaf powders and extracted as previously

described; in parallel, 1 μL of Tre6P stock solution was added to an empty tube and extracted as a control. Finally, Tre6P recovery rate was calculated and used to calculate Tre6P contents of plant samples.

Protein multi-alignments and phylogenetic analysis

Protein sequence acquisition. Ortholog candidates of AKIN10 were retrieved from the TAIR database (https://www.arabidopsis.org/). Potato MCPI proteins were identified using HMMER software: potato protein database was searched using hidden Markov model of PF02977 domain (Pfam database). Plant homologs of potato MCPI1 were identified by BLAST against the Uniprot protein database with the protein sequence of the potato carboxypeptidase inhibitor (Q9SBH8).

Phylogenetic analysis. Sequences were aligned using ClustalW with default settings. Conserved functional regions were selected for subsequent phylogenetic analysis. Phylogenetic trees were constructed using the Maximum Likelihood (ML) method, implemented in MEGA 11, with the Jones-Taylor-Thornton (JTT) model. Branch support was evaluated with 1000 bootstrap replicates, and all other parameters were set to default.

Measuring the inhibitory effects of leaf extracts on bovine carboxypeptidase A (CPA)

CPA reaction. Aliquots (~100 mg; fresh weight) of frozen sample powders were extracted by 150 μL of protein extraction buffer [0.1 M Tris-HCl, pH 7.6; 0.45 M NaCl, 0.2 % (w/w) Phenylthiourea, 5 % (w/w) PVPP]. Protein concentrations of leaf extracts were quantified using the Bradford method. Leaf extract (50 μg or 100 μg) was mixed with 50 μL of 10 μg/mL CPA solution, and Tris-HCl buffer (0.1 M Tris-HCl, pH 7.6) was added to bring total volume to 100 μL. The mixture was incubated on ice for 30 min. After incubation, CPA solution was mixed with an equal volume of substrate solution (2 mM hippuryl-L-phenylalanin in Tris-HCl buffer) and reacted at 30 °C for 45 min. The reaction was terminated by adding 100 μL of 7.5 M guanidine

hydrochloride.

Product (hippuric acid) assays. The reaction solution was freeze-dried, and products were redissolved in 500 μL of methanol. The product solution was filtered through a 45 μM membrane and prepared for LC-MS analysis. Hippuric acid was assayed using UPLC (ACQUITY UPLC I-Class PLUS System, WATERS) with ACQUITY UPLC CSH C18 Column (186005297, WATERS), coupled to ESI and QTOF MS system (X500R, AB SCIEX). Injection volume is 10 μL. Mobile phase was the mixture of solvent A [Milli-Q water containing 0.025 % (v/v) ammonium acetate] and solvent B [acetonitrile containing 0.025 % (v/v) ammonium acetate], and the column was equilibrated using 70 % solvent A. A gradient elution at a flow rate of 0.3 mL/min and a column temperature of 40 °C was used for a total run time of 12 min: 0 – 10 min, 70 – 30 % A; 10 – 12 min, 30 – 70 % A. System was re-equilibrated with 70 % solvent A. Metabolite was assayed using MRM mode with ESI negative ionization. For TOF MS, mass range: 50 – 1300 *m/z*; collision energy: 10 eV. For TOF MS/MS, mass range: 50 – 1300 *m/z*; collision energy: 36 eV; CE spread: ±5 eV. The fragmentation of hippuric acid was monitored and quantified based on the fragmentation from precursor to a fragment ion (m/z 178 – m/z 134).

Protein cell-free degradation assay

Protein cell-free degradation assays were performed as previously reported (90). Leaf soluble proteins were extracted by extraction buffer (25 mM Tris-HCl pH 7.5; 10 mM NaCl, 10 mM $MgCl_2$, 4 mM PMSF, 5 mM DTT, 10 mM ATP). The protein concentrations were quantified using the Bradford method and adjusted to 2.5 mg/mL. An amount of 0.1 μg target protein (GST, GST-MCPI1a, GST-MCPI1b) was added to 200 μL protein extract and incubated for 0, 1 or 3 h at 30 °C. Remaining proteins were detected and quantified using immunoblotting and Image J software.

Larval bioassays on detached leaves after intensive herbivory

Leaves at nodal positions 2, 3, 4 of 28-d plants were treated by intensive herbivore damage (30 4th *P. operculella* larvae feeding for 1 h). At 72 h after treatment, attacked mature leaves (node 2), systemic mature leaves (node 1), and systemic immature leaves (node -2) were detached. Larval bioassays were performed on these detached leaves as described above.

Synthesizing antibodies

Recombinant protein 6×His-StubSNF1 was directly used as an immunogen for synthesizing rabbit poly-antibody. After cleavage of the GST-tag of GST-MCPI1a/b using thrombin, the GST tag was removed by GST-affinity beads and thrombin was removed by filtering through HiTrap Benzamidine FF column (Cytiva). After being concentrated and cleaned by filtering through Amicon Ultra Filter Device (10 kDa MWCO), recombinant proteins MCPI1a and MCPI1b were used as immunogens for synthesizing rabbit poly-antibodies. Rabbit antibodies were synthesized and purified commercially (HUABIO, China). Two independent antibodies to an immunogen were independently synthesized in two rabbits. After purification, ELISA and immunoblotting were used to verify binding capability of antibody to its immunogen.

ELISA (enzyme-linked immunosorbent assay)

After diluting immunogen to 1 μg/mL using ELISA carbonate coating buffer (Thermo Fisher Scientific), 50 μL of immunogen was added into ELISA polystyrene microplate (Corning) and incubated at 4 °C overnight. After washing each well using 180 μL of 1×PBST buffer, 150 μL of 1% BSA was added into each well and the microplate was incubated at 37 °C for 1 h for blocking. After washing each well using 180 μL of 1×PBST buffer, 50 μL of diluted antibody solution was added into each well and the microplate was incubated at 37 °C for 30 min. After washing each well using 150 μL of 1×PBST buffer for 3 times, 50 μL of 1:10000 diluted secondary antibody (HRP Goat Anti-Rabbit IgG) was added into each well and microplate was incubated at 37 °C for

45 min. After washing each well using 150 μL of 1×PBST buffer for 3 times, 50 μL of TMB Chromogen Solution (Beyotime) was added into each well and microplate was incubated at 37 °C for 5 min. After the TMB reaction, 50 μL of 1 M H_2SO_4 was added to terminate the reaction. Absorbance at 450 nm was recorded using a microplate reader (Model 680, Bio-Rad).

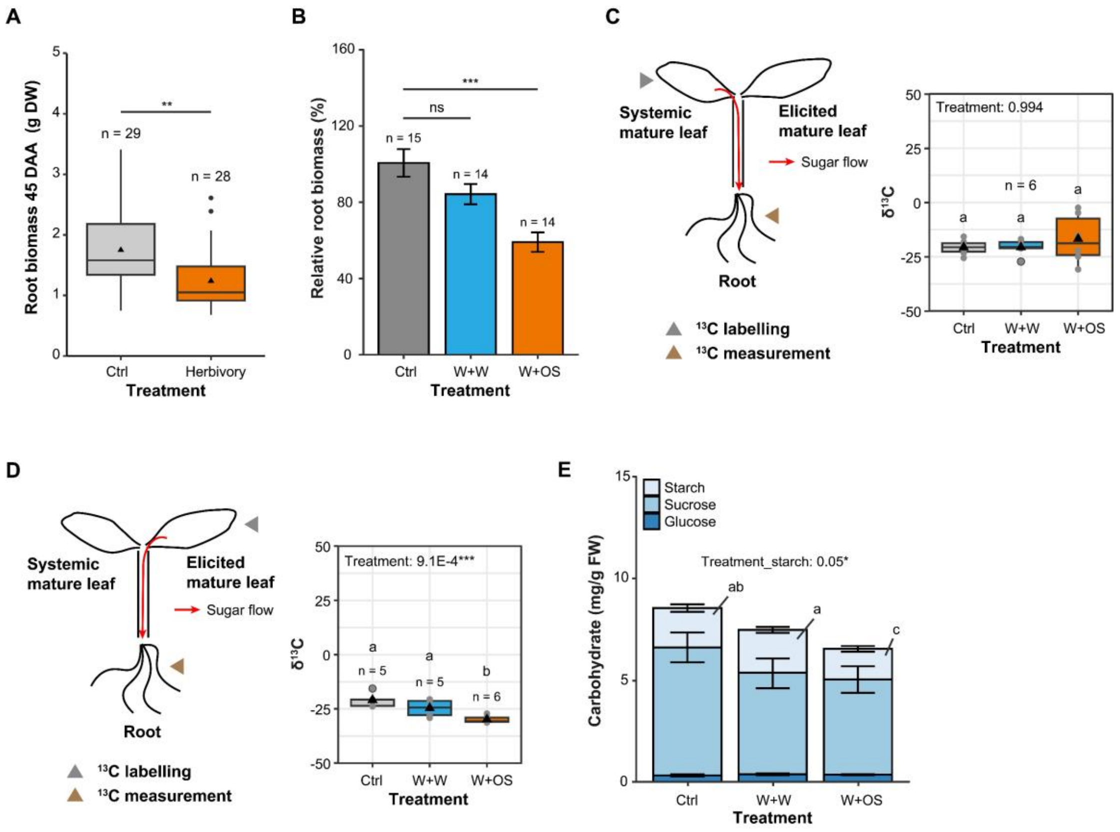

Fig. S1. *P. operculella* leaf herbivory leads to decreased sugar allocation to roots and reduces root growth.

(A) *P. operculella* herbivory (leaves at nodes 2, 3, 4 each attacked by 4 larvae) reduced root biomass 45 d after start of herbivory (boxplot, n = 28 – 29).

(B) Herbivory elicitation (W+OS) of mature leaves (at nodes 2, 3, 4) reduced root biomass. Relative root biomass was measured at 6 d after 1st treatments (DW; mean ± SEM, n = 14 – 15).

(C – D) Herbivory elicitation (W+OS) of mature leaves decreased sugar allocations to roots. Herbivory elicitation (W+OS) of mature leaves (at nodes 2, 3, 4) decreased elicited mature leaf (node 2)-derived root ^{13}C levels 12 h after elicitation (**D**; boxplot, n = 5 – 6), while it did not affect systemic mature leaf (at node 1)-derived root ^{13}C levels (**C**; boxplot, n = 6).

(E) Herbivory elicitation (W+OS) of mature leaves (at nodes 2, 3, 4) decreased root carbohydrate contents 12 h after elicitation (mean ± SEM, n = 5).

In boxplots, triangles refer to mean value; black dots refer to outliers; grey points refer to data points. Asterisks indicate significant differences between treatment and control

(Student's t test or Mann-Whitney U test, $*p < 0.05$, $**p < 0.01$, $***p < 0.001$). Different letters indicate significant differences among treatments (LSD *post hoc* multiple comparisons following one-way ANOVA, $*p < 0.05$, $**p < 0.01$, $***p < 0.001$).

DW, dry weight.

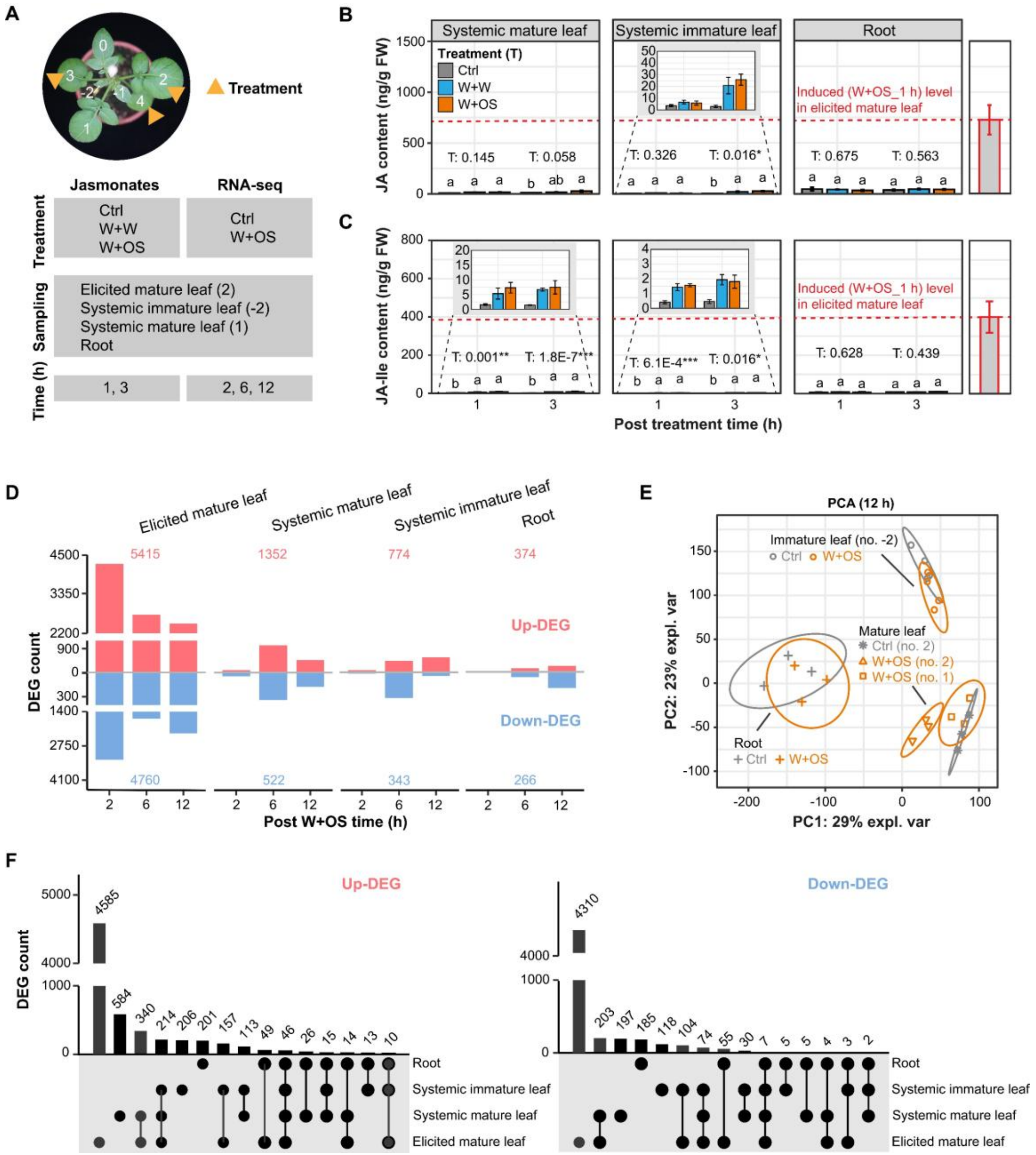

Fig. S2. Experimental design and analysis of jasmonate levels and transcriptomes in elicited and systemic tissues following W + OS elicitations of mature potato leaves.

(A) Experimental design of the analysis of local and systemic jasmonate levels and transcriptomes following W+OS elicitation of mature leaves. Potato plants were treated by herbivory elicitation (W+OS) of mature source leaves growing at nodes 2, 3, 4. Four types of tissues (elicited mature leaf, systemic immature leaf, systemic mature leaf and root) were sampled at different times after treatment. These samples were collected for jasmonate analysis or RNA-Seq. Systemic mature leaf and elicited mature leaf share a common control group (leaf node 2) because leaves at nodes 1 and 2 have similarly just

completing the sink-source transition.

(B) W+W and herbivory elicitation (W+OS) of mature leaves (at nodes 2, 3, 4) induced low levels of jasmonic acid (JA) in systemic mature leaves (node 1), systemic immature leaves (node -2) and roots (±SEM, n = 5).

(C) W+W and herbivory elicitation (W+OS) of mature leaves (nodes 2, 3, 4) induced low levels of jasmonic acid-isoleucine (JA-Ile) in systemic mature leaves (node 1), systemic immature leaves (node -2) and roots (± SEM, n = 5).

(D) DEG analysis of local and systemic transcriptomes. The total (three time points) number of DEGs is displayed above the bar.

(E) PCA reveals that sampled tissues are distinct with significantly different transcriptomic profiles. Ellipse refer to 95% confidence intervals.

(F) Set analyses of DEGs revealed that herbivory elicitation (W+OS) induced both shared and tissue-specific transcriptional responses. Analyses are visualized using UpSet plot.

Different letters indicate significant differences between treatments (LSD *post hoc* multiple comparisons following one-way ANOVA, *$p < 0.05$, **$p < 0.01$, ***$p < 0.001$).

RNA-seq, RNA-sequencing; DEG, differentially expressed gene; PCA, Principal Component Analysis.

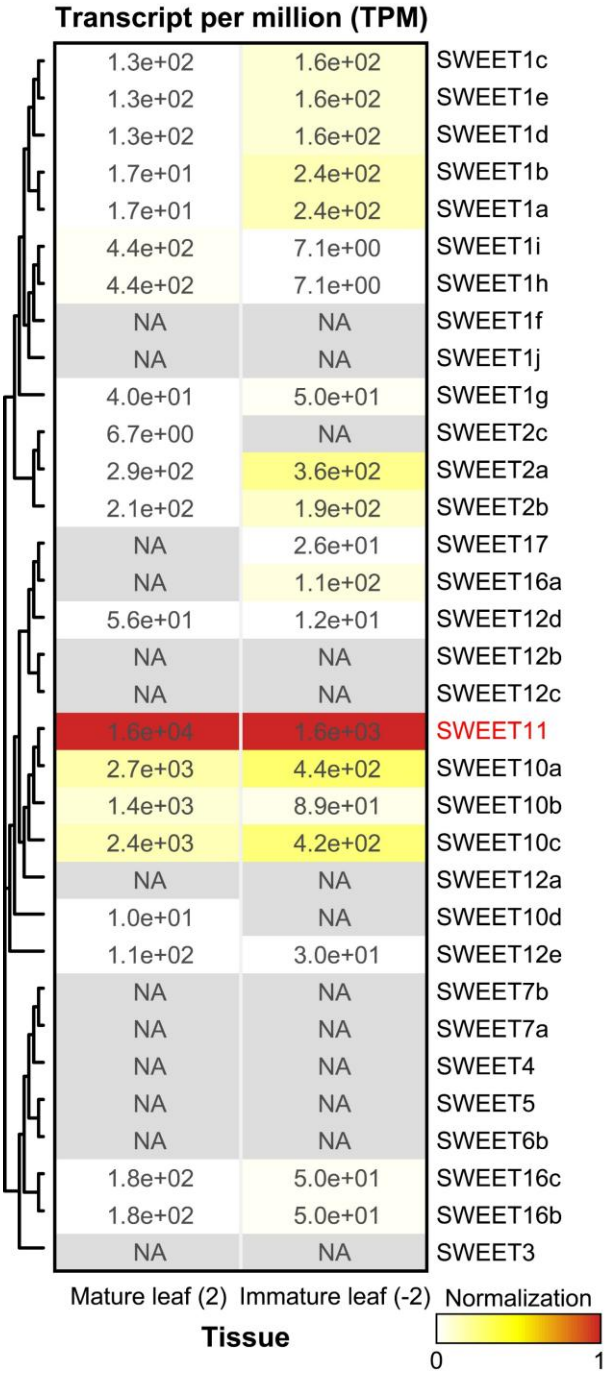

Fig. S3. Expression pattern of SWEET-gene family in mature and immature leaves. Analyzing transcript abundances (transcript per million) of 33 SWEET genes (91) in mature potato leaf (at node 2) and immature leaf (at node -2) using RNA-seq data. Transcript abundances in each tissue were normalized to a 0 – 1 range and visualized using a heatmap. NA, not available.

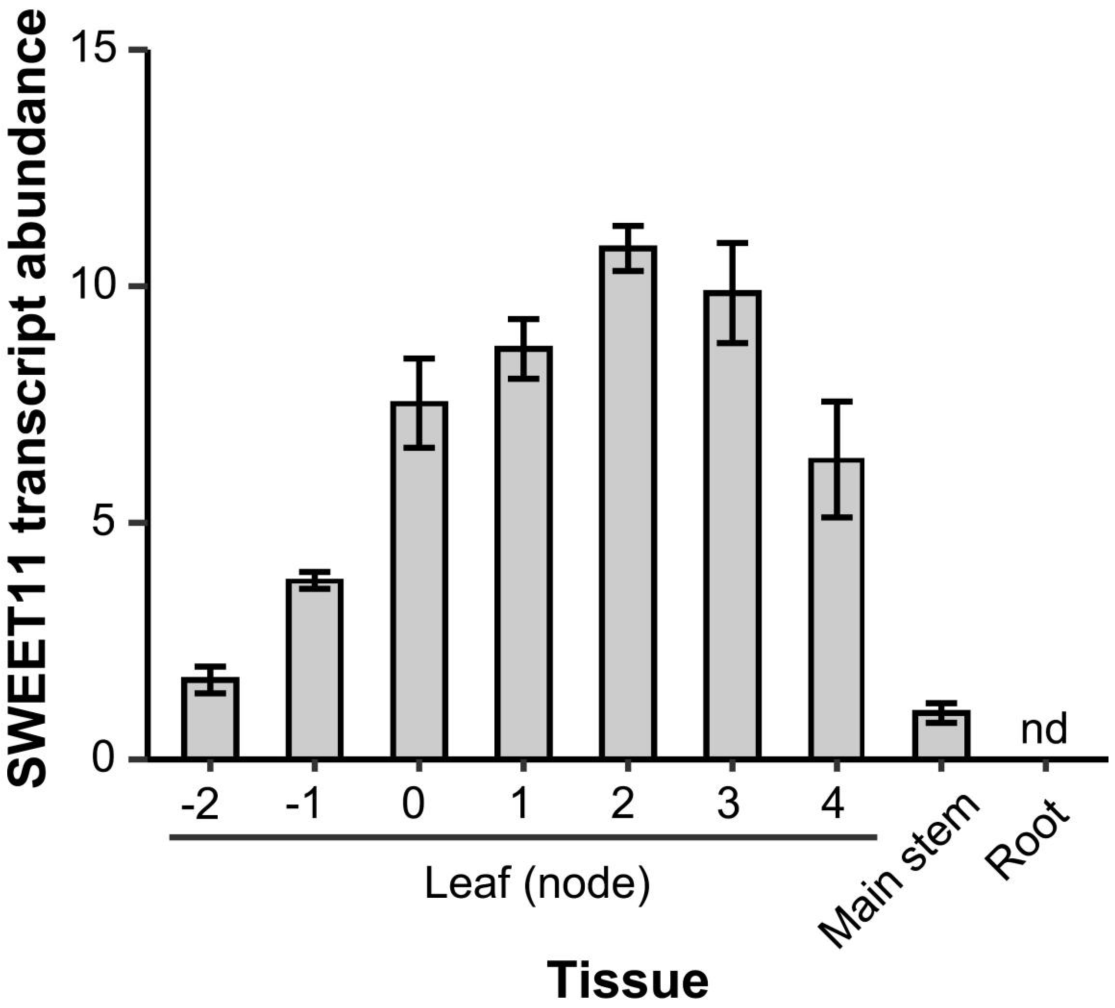

Fig. S4. Expression pattern of SWEET11 at the tissue level.

Transcript abundances of SWEET11 were quantified in different tissues of potato plants using qRT-PCR (mean ± SEM, n = 3; nd, not detected).

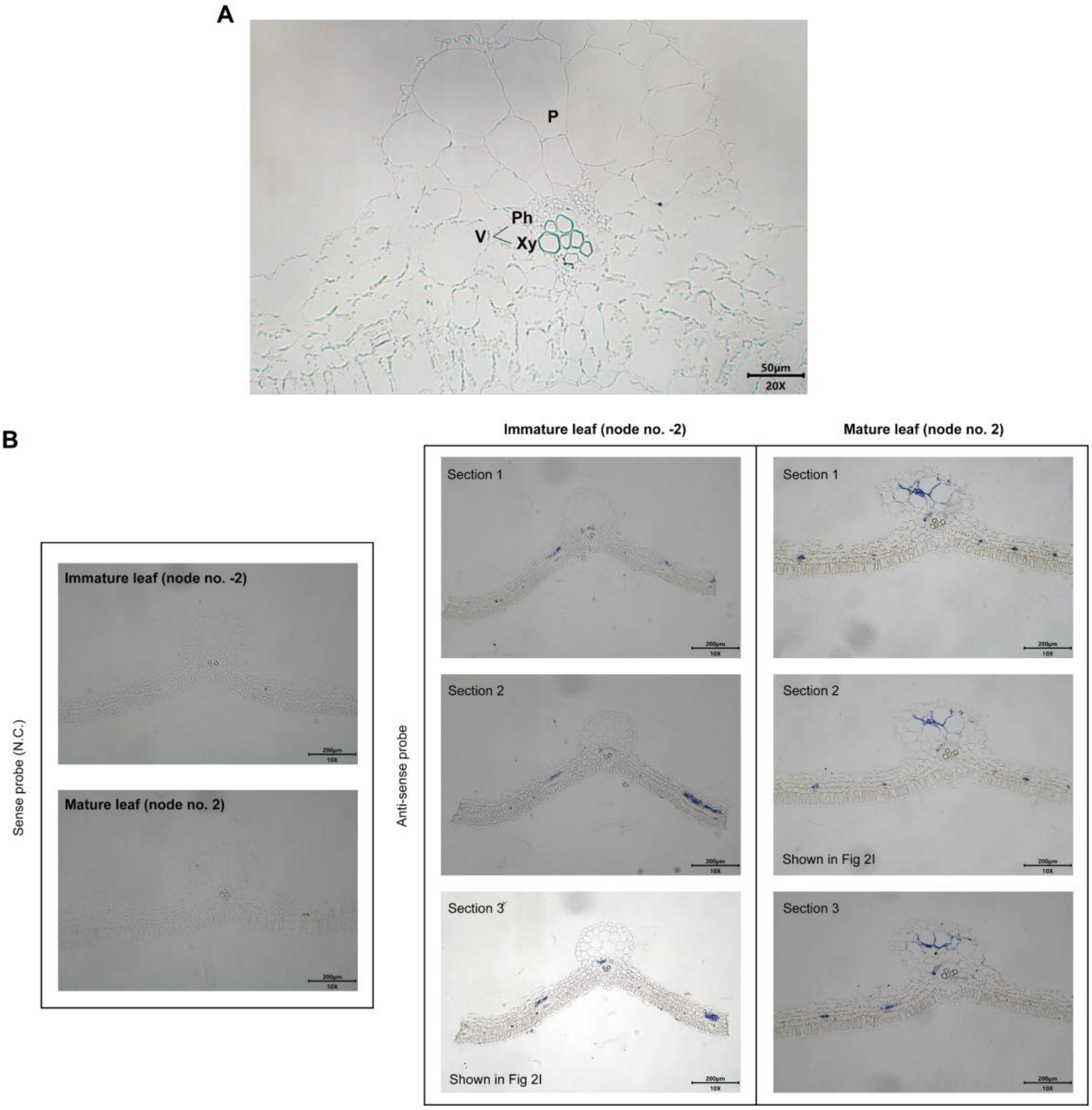

Fig. S5. Results of SWEET11 mRNA *in situ* hybridization in immature (node -2) and mature (node 2) leaves.

(A) Midrib cross section of a mature potato leaf. P, parenchyma; V, vasculature; Ph; phloem; Xy, xylem.

(B) Serial cross sections of the same leaf are labeled as serial numbers. Hybridization with sense-probe is used as negative control (N.C.).

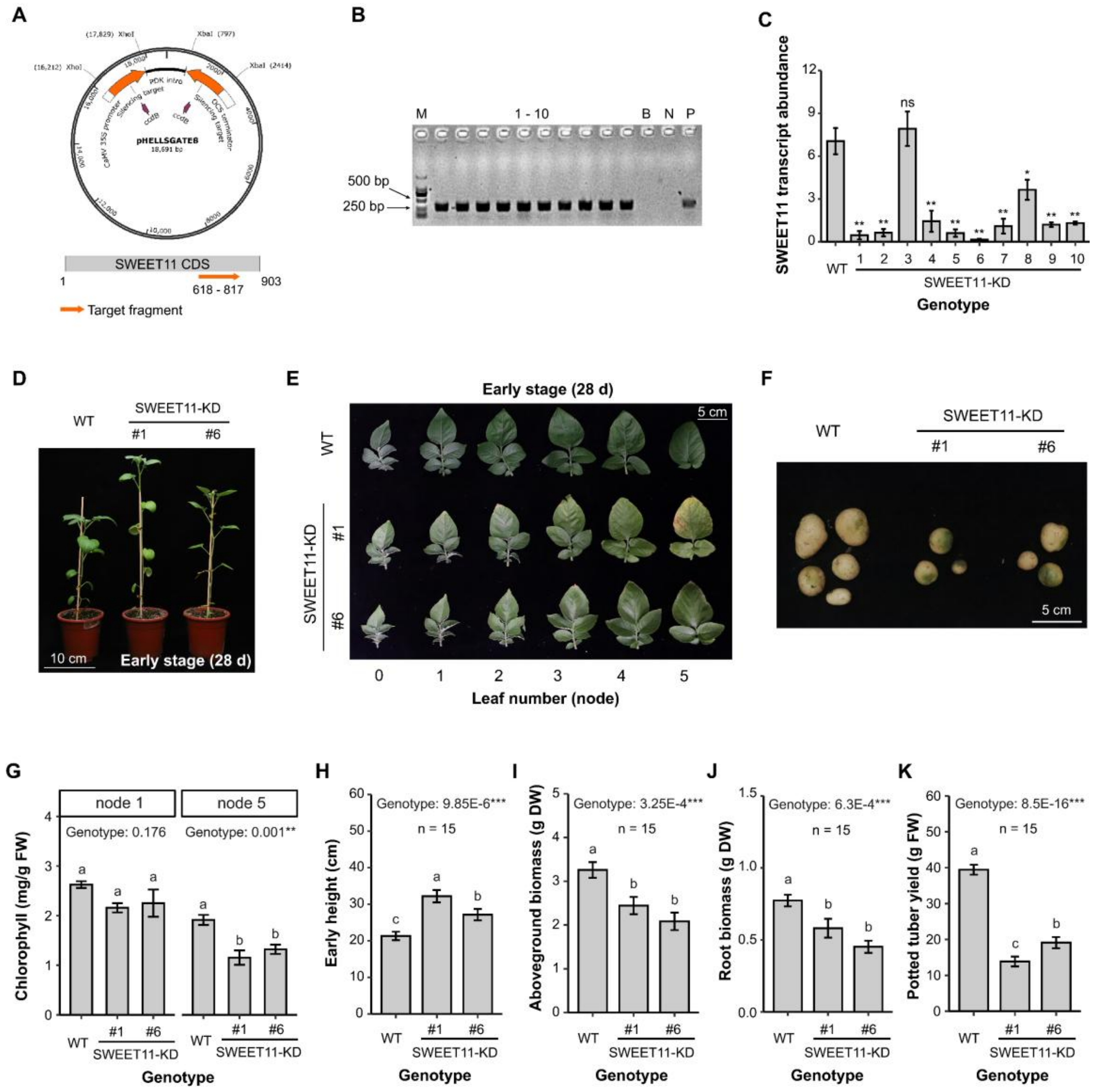

Fig. S6. Construction and phenotypic characterization of SWEET11-KD plants.

(A – C) Construction of SWEET11-KD plants.

(A) Design of inverted-repeat vector for knocking down SWEET11 by RNAi. A fragment of SWEET11 CDS (618 – 817) was cloned as the RNAi target. Sense target and antisense target were cloned into pHELLSGATE8 as shown. Plasmid harboring target was transformed into plants using *Agrobacterium tumefaciens*-mediated method.

(B) Detection of transformed plants using PCR. Screening marker NPTII gene was detected after transformation. M: marker; B: blank; N: negative control; P: positive control.

(C) Quantification of SWEET11 silencing efficiency (mean ± SEM, n = 3 – 5). The silencing efficiency of SWEET11 was assessed by quantifying its relative expression in mature leaves (node 2) of 28-d plants.

Asterisks indicate significant difference between KD and WT plants (Student's t test, *$p < 0.05$, **$p < 0.01$, ***$p < 0.001$; ns, not significant).

(D – J) Phenotypic characterization of SWEET11-KD plants.

(D) Representative plants at early growth-stage (28 d after transplanting).

(E) Leaf phenotypes at early growth-stage (leaf nodes 0 – 5).

(F) Tubers after harvesting (75 d after transplanting).

(G) SWEET11 knockdown decreased chlorophyll (chlorophyll a and b) contents in old leaves (mean ± SEM, n = 5).

(H) SWEET11 knockdown resulted in an increase in plant height 28 d after transplanting (mean ± SEM, n = 15).

(I) SWEET11 knockdown reduced aboveground biomass 75 d after transplanting (DW; mean ± SEM, n = 15).

(J) SWEET11 knockdown reduced root biomass 75 d after transplanting (DW; mean ± SEM, n = 15).

(K) SWEET11 knockdown reduced potted tuber yield 75 d after transplanting (FW; mean ± SEM, n = 15).

Different letters indicate significant difference between genotypes (LSD *post hoc* multiple comparisons following one-way ANOVA, *$p < 0.05$, **$p < 0.01$, ***$p < 0.001$).

KD, knockdown; RNAi, RNA interference; CDS, coding sequence. NPTII, neomycin phosphotransferase II; DW, dry weight; FW, fresh weight.

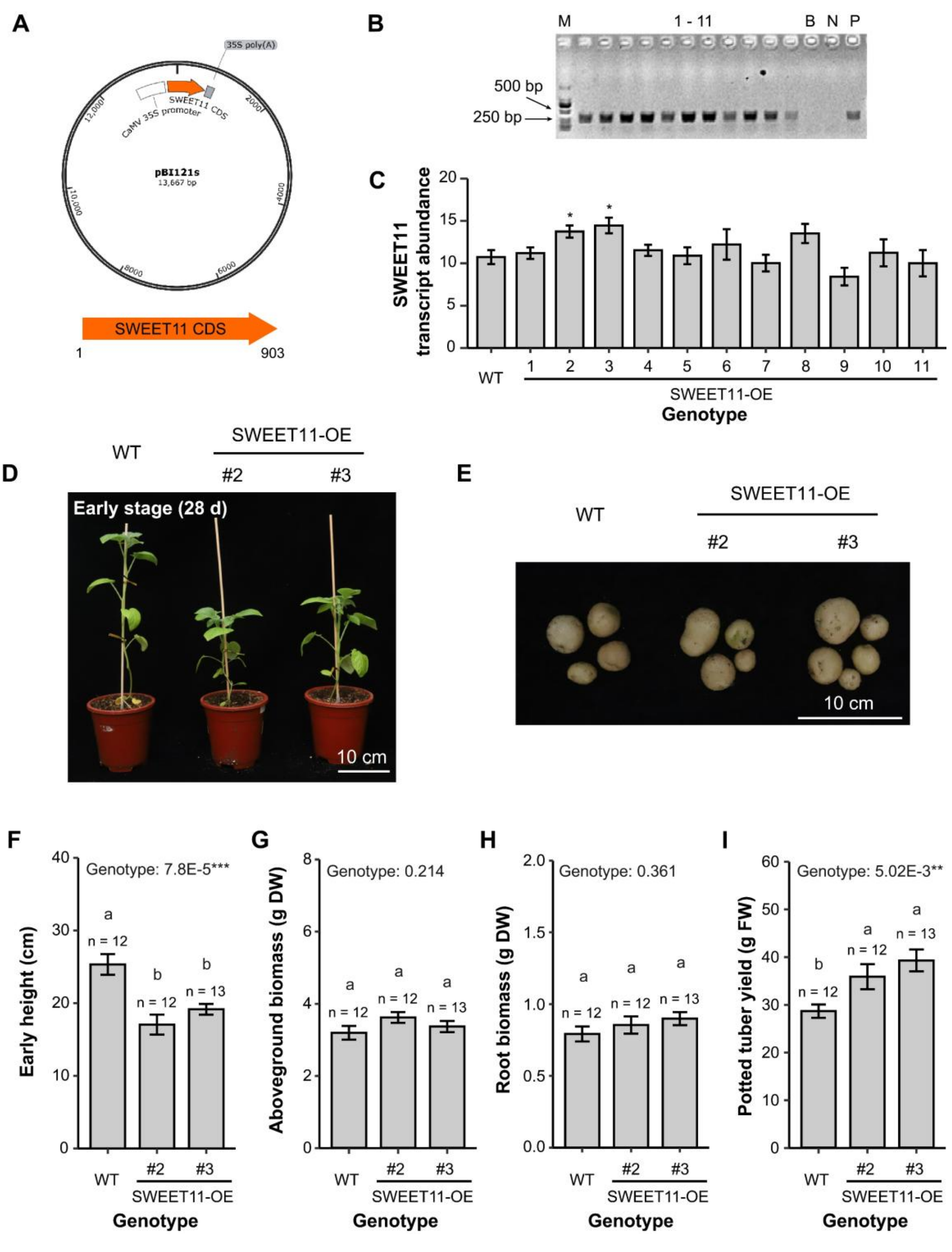

Fig. S7. Construction and phenotypic characterization of SWEET11-OE plants.

(A – C) Construction of SWEET11-OE plants.

(A) Design of SWEET11 overexpression transformation construct. SWEET11 CDS was cloned into the multiple cloning site of pBI121s as shown. Plasmid was transformed into potato plants by an *Agrobacterium tumefaciens*-mediated method.

(B) Detection of transformed plants using PCR. Screening marker NPTII gene was

detected after transformation. M: marker; B: blank; N: negative control; P: positive control.

(C) Quantification of SWEET11 overexpression efficiency (mean ± SEM, n = 3). The overexpression efficiency of SWEET11 was assessed by quantifying its relative expression in mature leaves (node 2) of 28-d plants.

Asterisks indicate significant difference between OE and WT plants (Student's t test, $*p < 0.05$, $**p < 0.01$, $***p < 0.001$; ns, not significant).

(D – I) Phenotypic characterization of SWEET11-OE plants.

(D) Representative plants at early growth-stage (28 d after transplanting).

(E) Tubers after harvesting (75 d after transplanting).

(F) SWEET11 overexpression resulted in a decrease in plant height 28 d after transplanting (mean ± SEM, n = 12 – 13).

(G) SWEET11 overexpression did not affect aboveground biomass 75 d after transplanting (DW; mean ± SEM, n = 12 – 13).

(H) SWEET11 overexpression did not affect root biomass 75 d after transplanting (DW; mean ± SEM, n = 12 – 13).

(I) SWEET11 overexpression resulted in an increase in potted tuber yield 75 d after transplanting (FW; mean ± SEM, n = 12 – 13).

Different letters indicate significant differences between genotypes (LSD *post hoc* multiple comparisons following one-way ANOVA, $*p < 0.05$, $**p < 0.01$, $***p < 0.001$).

OE, overexpression; DW, dry weight; FW, fresh weight.

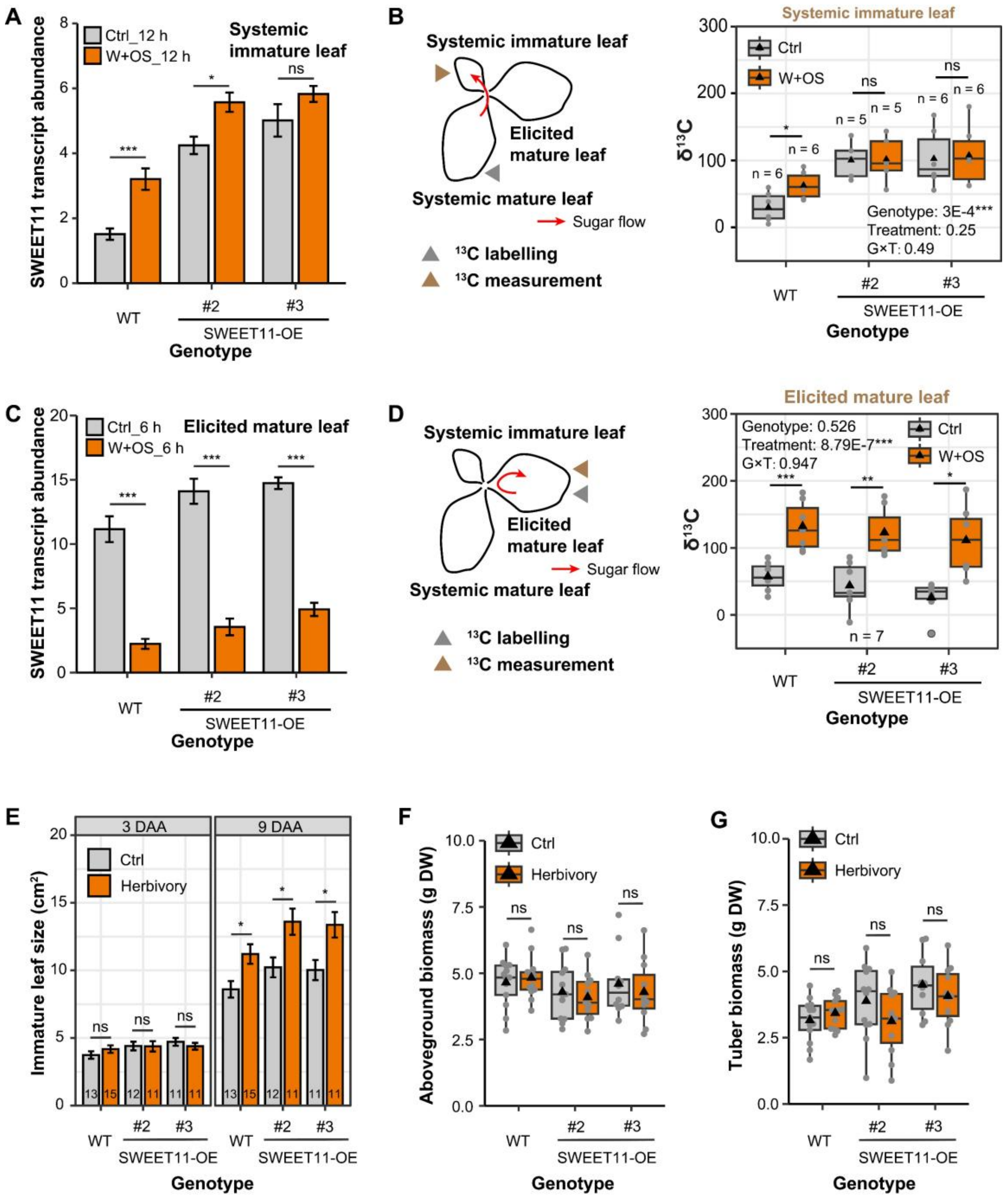

Fig. S8. The effects of SWEET11 overexpression on herbivory-induced sugar transport and herbivore tolerance.

(A) SWEET11 overexpression led to a constitutive increase in SWEET11 transcript abundance in immature leaves (node -2) (mean ± SEM, n = 4). SWEET11 transcriptional 12-h responses in systemic immature leaves following herbivory elicitation of mature leaves (nodes 2, 3, 4) were attenuated in SWEET11-OE plants.

(B) SWEET11 overexpression resulted in a constitutive increase in sugar import into immature leaves (node -2). Consequently, herbivory elicitation (W+OS) of mature

leaves (nodes 2, 3, 4) did not significantly enhance it within 12 h (boxplot, n = 5 – 6).

(C) SWEET11 overexpression did not affect the SWEET11 transcriptional response in elicited mature leaf (node 2) following herbivory elicitation (W+OS) (mean ± SEM, n = 4).

(D) SWEET11 overexpression did not affect herbivory-inhibited sugar export of mature leaves (boxplot, n = 7). When elicited mature leaves (node 2) were ^{13}C-labelled prior to treatments, SWEET11 overexpression had no effects on retained $\delta^{13}C$ levels in elicited leaves either at constitutive stage (control) or induced stage (Herbivory elicitation, W + OS), while herbivory elicitation increased retained ^{13}C levels of elicited leaves 12 h after elicitation.

(E – G) SWEET11 overexpression did not affect potato plant's tolerance of *P. operculella* herbivory. *P. operculella* herbivory (leaves at nodes 2, 3, 4 each attacked by 4 larvae) induced growth compensation of immature leaves (node -2) 9 d after start of attacks in SWEET11-OE plants (**E**; mean ± SEM, n = 11 – 15). *P. operculella* herbivory did not result in aboveground (**F**) and tuber (**G**) biomass loss 45 d after start of herbivory in WT and SWEET11-OE plants (boxplot, n = 10 – 13).

In the boxplots, triangles refer to mean values and grey points refer to data points. Asterisks indicate significant difference between control and treatment groups (Student's t test, *$p < 0.05$, **$p < 0.01$, ***$p < 0.001$; ns, not significant). Two-way ANOVA is used to analyze the interactive effect of genotype and treatment (*$p < 0.05$, **$p < 0.01$, ***$p < 0.001$).

DAA, day after start of attacks; DW, dry weight.

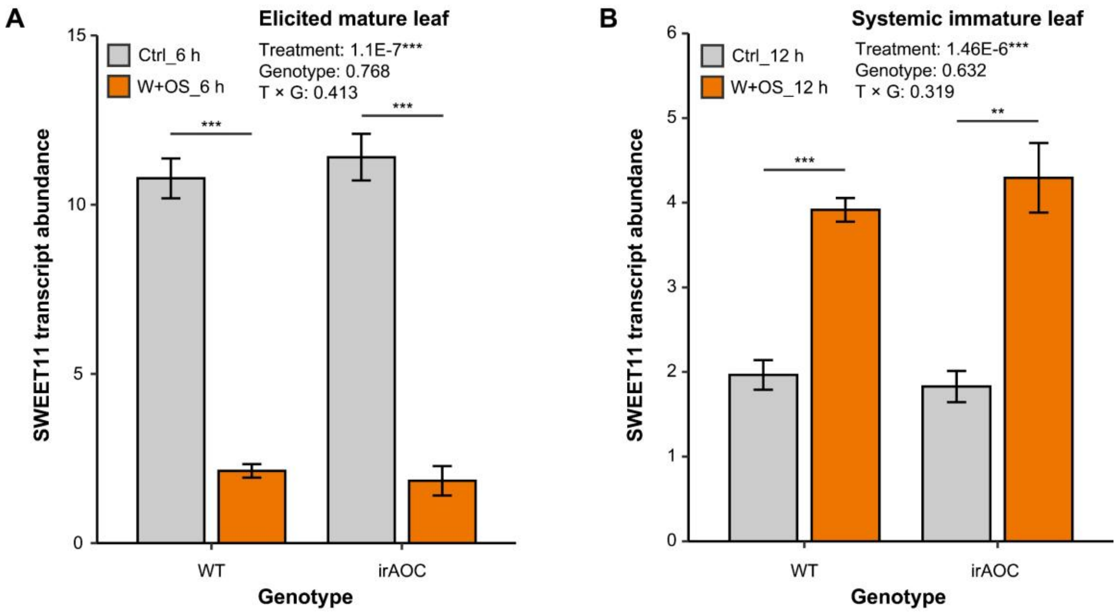

Fig. S9. The transcriptional regulation of SWEET11 in response to herbivory is independent of jasmonic acid signaling.

(A) Herbivory elicitation (W+OS) still repressed SWEET11 transcript abundance in elicited mature leaves (node 2) of JA-biosynthesis-deficient (irAOC) plants 6 h after elicitation (mean ± SEM, n = 3).

(B) Herbivory elicitation (W+OS) of mature leaves (nodes 2, 3, 4) increased SWEET11 transcript abundance in systemic immature leaves (node -2) of JA-biosynthesis-deficient (irAOC) plants 12 h after elicitation (mean ± SEM, n = 3).

Asterisks indicate significant difference between control and treatment (Student's t test, *$p < 0.05$, **$p < 0.01$, ***$p < 0.001$). The interaction effect of treatment and genotype was analyzed using two-way ANOVA (*$p < 0.05$, **$p < 0.01$, ***$p < 0.001$).

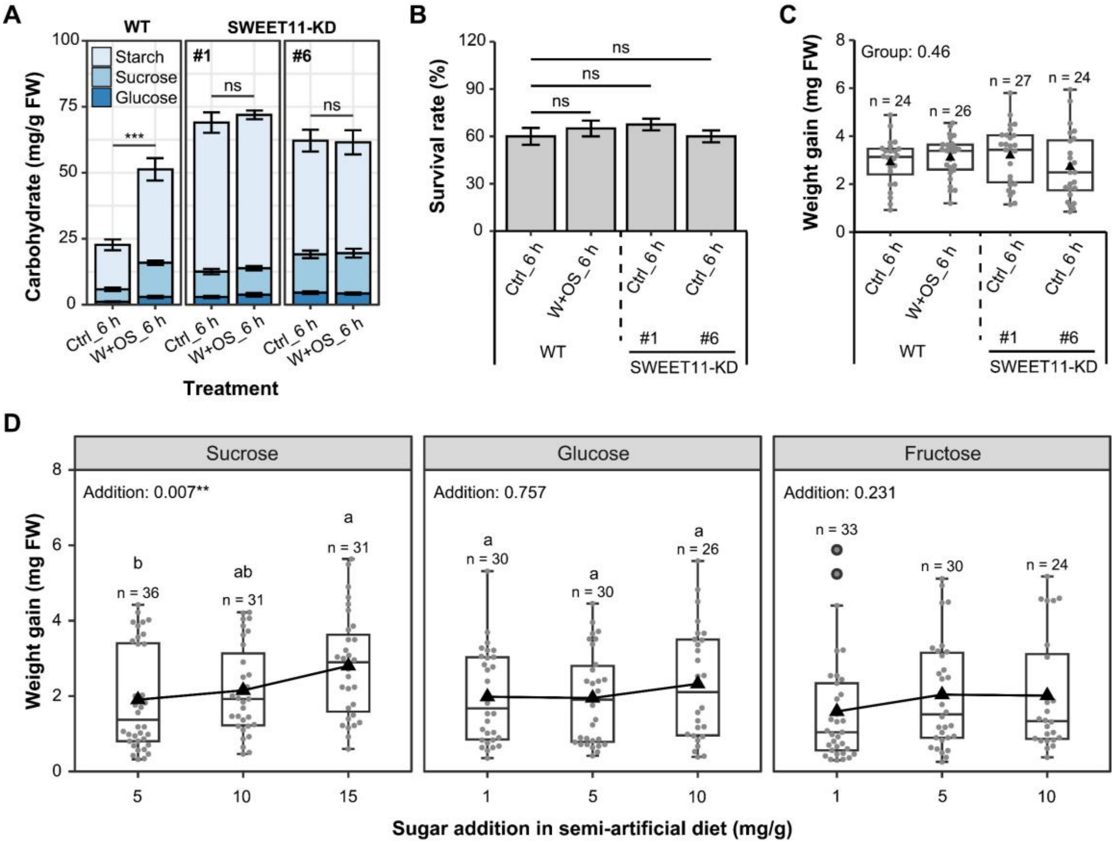

Fig. S10. Increasing carbohydrate levels in semi-artificial diets (semi-AD) did not reduce larval performance.

(A) Herbivory elicitation (W+OS) increased carbohydrate contents of elicited mature leaf (node 2) 6 h after elicitation through SWEET11 (mean ± SEM, n = 5).

(B – C) Performance of larvae fed a semi-ADs prepared from WT or SWEET11-KD mature leaves did not differ. Diets did not affect larval survival rates (**B**; mean ± SEM, n = 8) or weight gain (**C**; boxplot, n = 24 – 27)

(D) Supplementing the semi-AD prepared from WT mature leaves with additional sugar did not reduce larval weight gain (boxplot, n = 24 – 36).

In boxplots, triangles refer to mean values, and grey points refer to data points. Asterisks indicate significant difference between groups (Student's t test, *$p < 0.05$, **$p < 0.01$, ***$p < 0.001$; ns, not significant). Different letters indicate significant differences among different diets (LSD *post hoc* multiple comparisons following one-way ANOVA, *$p < 0.05$, **$p < 0.01$, ***$p < 0.001$).

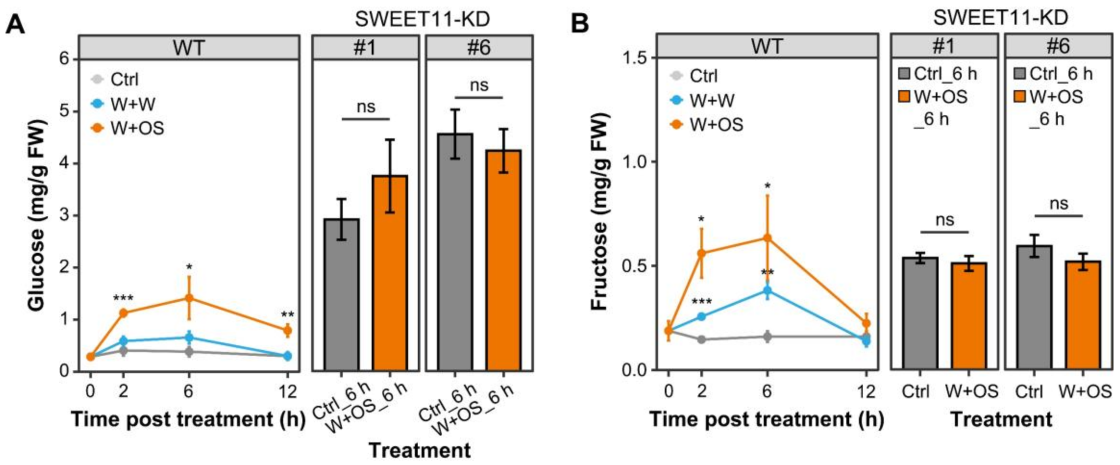

Fig. S11. Herbivory elicitation rapidly elevates hexose levels in elicited mature leaves through SWEET11.

Herbivory elicitation (W + OS) rapidly elevated glucose (**A**; mean ± SEM, n = 5) and fructose (**B**; mean ± SEM, n = 4 – 5) levels in elicited mature leaves (node 2) within 6 h of elicitation. Hexose levels were constitutively elevated and herbivory elicitation did not increase glucose and fructose levels of elicited mature leaves 6 h after elicitation in SWEET11-KD plants.

Asterisks indicate significant difference between treatment and control groups (Student's t test, *$p < 0.05$, **$p < 0.01$, ***$p < 0.001$; ns, not significant).

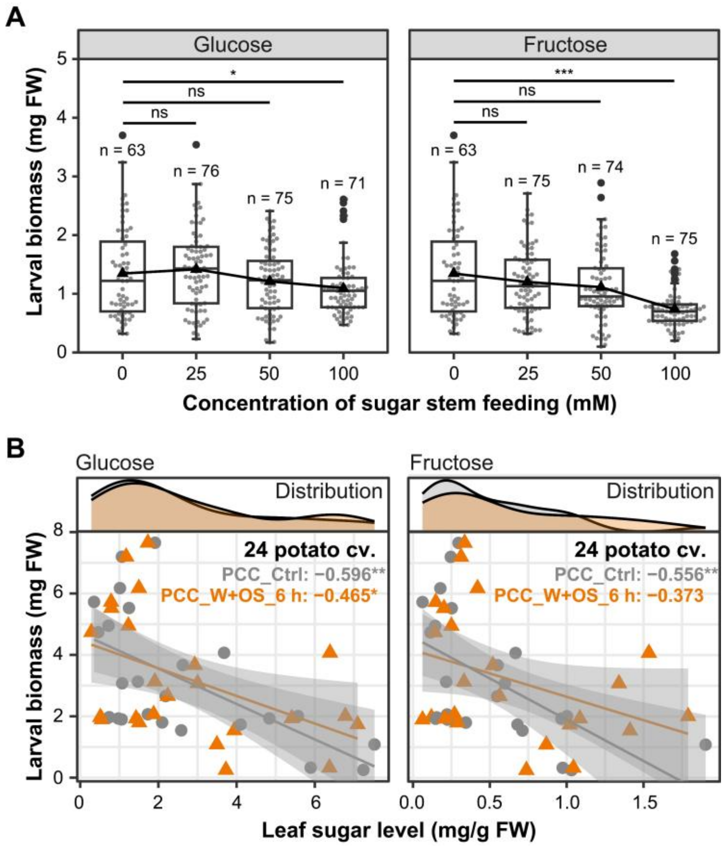

Fig. S12. Hexoses (glucose and fructose) enhance herbivore resistance.

(A) Exogenous supplementation of hexose (glucose and fructose) reduced larval biomass (boxplot, n = 63 – 76). Exogenous hexose was supplemented to mature leaves (nodes 1, 2) using stem feeding method for 6 h, followed by bioassays.

Triangles refers to mean values; black dots refer to outliers; grey points refer to data points.

Asterisks indicate significant difference between concentration groups (Mann-Whitney U test, *$p < 0.05$, **$p < 0.01$, ***$p < 0.001$; ns, not significant).

(B) Mature leaf hexose (glucose and fructose) levels were negatively correlated with larval performance across 24 potato cultivars. Upper panel: distribution of mature leaf (node 2) hexose levels in control and herbivory elicitation (W+OS) treated samples (6 h after treatment). Lower panel: correlation analysis between larval biomass (n = 30 – 71) and mature leaf hexose levels (n = 4 – 5) among 24 potato cultivars [Pearson correlation coefficient (PCC), *$p < 0.05$, **$p < 0.01$, ***$p < 0.001$; ribbons refer to 95% confidence intervals].

FW, fresh weight.

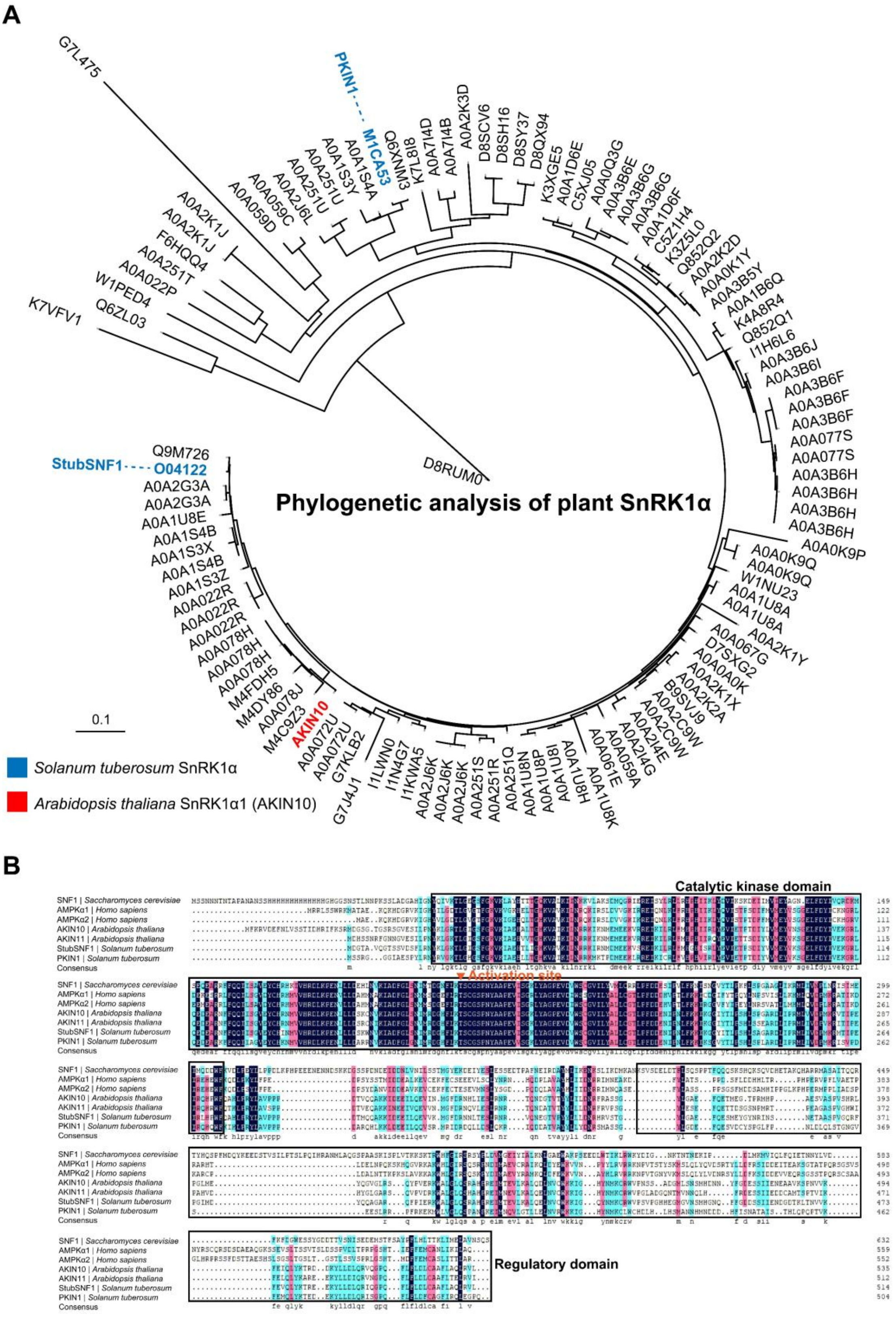

Fig. S13. Phylogenetic analysis and protein multiple alignments reveal that potato StubSNF1 is an ortholog of *Arabidopsis thaliana* SnRK1α1.1 (AKIN10).

(A) Phylogenetic analysis of AKIN10 ortholog candidates across the plant kingdom.

AKIN10 is highlighted in red and two potato ortholog candidates (StubSNF1 and PKIN1) are highlighted in blue. Phylogenetic tree was constructed using Maximum Likelihood method with 1000 bootstrap replicates.

(B) Protein multiple alignment of *Saccharomyces cerevisiae* SNF1, *Homo sapiens* AMPK1α, *Arabidopsis thaliana* SnRK1α and *Solanum tuberosum* SnRK1α revealed that SNF1-related protein kinases were highly conserved in their catalytic kinase domain.

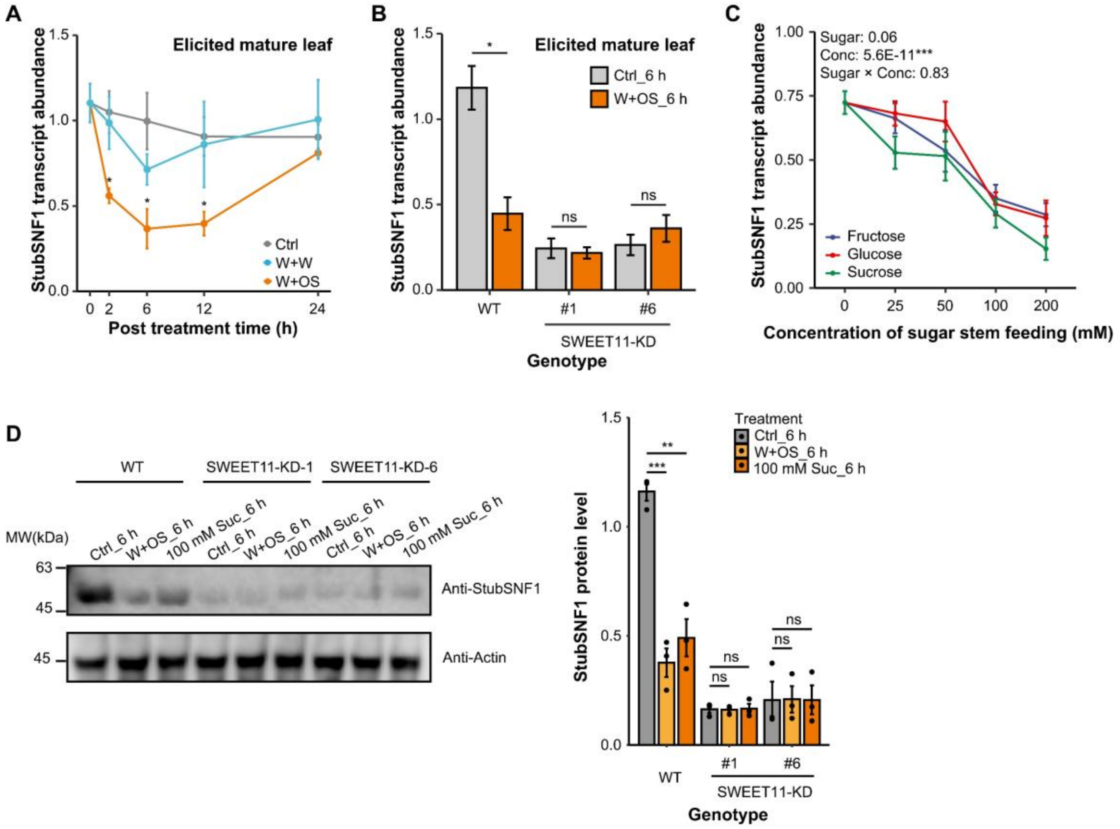

Fig. S14. Herbivory elicitation represses StubSNF1 transcript accumulations and protein abundances via SWEET11-mediated sugar in elicited mature leaf.

(A) Herbivory elicitation (W+OS) repressed relative StubSNF1 transcript accumulation in elicited mature leaves (node 2) within 12 h (mean ± SEM, n = 3).

(B) Herbivory elicitation (W+OS) repressed StubSNF1 transcript accumulations in elicited mature leaves (node 2) 6 h after elicitation through SWEET11 (mean ± SEM, n = 3).

(C) Exogenous sugars repressed StubSNF1 transcript accumulations in mature leaves (nodes 1, 2) (mean ± SEM, n = 3). The effects of sugar and concentration (Conc) are analyzed using two-way ANOVA (*$p < 0.05$, **$p < 0.01$, ***$p < 0.001$).

(D) Herbivory elicitation (W+OS) and sucrose feeding (100 mM) reduced StubSNF1 protein abundances of elicited mature leaves (node 2) 6 h after treatment in WT plants but not in SWEET11-knockdown plants. Left: representative images. Right: quantification.

Asterisks indicate significant difference between treatment and control (Student's t test, *$p < 0.05$, **$p < 0.01$, ***$p < 0.001$; ns, not significant).

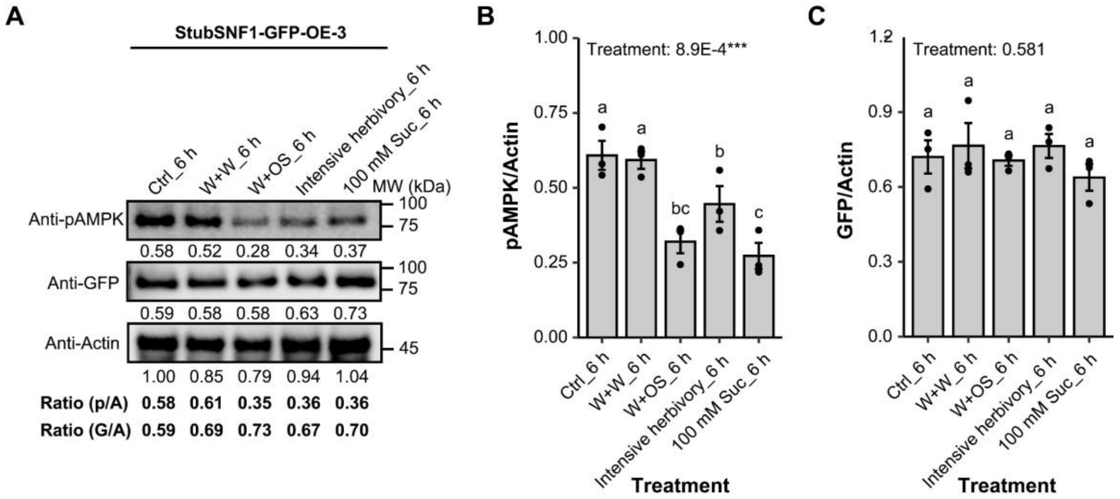

Fig. S15. Herbivory elicitation and exogenous sucrose suppress StubSNF1 activation.

(A) Activated StubSNF1-GFP (anti-pAMPK) and total StubSNF1-GFP (anti-GFP) levels of mature leaves (node 2) were analyzed in StubSNF1-GFP-OE plants 6 h after different treatments (W+W, W+OS, Intensive herbivory, 100 mM Suc) using immunoblotting. Actin served as an internal standard. Intensive herbivory, 30 fourth-instar larvae feeding a mature leaf for 1 h.

(B) Quantification of activated StubSNF1-GFP using Image J (mean ± SEM, n = 3).

(C) Quantification of total StubSNF1-GFP using Image J (mean ± SEM, n = 3).

Different letters indicate significant difference between treatments (LSD *post hoc* multiple comparisons following one-way ANOVA, *$p < 0.05$, **$p < 0.01$, ***$p < 0.001$).

Intensive herbivory, 30 4th larvae feeding for 1 h.

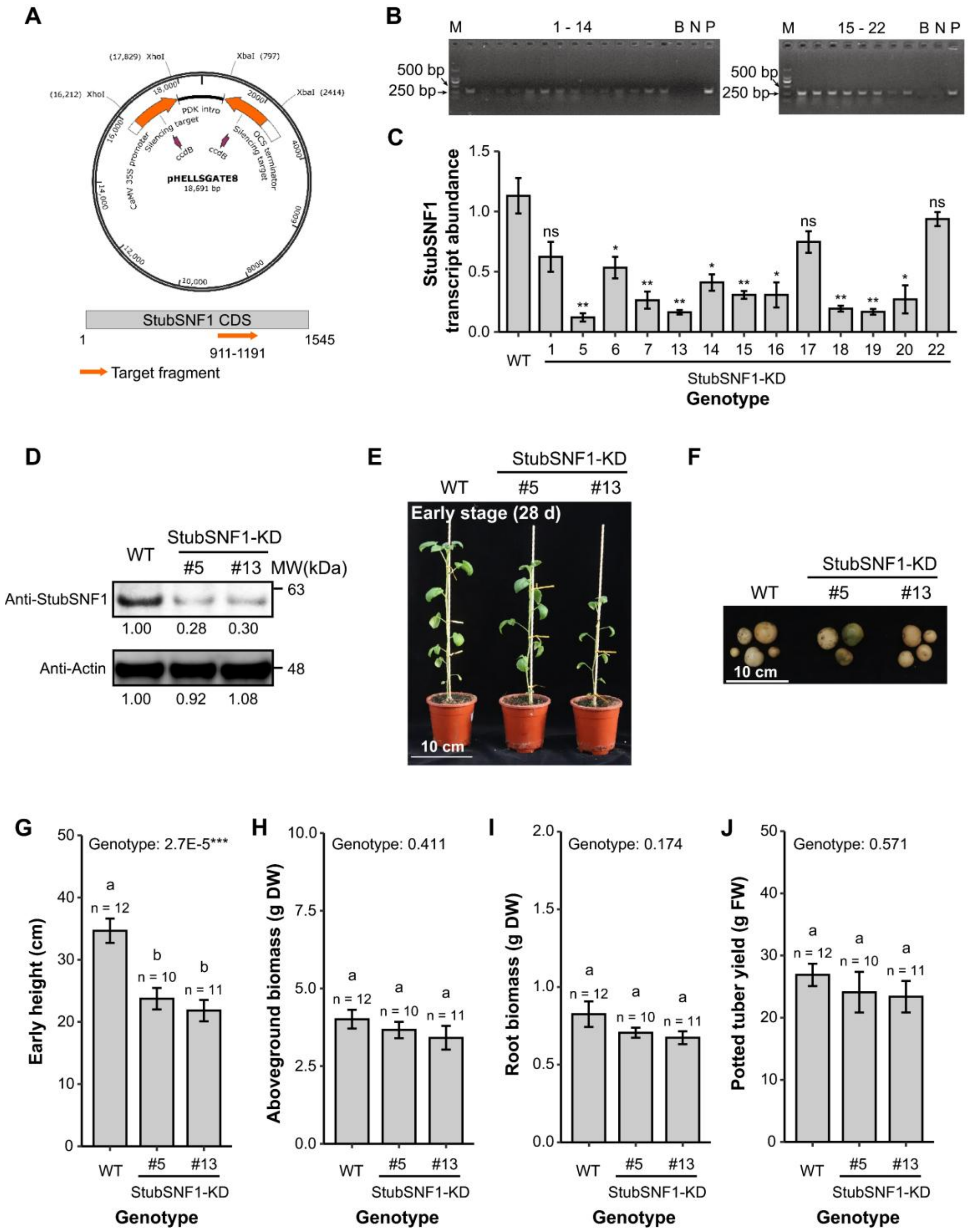

Fig. S16. Construction and phenotypic characterization of StubSNF1-KD plants.

(A – D) Construction of StubSNF1-KD plants.

(A) Design of inverted-repeat transformation vector for knocking down StubSNF1 by RNAi. A fragment of StubSNF1 CDS (911 – 1191) was cloned into pHELLSGATE8 as showed. Plasmid was transformed into potato plants by *Agrobacterium*-mediated transformation.

(B) Detection of transformed plants using PCR. Screening marker NPTII gene was

detected after transformation. M: marker; B: blank; N: negative control; P: positive control.

(C) Quantification of StubSNF1 knockdown efficiency using qRT-PCR (mean ± SEM, n = 3). The silencing efficiency of StubSNF1 was assessed by quantifying its relative expression in mature leaves (node 2) of 28-d plant.

(D) Detection of StubSNF1 knockdown efficiency at the protein level. StubSNF1 protein was measured using immunoblotting in mature leaves (node 2) of 28-d plant.

Asterisks indicate significant difference between KD and WT plants (Student's t test, *$p < 0.05$, **$p < 0.01$, ***$p < 0.001$; ns, not significant).

(E – J) Phenotypic characterization of StubSNF1-KD plants.

(E) Representative plants at early growth stage (28 d after transplanting).

(F) Tubers after harvesting (75 d after transplanting).

(G) StubSNF1 knockdown resulted in a decrease in plant height 28 d after transplanting (mean ± SEM, n = 10 – 12).

(H) StubSNF1 knockdown did not affect aboveground biomass 75 d after transplanting (DW; mean ± SEM, n = 10 – 12).

(I) StubSNF1 knockdown did not affect root biomass 75 d after transplanting (DW; mean ± SEM, n = 10 – 12).

(J) StubSNF1 knockdown did not affect potted tuber yield 75 d after transplanting (FW; mean ± SEM, n = 10 – 12).

Different letters indicate significant differences between genotypes (LSD *post hoc* multiple comparisons following one-way ANOVA, *$p < 0.05$, **$p < 0.01$, ***$p < 0.001$).

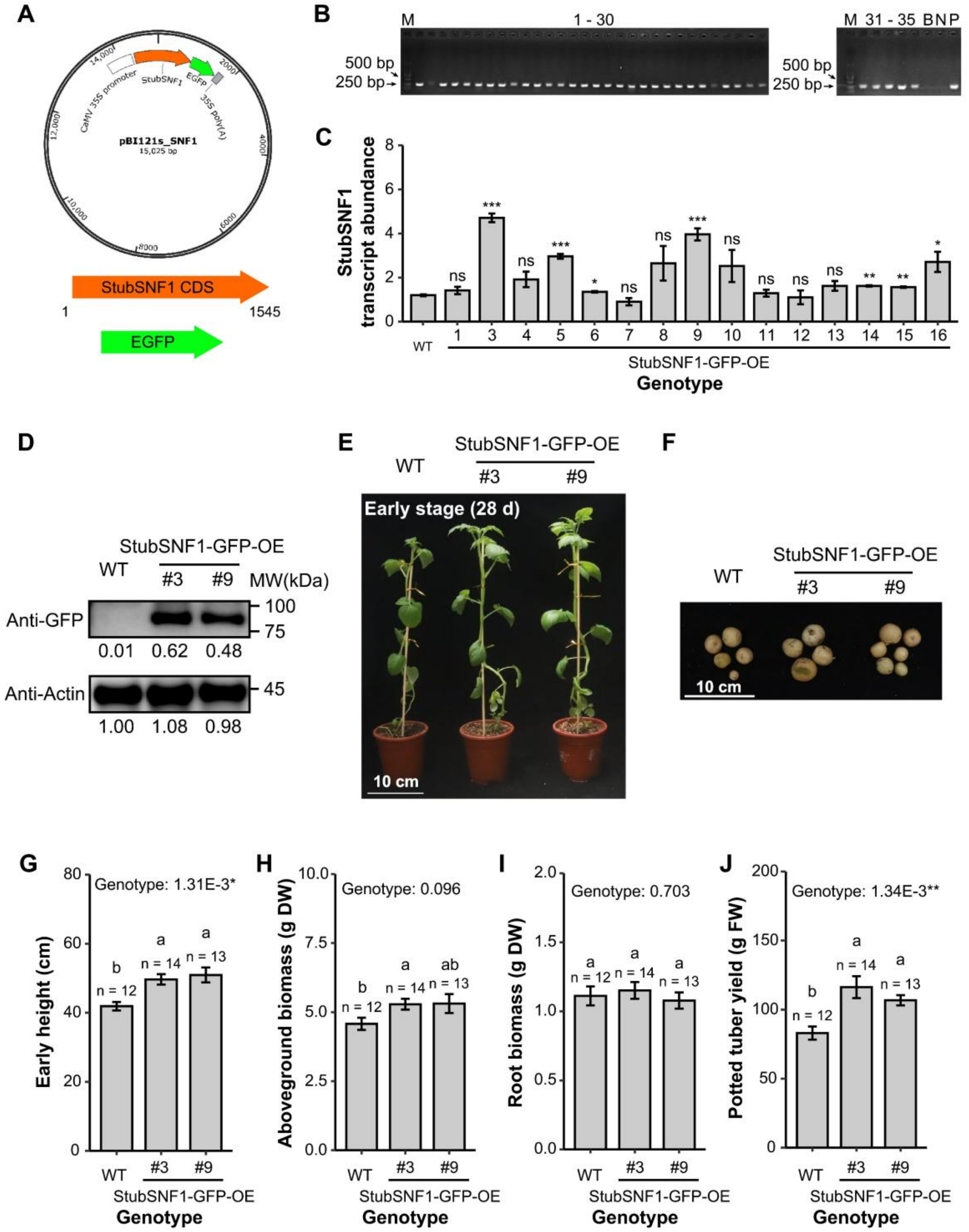

Fig. S17. Construction and phenotypic characterization of StubSNF1-GFP-OE plants.

(A – D) Construction of StubSNF1-GFP-OE plants.

(A) Design of StubSNF1 overexpression transformation construct. StubSNF1 CDS and GFP were cloned into pBI121s as shown. Plasmid was transformed into potato plant by *Agrobacterium*-mediated transformation.

(B) Detection of transformed plants using PCR. Screening marker NPTII gene was

detected after transformation. M: marker; B: blank; N: negative control; P: positive control.

(C) Quantification of StubSNF1 overexpression efficiency using qRT-PCR (mean ± SEM, n = 3). The overexpression efficiency of StubSNF1 was assessed by quantifying its relative expression in mature leaf (node 2) of 28-d plant.

(D) Detection of StubSNF1 overexpression efficiency at the protein level. StubSNF1 protein was measured using immunoblotting in mature leaf (leaf 2) of 28-d plant.

Asterisks indicate significant difference between OE and WT plants (Student's t test, **p* < 0.05, ***p* < 0.01, ****p* < 0.001; ns, not significant).

(E – J) Phenotypic characterization of StubSNF1-GFP-OE plants.

(D) Representative plants at early growth-stage (28 d after transplanting).

(F) Tubers after harvesting (75 d after transplanting).

(G) StubSNF1 overexpression resulted in an increase in plant height 28 d after transplanting (mean ± SEM, n = 12 – 14).

(H) StubSNF1 overexpression did not affect aboveground biomass 75 d after transplanting (DW; mean ± SEM, n = 12 – 14).

(I) StubSNF1 overexpression did not affect root biomass 75 d after transplanting (DW; mean ± SEM, n = 12 – 14).

(J) StubSNF1 overexpression resulted in an increase in potted tuber yield 75 d after transplanting (FW; mean ± SEM, n = 12 – 14).

Different letters indicate significant difference between genotypes (LSD *post hoc* multiple comparisons following one-way ANOVA, **p* < 0.05, ***p* < 0.01, ****p* < 0.001).

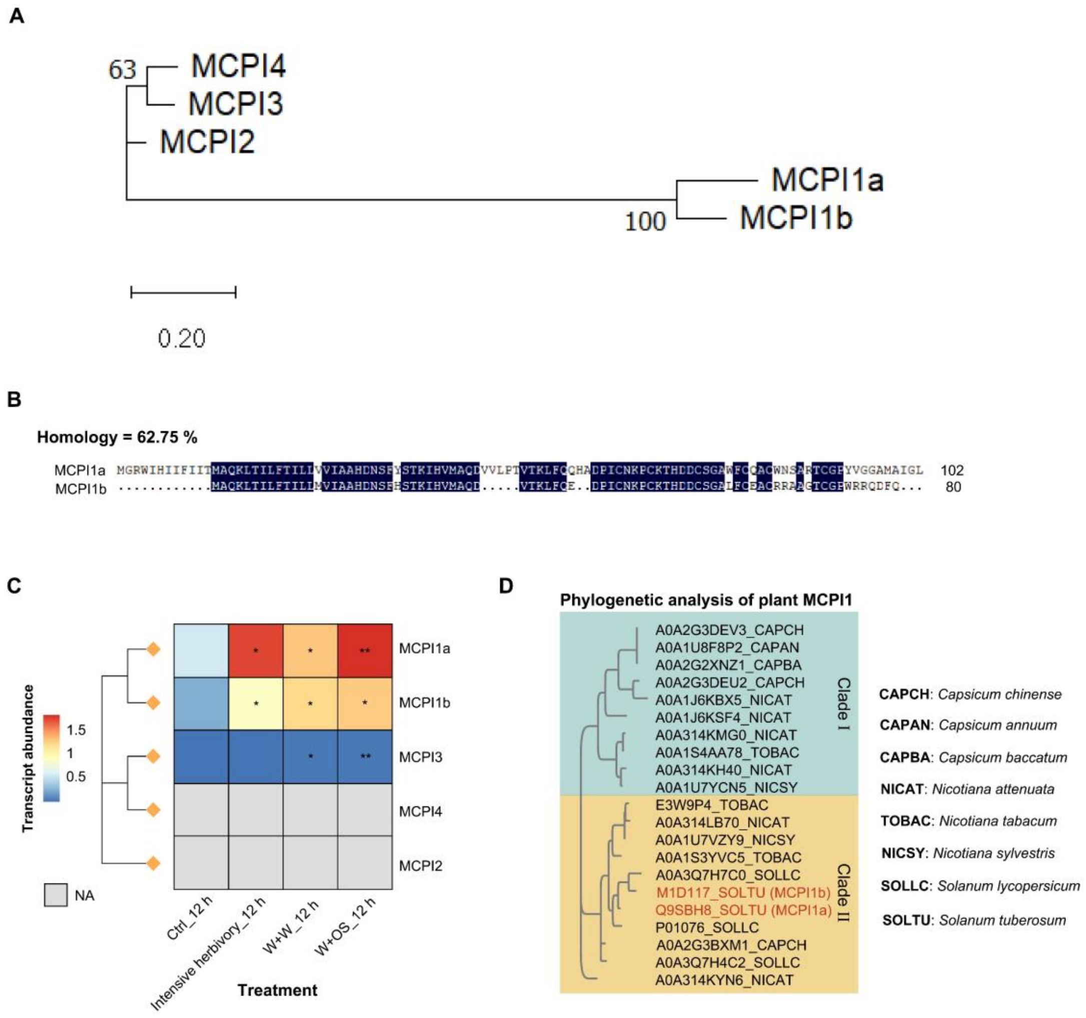

Fig. S18. Analysis of potato MCPIs.

(A) Phylogenetic analysis of potato MCPIs.

(B) Protein alignment of MCPI1a and MCPI1b using ClustalW.

(C) Transcript abundances of potato MCPI genes in elicited mature leaves (node 2) 12 h after treatments (W+W, W+OS, Intensive herbivory). Left panel: phylogenetic analysis of potato MCPIs. Right panel: transcript abundance.

Asterisks indicate significant difference between treatment and control (Student's t test, *$p < 0.05$, **$p < 0.01$, ***$p < 0.001$).

(D) Identification of MCPI1 as a Solanaceae-specific protein. BLAST analysis using StMCPI1a's catalytic region and phylogenetic analysis reveal that MCPI1s are Solanaceae-specific.

Phylogenetic tree was constructed using Maximum Likelihood method with 1000 bootstrap replicates. MCPI: metallocarboxypeptidase inhibitor. NA: not available.

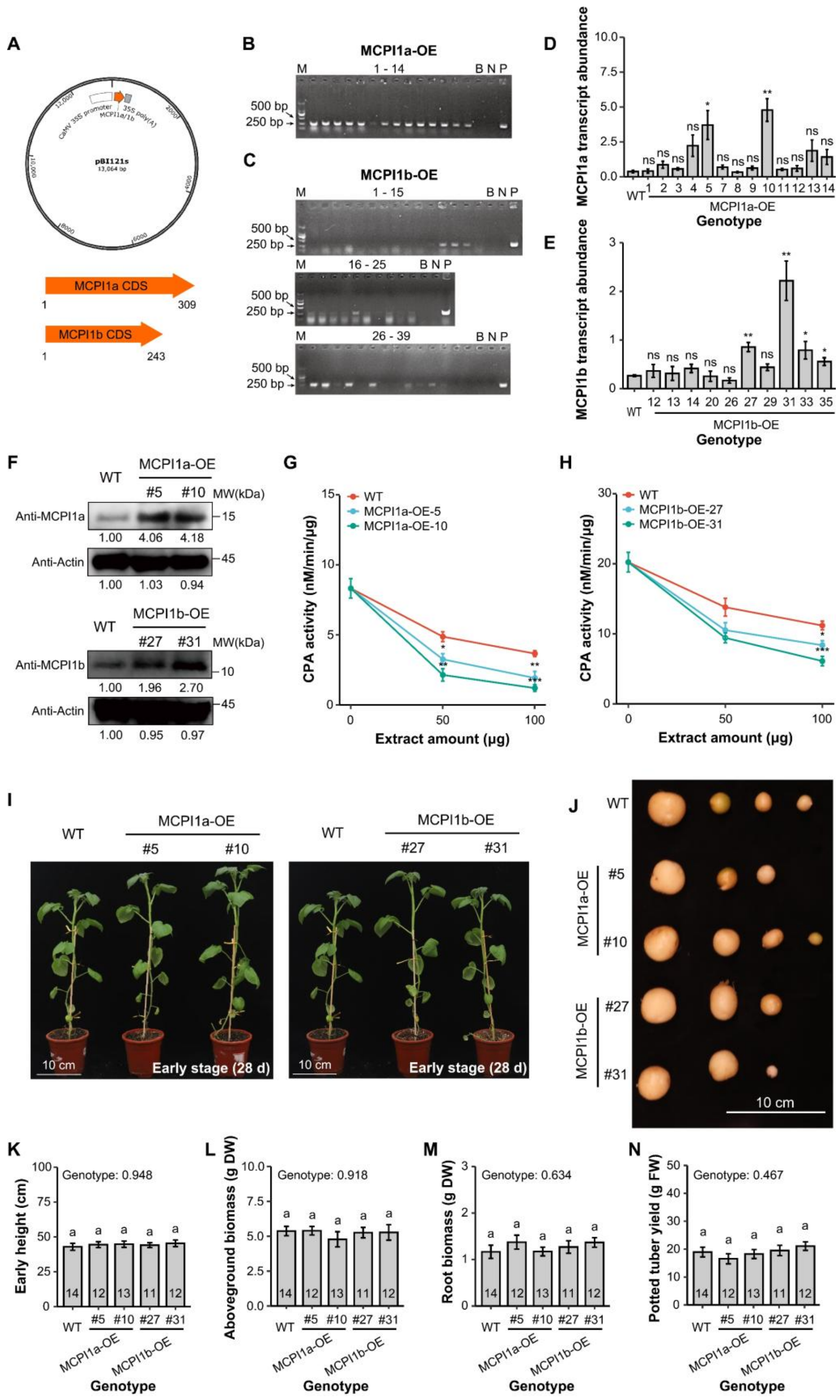

Fig. S19. Construction and phenotypic characterization of MCPI1a-OE and MCPI1b-OE plants.

(A – H) Construction of MCPI1a-OE and MCPI1b-OE plants.

(A) Design of MCPI1a and MCPI1b overexpression transformation constructs. MCPI1a or MCPI1b CDS was cloned into pBI121s as shown. Plasmids were transformed into potatoes by *Agrobacterium*-mediated transformation.

(B – C) Detection of transformed plants using PCR (**B**: MCPI1a-OE plants; **C**: MCPI1a-OE plants). Screening marker NPTII gene was detected after transformation. M: marker; B: blank; N: negative control; P: positive control.

(D – E) Detection of overexpression efficiency using qRT-PCR (mean ± SEM, n = 3). The overexpression efficiency of was assessed by quantifying its relative expression in mature leaves (node 2) of 28-d plants. **D**: MCPI1a-OE plants. **E**: MCPI1a-OE plants.

(F) Quantification of overexpression efficiency at the protein level. MCPI1a or MCPI1b proteins were measured using immunoblotting of mature leaves (node 2) of 28-d plants.

(G – H) MCPI1a (**G**) or MCPI1b (**H**) overexpression resulted in increased inhibition of bovine carboxypeptidase A (CPA) by leaf extract (mean ± SEM, n = 5).

Asterisks indicate significant difference between OE and WT plants (Student's t test, *$p < 0.05$, **$p < 0.01$, ***$p < 0.001$; ns, not significant).

(I – N) Phenotypic characterization of MCPI1a-OE and MCPI1b-OE plants.

(I) Representative plants at early growth stage (28 d after transplanting).

(J) Tubers after harvesting (75 d after transplanting).

(K) MCPI1a or MCPI1b overexpression did not affect plant height 28 d after transplanting (mean ± SEM, n = 12 – 14; n was displayed on bar).

(L) MCPI1a or MCPI1b overexpression did not affect aboveground biomass 75 d after transplanting (DW; mean ± SEM, n = 12 – 14; n was displayed on bar).

(M) MCPI1a or MCPI1b overexpression did not affect root biomass 75 d after transplanting (DW; mean ± SEM, n = 12 – 14; n was displayed on bar).

(N) MCPI1a or MCPI1b overexpression did not affect potted tuber yield 75 d after transplanting (FW; mean ± SEM, n = 12 – 14; n was displayed on bar).

Different letters indicate significant differences between genotypes (LSD *post hoc* multiple comparisons following one-way ANOVA, *$p < 0.05$, **$p < 0.01$, ***$p < 0.001$).

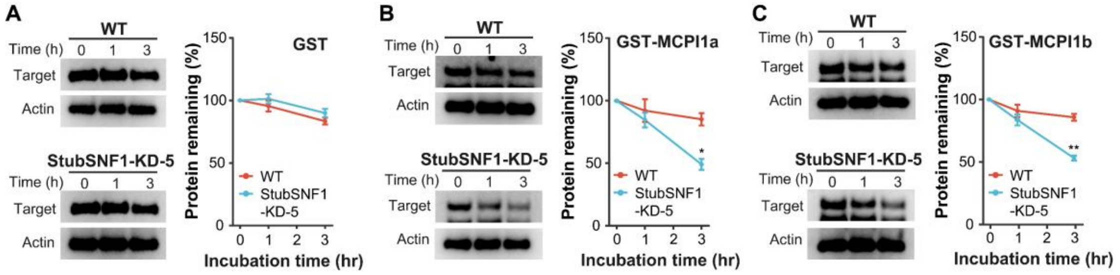

Fig. S20. Cell-free degradation assays demonstrate that StubSNF1 promotes the stability of MCPI1a/b.

(A) Degradation assays of GST proteins in protein extracts (leaf node 2) of WT and StubSNF1-KD-5 plants (mean ± SEM, n = 3).

(B) Degradation assays of GST-MCPI1a protein in protein extracts (leaf node 2) of WT or StubSNF1-KD-5 plants (mean ± SEM, n = 3).

(C) Degradation assays of GST-MCPI1b proteins in protein extracts (leaf node 2) of WT or StubSNF1-KD-5 plants (mean ± SEM, n = 3).

Left panel: representative immunoblotting image. Right panel: protein quantification using Image J. Asterisks indicate significant difference between WT and StubSNF1-KD-5 groups (Student's t test, *$p < 0.05$, **$p < 0.01$, ***$p < 0.001$).

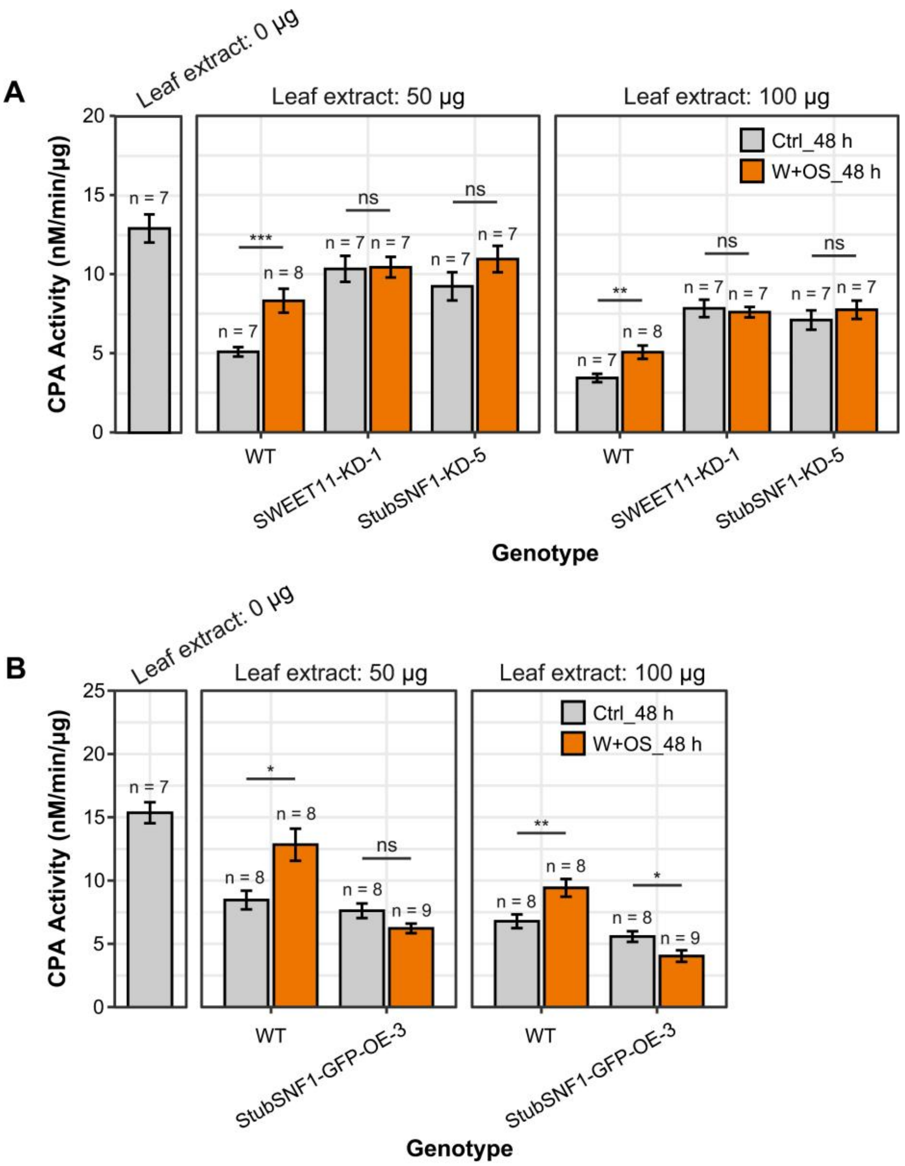

Fig. S21. The inhibition of bovine carboxypeptidase A (CPA) by extracts of elicited mature leaves closely mirrors changes in MCPI1a and MCPI1b protein levels following herbivory elicitation (W+OS).

(A) Herbivory elicitation (W+OS) reduced the inhibition of CPA by extracts of elicited mature leaves (node 2; 48 h after elicitation) in WT plants, while the reduction was not observed in SWEET11-KD or StubSNF1-KD plants (mean ± SEM, n = 7 – 8).

(B) Herbivory elicitation (W+OS) increased the inhibition of CPA by extracts of elicited mature leaves (node 2; 48 h after elicitation) in StubSNF1-GFP-OE-3 plants (mean ± SEM, n = 7 – 9).

Asterisks indicate significant difference between control and treatment (Student's t test, *$p < 0.05$, **$p < 0.01$, ***$p < 0.001$; ns, not significant).

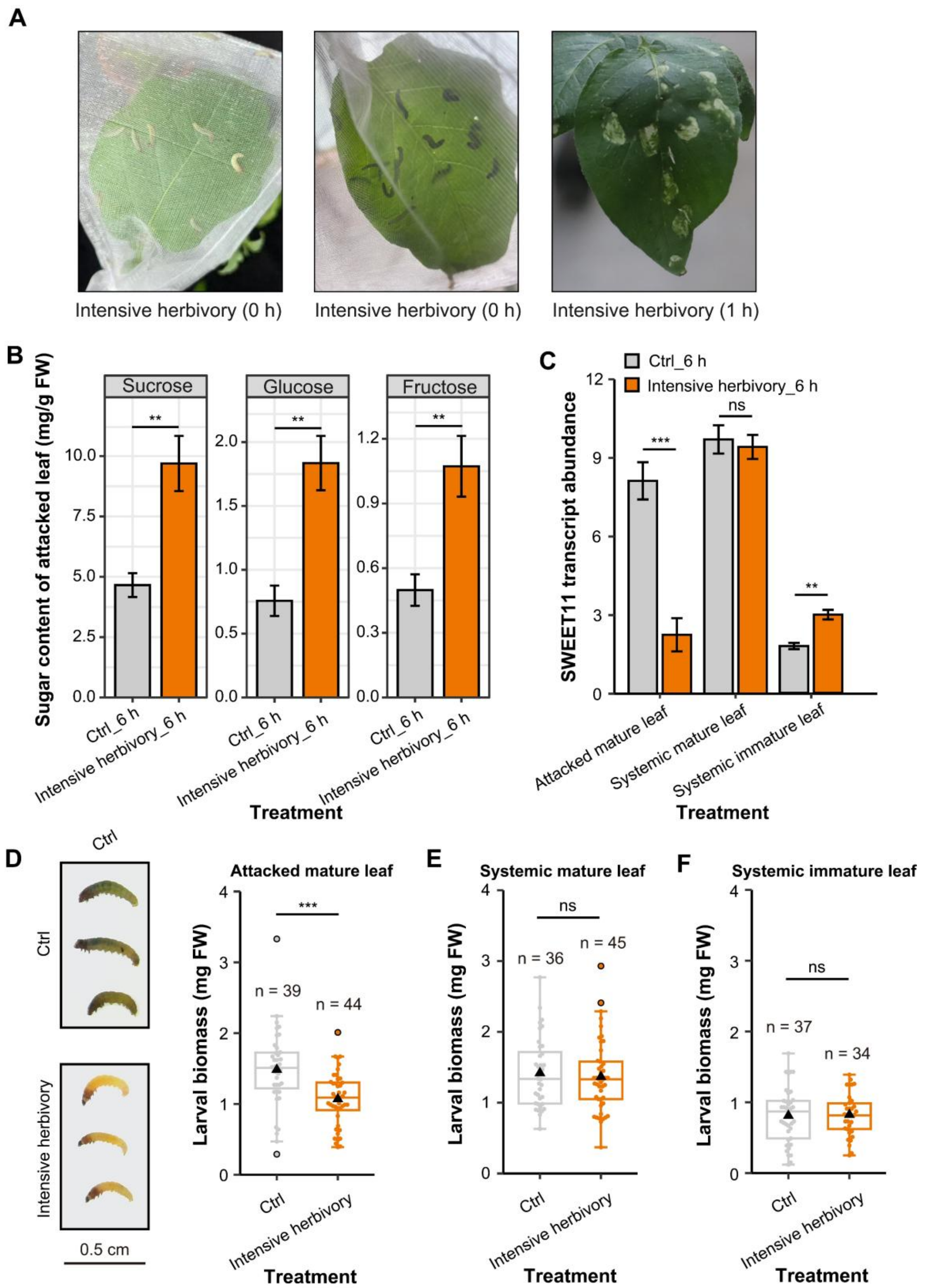

Fig. S22. Potato plants elevate sugar levels of attacked mature leaves, tissue-specifically regulate SWEET11, and activate resistance responses of attacked mature leaves following intensive herbivore damage treatments of mature leaves. **(A)** An hour of intensive herbivory caused significant damage to mature potato leaves.

(B) Intensive herbivory elevated 3 sugars (sucrose, glucose, fructose) of attacked mature leaves (node 2) 6 h after herbivory.

(C) Intensive herbivory of mature leaves (nodes 2, 3, 4) repressed SWEET11 transcript abundance in attacked mature leaves (node 2) and increased SWEET11 transcript abundance in systemic immature leaves (node -2) 6 h after herbivory.

(D) Intensive herbivory of mature leaves (nodes 2, 3, 4) reduced larval biomass when larvae were fed attacked mature leaves (node 2) (**D**; boxplot, n = 39 – 44), but did not affect larval biomass when larvae were fed systemic mature leaves (node 1) (**E**; boxplot, n = 36 – 45) or systemic immature leaves (node -2) (**F**; boxplot, n = 34 – 37).

Triangles refer to mean values, black circles refer to outliers, and points refers to data points. Asterisks indicate significant differences between control and treatment groups (Student's t test or Mann-Whitney U test, $*p < 0.05$, $**p < 0.01$, $***p < 0.001$)

Intensive herbivory, 30 4th larvae feeding for 1 h.

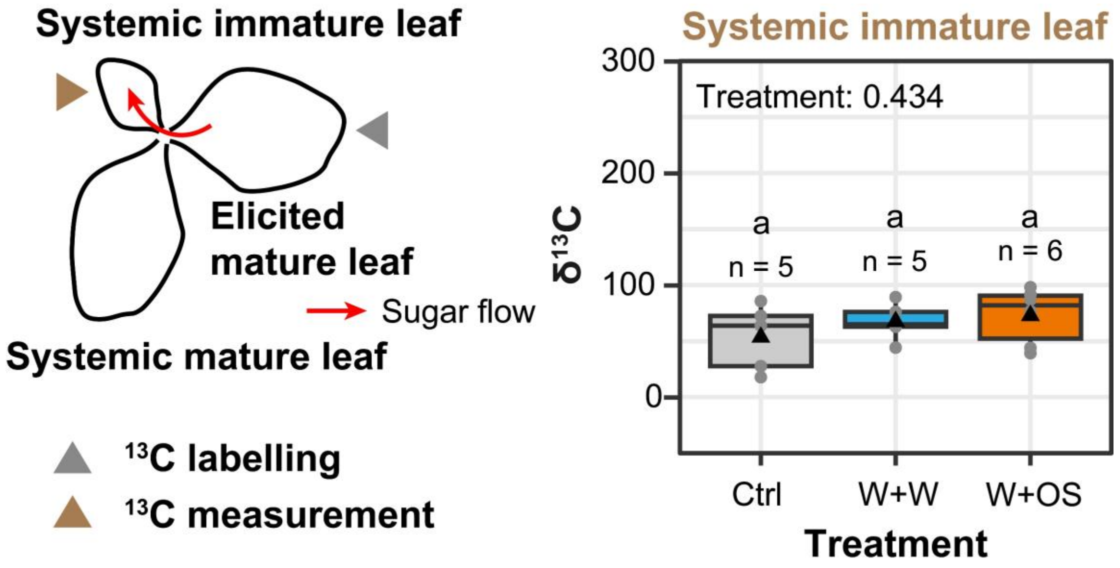

Fig. S23. Herbivory elicitation (W+OS) of mature leaves did not enhance sugar import from elicited leaves to systemic immature leaves.

When the elicited mature leaves (node 2) were labeled with ^{13}C prior to the treatments, ^{13}C levels in immature leaves (node -2) did not significantly increase 12 h after herbivory elicitation of mature leaves (nodes 2, 3, 4) (boxplot, n = 5 – 6).

In boxplots, triangles refer to mean values, and grey points refer to data points. Different letters indicate significant differences among treatments (LSD *post hoc* multiple comparisons following one-way ANOVA, $*p < 0.05$, $**p < 0.01$, $***p < 0.001$).

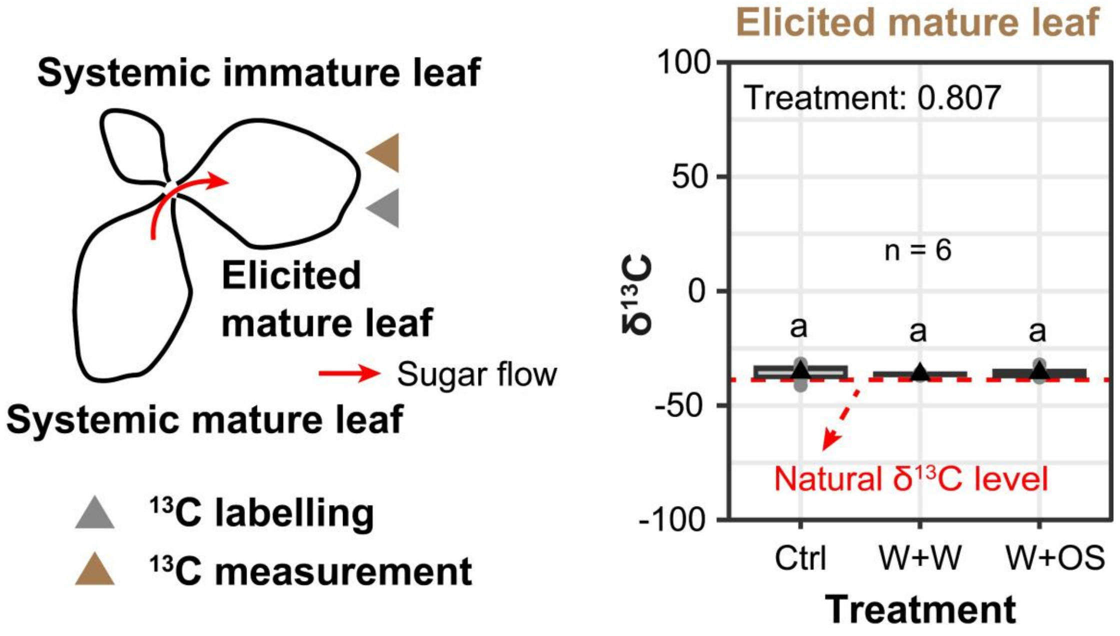

Fig. S24. Herbivory elicitation (W+OS) of mature leaves did not increase sugar import from systemic mature leaves to elicited mature leaves.

When the systemic mature leaves (node 1) were labeled with ^{13}C prior to the treatments, ^{13}C levels in elicited mature leaves (node 2) did not change and remained close to the natural $\delta^{13}C$ level 12 h after herbivory elicitation of mature leaves (nodes 2, 3, 4) (boxplot, n = 6). Red dashed line indicates natural ^{13}C level ($\delta^{13}C$ = -38.8).

In boxplots, triangles refer to mean values, and grey points refer to data points. Different letters indicate significant differences among treatments (LSD *post hoc* multiple comparisons following one-way ANOVA, *$p < 0.05$, **$p < 0.01$, ***$p < 0.001$).

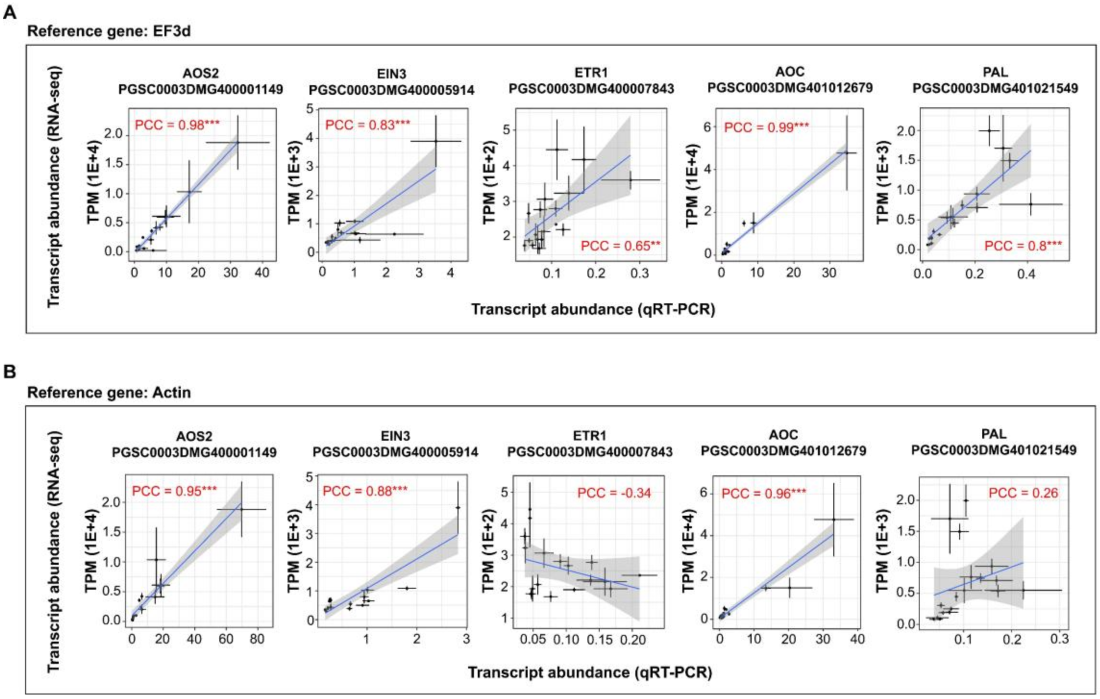

Fig. S25. Verifying data reliability of RNA-seq.

The transcript abundances of 5 phytohormone-related genes (*AOS2*, *EIN3*, *ETR1*, *AOC*, *PAL*) were quantified using qRT-PCR (**A**: *EF3d* as reference gene; **B**: *Actin* as reference gene). Pearson correlation analyses were performed between RNA-seq and qRT-PCR (PCC, Pearson correlation coefficient; *$p < 0.05$, **$p < 0.01$, ***$p < 0.001$).

AOS2, Allene oxide synthase 2; EIN3, Ethylene insensitive3; ETR1, Ethylene receptor 1; AOC, Allene oxide cyclase; PAL, Phenylalanine ammonia lyase; EF3d, Elongation factor 3d.

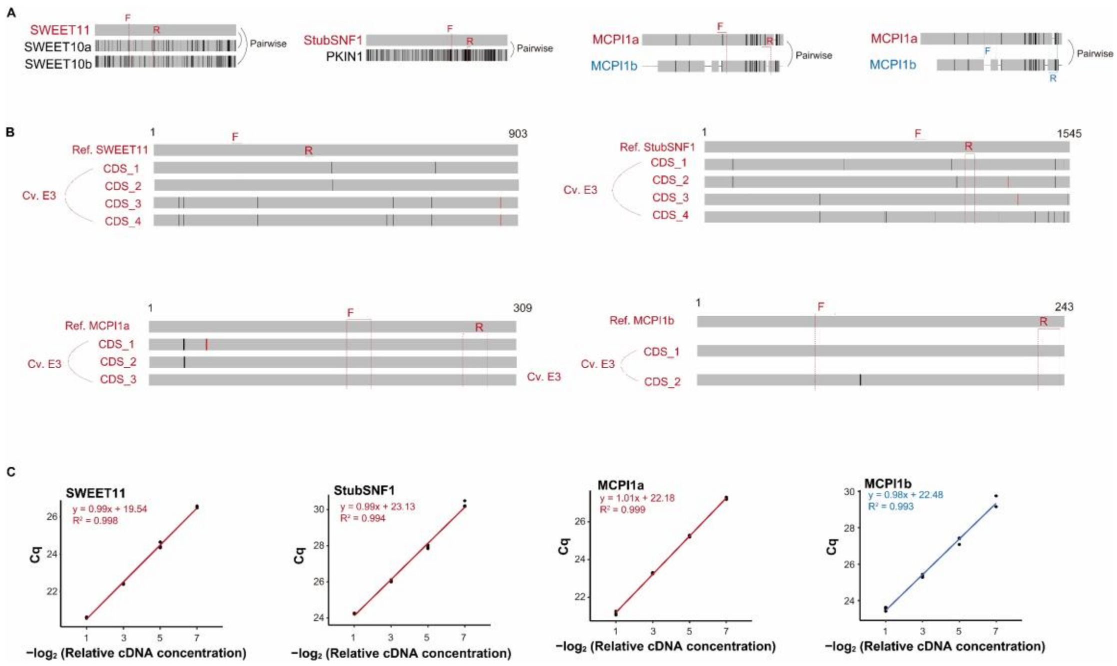

Fig. S26 Verifying efficiency of 4 main qRT-PCR primer pairs used in the study.

(A) CDS pairwise alignments between target genes and their closest paralogs. The positions of F and R were displayed as arrows. Grey bars indicate base agreements, black bars indicate base disagreements, and black lines indicate gaps.

(B) CDS pairwise alignments between reference genes and distinct cloned sequences (cultivar E3). The positions of F and R were displayed as arrows. Grey bars indicate base agreements, black bars indicate synonymous mutation, and red bars indicate nonsynonymous mutations.

(C) Estimating efficiency of qRT-PCR primers for SWEET11, StubSNF1, MCPI1a, MCPI1b transcript quantification. Leaf cDNA was serially diluted and qRT-PCR was conducted. Linear relationship between Cq value and -$\log_2$(Relative cDNA concentration) was analyzed. The relative concentration of undiluted cDNA was designated as 1.

F, forward primer; R, reverse primer.

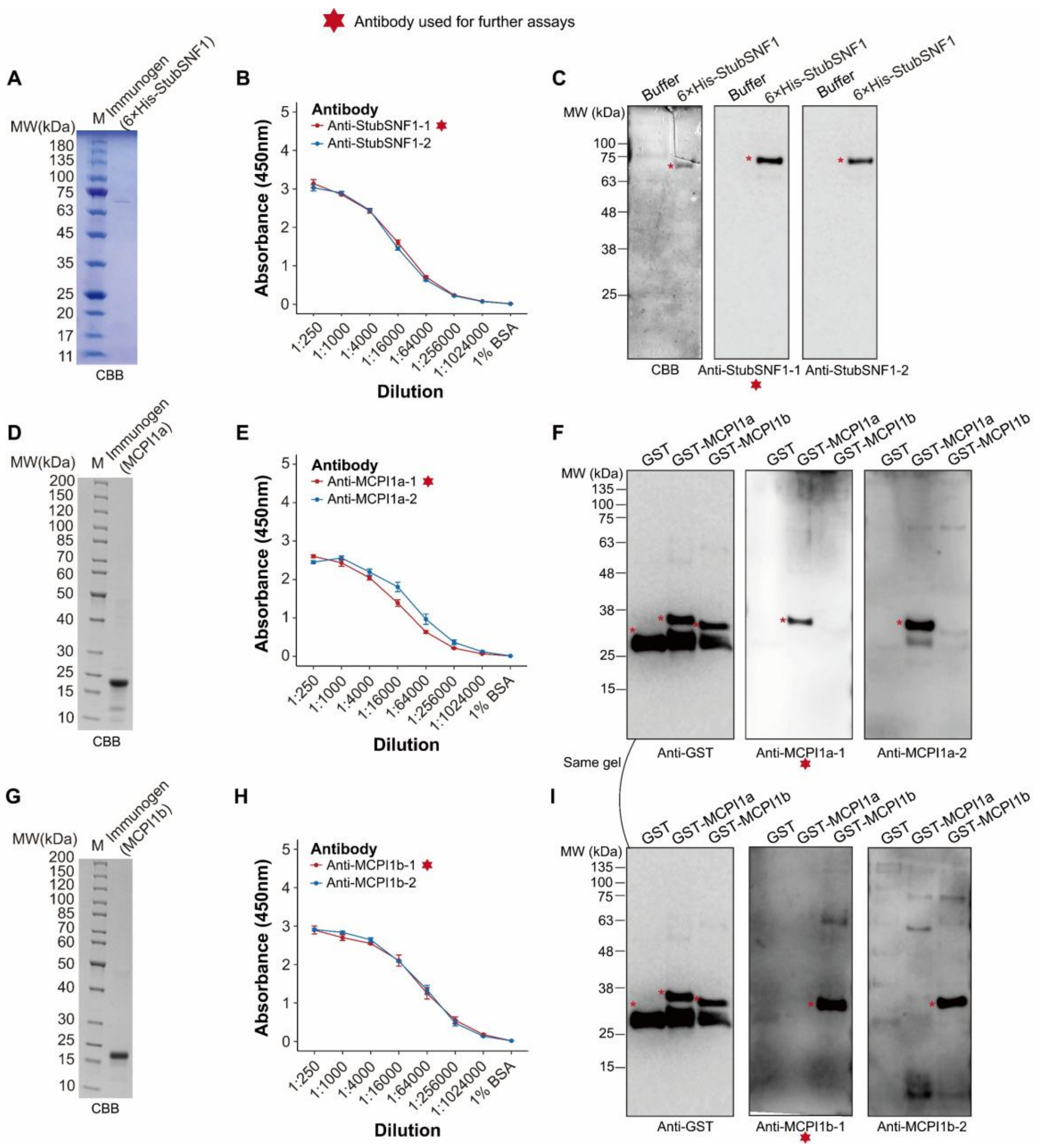

Fig. S27 Synthesizing and verifying Anti-StubSNF1, Anti-MCPI1a, and Anti-MCPI1b.

(A, D, G) Recombinant proteins were purified (see Methods section) and used as immunogens for synthesizing rabbit poly-antibody. Immunogen sequences were displayed in Supplementary Data S3.

(B, E, H) Measuring binding efficiency of synthesized antibodies to their immunogens using ELISA (mean ± SEM, n = 3). Two independent antibodies to an immunogen were independently synthesized in two rabbits. Purified antibodies (approx. 15 mg/mL) were serially diluted and their binding capacities were measured using ELISA (absorbance at 450 nm). 1 % BSA solution was used as negative control.

(C, F, I) Verifying binding capability of synthesized antibodies to their immunogens in immunoblotting assays. Proteins 6×His-StubSNF1, GST-MCPI1a, and GST-MCPI1b were detected by their antibodies using immunoblotting. Buffer (25 mM Tris-HCl, pH 7.4) or GST protein were used as negative control.

CBB, Coomassie brilliant blue; ELISA, enzyme-linked immunosorbent assay; BSA, bovine serum albumin. M, marker.

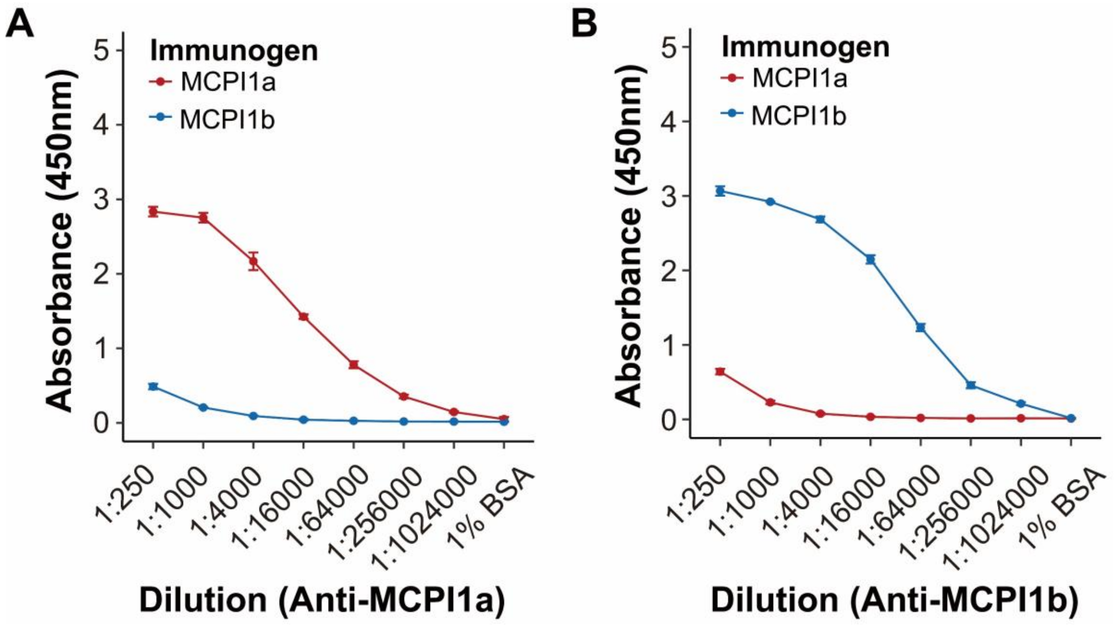

Fig. S28 Evaluating cross-reactivity of Anti-MCPI1a to MCPI1b and cross-reactivity of Anti-MCPI1b to MCPI1a using ELISA.

(A) After serial dilution, binding capacity of Anti-MCPI1a to MCPI1a or MCPI1b were measured using ELISA (mean ± SEM, n = 3). Absorbance at 450 nm was recorded and 1 % BSA solution was used as negative control.

(B) After serial dilution, binding capacity of Anti-MCPI1b to MCPI1a or MCPI1b were measured using ELISA (mean ± SEM, n = 3). Absorbance at 450 nm was recorded and 1 % BSA solution was used as negative control.

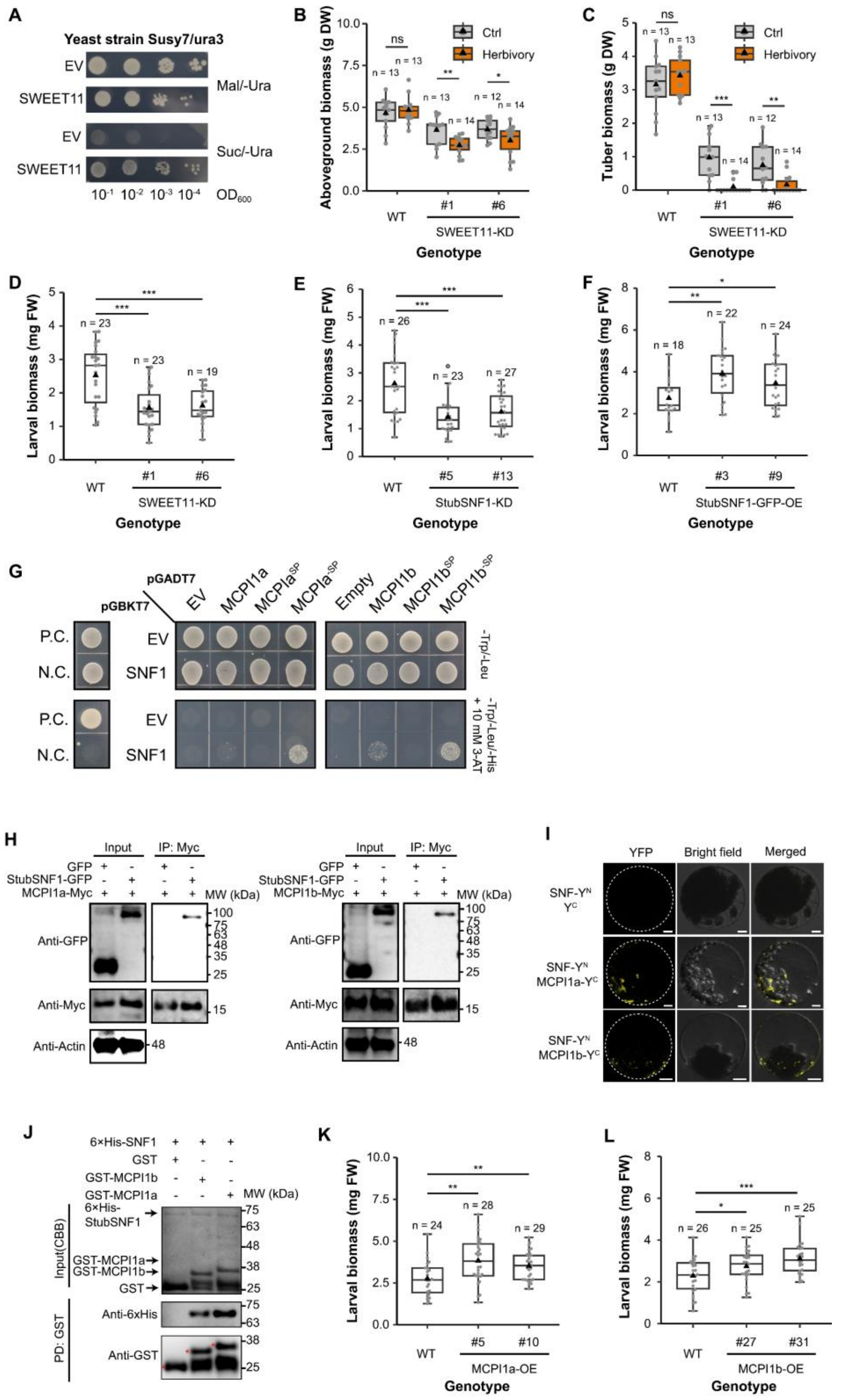

Fig. S29. Repeated experiments in the study.

(A) Figure 2K. (**B** – **C)** Figure 3F – G. (**D)** Figure 4E. (**E)** Figure 5G – H. (**G** – **L)** Figure 6A – E. Asterisks indicated significant differences between treatments or genotypes (Student's t test or Mann-Whitney U test, *p < 0.05, **p < 0.01, ***p < 0.001)

Legends for data S1 to S4

Data S1. The results of statistical analyses.

Data S2. The results of RNA-seq analysis in the study.

Data S3. Cloned genes (Sheet 1); StubSNF1 interactor candidates in Y2H screening (Sheet 2); primers (Sheet 3) and antibodies (Sheet 4) used in the study; optimized coding sequences for recombinant expression in *E. coli*. (Sheet 5).

Data S4. Source data of Figures and Supplementary Figures.

REFERENCES AND NOTES

1. E. I. Svensson, L. Råberg, Resistance and tolerance in animal enemy–victim coevolution. *Trends Ecol. Evol.* **25**, 267–274 (2010).

2. W. P. J. Smith, B. R. Wucher, C. D. Nadell, K. R. Foster, Bacterial defences: Mechanisms, evolution and antimicrobial resistance. *Nat. Rev. Microbiol.* **21**, 519–534 (2023).

3. J. Núñez-Farfán, J. Fornoni, P. L. Valverde, The evolution of resistance and tolerance to herbivores. *Annu. Rev. Ecol. Evol. Syst.* **38**, 541–566 (2007).

4. J. M. Gómez, R. Zamora, Thorns as induced mechanical defense in a long-lived shrub (*Hormathophylla Spinosa*, Cruciferae). *Ecology* **83**, 885–890 (2002).

5. C. Blanc, F. Coluccia, F. L'Haridon, M. Torres, M. Ortiz-Berrocal, E. Stahl, P. Reymond, L. Schreiber, C. Nawrath, J. Métraux, M. Serrano, The cuticle mutant *eca2* modifies plant defense responses to biotrophic and necrotrophic pathogens and herbivory insects. *Mol. Plant Microbe Interact.* **31**, 344–355 (2018).

6. R. V. Barbehenn, C. P. Constabel, Tannins in plant–herbivore interactions. *Phytochemistry* **72**, 1551–1565 (2011).

7. A. Steppuhn, K. Gase, B. Krock, R. Halitschke, I. T. Baldwin, Nicotine's defensive function in nature. *PLoS Biol.* **2**, e217 (2004).

8. R. Karban, I. T. Baldwin, *Induced Responses to Herbivory*. (University Of Chicago Press, 1997), 10.7208/chicago/9780226424972.001.0001.

9. S. Y. Strauss, A. A. Agrawal, The ecology and evolution of plant tolerance to herbivory. *Trends Ecol. Evol.* **14**, 179–185 (1999).

10. S. N. Johnson, M. W. Young, A. J. Karley, Protected raspberry production alters aphid–plant interactions but not aphid population size. *Agric. For. Entomol.* **14**, 217–224 (2011).

11. J. Schwachtje, P. E. H. Minchin, S. Jahnke, J. T. Dongen, U. Schittko, I. T. Baldwin, SNF1-related kinases allow plants to tolerate herbivory by allocating carbon to roots. *Proc. Natl. Acad. Sci. U.S.A.* **103**, 12935–12940 (2006).

12. F. P. Schiestl, H. Kirk, L. Bigler, S. Cozzolino, G. A. Desurmont, Herbivory and floral signaling: Phenotypic plasticity and tradeoffs between reproduction and indirect defense. *New Phytol.* **203**, 257–266 (2014).

13. O. Miler, D. Straile, How to cope with a superior enemy? Plant defence strategies in response to annual herbivore outbreaks. *J. Ecol.* **98**, 900–907 (2010).

14. P. Singh, A. Dave, F. E. Vaistij, D. Worrall, G. H. Holroyd, J. G. Wells, F. Kaminski, I. A. Graham, M. R. Roberts, Jasmonic acid-dependent regulation of seed dormancy following maternal herbivory in *Arabidopsis*. *New Phytol.* **214**, 1702–1711 (2017).

15. K. A. Stowe, R. J. Marquis, C. G. Hochwender, E. L. Simms, The evolutionary ecology of tolerance to consumer damage. *Annu. Rev. Ecol. Syst.* **20**, 565–595 (2000).

16. S. M. Yu, S. F. Lo, T. D. Ho, Source-sink communication: Regulated by hormone, nutrient, and stress cross-signaling. *Trends Plant Sci.* **20**, 844–857 (2015).

17. J. C. Schultz, H. M. Appel, A. P. Ferrieri, T. M. Arnold, Flexible resource allocation during plant defense responses. *Front. Plant Sci.* **4**, 324 (2013).

18. C. A. Robert, R. A. Ferrieri, S. Schirmer, B. A. Babst, M. J. Schueller, R. A. Machado, C. C. Arce, B. E. Hibbard, J. Gershenzon, T. C. Turlings, M. Erb, Induced carbon reallocation and compensatory growth as root herbivore tolerance mechanisms. *Plant Cell Environ.* **37**, 2613–2622 (2014).

19. R. A. Machado, A. P. Ferrieri, C. A. Robert, G. Glauser, M. Kallenbach, I. T. Baldwin, M. Erb, Leaf-herbivore attack reduces carbon reserves and regrowth from the roots via jasmonate and auxin signaling. *New Phytol.* **200**, 1234–1246 (2013).

20. K. N. Paige, T. G. Whitham, Overcompensation in response to mammalian herbivory: The advantage of being eaten. *Am. Nat.* **129**, 407–416 (1987).

21. Y. Chen, A. J. Miller, B. Qiu, Y. Huang, K. Zhang, G. Fan, X. Liu, The role of sugar transporters in the battle for carbon between plants and pathogens. *Plant Biotechnol. J.* **22**, 2844–2858 (2024).

22. K. Yamada, Y. Saijo, H. Nakagami, Y. Takano, Regulation of sugar transporter activity for antibacterial defense in *Arabidopsis*. *Science* **354**, 1427–1430 (2016).

23. K. Yamada, A. Mine, Sugar coordinates plant defense signaling. *Sci. Adv.* **10**, eadk4131 (2024).

24. S. Hulsmans, M. Rodriguez, B. De Coninck, F. Rolland, The SnRK1 energy sensor in plant biotic interactions. *Trends Plant Sci.* **21**, 648–661 (2016).

25. R. A. R. Machado, C. C. M. Arce, A. P. Ferrieri, I. T. Baldwin, M. Erb, Jasmonate-dependent depletion of soluble sugars compromises plant resistance to *Manduca sexta*. *New Phytol.* **207**, 91–105 (2015).

26. S. T. Behmer, Insect herbivore nutrient regulation. *Annu. Rev. Entomol.* **54**, 165–187 (2009).

27. H. A. Hespenheide, Bionomics of leaf-mining insects. *Annu. Rev. Entomol.* **36**, 535–560 (1991).

28. G. Csoka, *Leaf Mines and Leaf Miners*. (Hungarian Forest Research Institute, 2003).

29. R. J. Knecht, A. Swain, J. S. Benner, S. L. Emma, N. E. Pierce, C. C. Labandeira, Endophytic ancestors of modern leaf miners may have evolved in the Late Carboniferous. *New Phytol.* **240**, 2050–2057 (2023).

30. S. I. Rondon, The potato tuberworm: A literature review of its biology, ecology, and control. *Am. J. Potato Res.* **87**, 149–166 (2010).

31. S. I. Rondon, Y. Gao, "The journey of the potato tuberworm around the world" in *Moths - Pests of Potato*, *Maize and Sugar Beet*, K. Perveen, Ed. (IntechOpe; 2018), chap. 2.

32. A. Abdeen, A. Virgos, E. Olivella, J. Villanueva, X. Aviles, R. Gabarra, S. Prat, Multiple insect resistance in transgenic tomato plants over-expressing two families of plant proteinase inhibitors. *Plant Mol. Biol.* **57**, 189–202 (2005).

33. J. Quilis, D. Meynard, L. Vila, F. X. Aviles, E. Guiderdoni, B. San Segundo, A potato carboxypeptidase inhibitor gene provides pathogen resistance in transgenic rice. *Plant Biotechnol. J.* **5**, 537–553 (2007).

34. C. Zhang, Y. Li, J. Wang, X. Xue, G. Beuchat, L. Q. Chen, Two evolutionarily duplicated domains individually and post-transcriptionally control *SWEET* expression for phloem transport. *New Phytol.* **232**, 1793–1807 (2021).

35. J. Kim, E. Symeonidi, T. Y. Pang, T. Denyer, D. Weidauer, M. Bezrutczyk, M. Miras, N. Zöllner, T. Hartwig, M. M. Wudick, M. Lercher, L. Chen, M. C. P. Timmermans, W. B. Frommer, Distinct identities of leaf phloem cells revealed by single cell transcriptomics. *Plant Cell* **33**, 511–530 (2021).

36. J. A. Abelenda, S. Bergonzi, M. Oortwijn, S. Sonnewald, M. Du, R. G. F. Visser, U. Sonnewald, C. W. B. Bachem, Source-sink regulation is mediated by interaction of an FT homolog with a SWEET protein in potato. *Curr. Biol.* **29**, 1178–1186.e6 (2019).

37. J. Blanford, Z. Zhai, M. D. Baer, G. Guo, H. Liu, Q. Liu, Q. Raugei, Q. Shanklin, Molecular mechanism of trehalose 6-phosphate inhibition of the plant metabolic sensor kinase SnRK1. *Sci. Adv.* **10**, eadn0895 (2024).

38. B. Martineau, K. E. McBride, C. M. Houck, Regulation of metallocarboxypeptidase inhibitor gene expression in tomato. *Mol. Gen. Genet.* **228**, 281–286 (1991).

39. C. M. Pieterse, D. Van der Does, C. Zamioudis, A. Leon-Reyes, S. C. Van Wees, Hormonal modulation of plant immunity. *Annu. Rev. Cell Dev. Biol.* **28**, 489–521 (2012).

40. M. Erb, P. Reymond, Molecular interactions between plants and insect herbivores. *Annu. Rev. Plant Biol.* **70**, 527–557 (2019).

41. J. Zhong, J. Zhang, Y. Zhang, Y. Ge, W. He, C. Liang, Y. Gao, Z. Zhu, R. A. R. Machado, W. Zhou, Heat stress reprograms herbivory-induced defense responses in potato plants. *BMC Plant Biol.* **24**, 677 (2024).

42. N. E. Turley, R. M. Godfrey, M. T. J. Johnson, Evolution of mixed strategies of plant defense against herbivores. *New Phytol.* **197**, 359–361 (2013).

43. K. Poveda, M. I. G. Jiménez, A. Kessler, The enemy as ally: Herbivore-induced increase in crop yield. *Ecol. Appl.* **20**, 1787–1793 (2010).

44. P. Kumar, E. Garrido, K. Zhao, Y. Zheng, S. Alseekh, E. Vargas-Ortiz, A. R. Fernie, Z. Fei, K. Poveda, G. Jander, *Tecia solanivora* infestation increases tuber starch accumulation in Pastusa Suprema potatoes. *J. Integr. Plant Biol.* **60**, 1083–1096 (2018).

45. G. Mailloux, N. J. Bostanian, M. R. Binns, Density-yield relationships for colorado potato beetle adults on potatoes. *Phytoparasitica* **23**, 101–118 (1995).

46. G. Mailloux, M. R. Binns, N. J. Bostanian, Density yield relationships and economic injury level model for the colorado potato beetle larvae on potatoes. *Res. Popul. Ecol.* **33**, 101–113 (1991).

47. B. A. Nault, G. G. Kennedy, Limitations of using regression and mean separation analyses for describing the response of crop yield to defoliation: A case study of the colorado potato beetle (Coleoptera: Chrysomelidae) on potato. *J. Econ. Entomol.* **91**, 7–20 (1998).

48. I. Kaplan, G. P. Dively, R. F. Denno, Variation in tolerance and resistance to the leafhopper *Empoasca fabae* (Hemiptera: Cicadellidae) among potato cultivars: Implications for action thresholds. *J. Econ. Entomol.* **101**, 959–968 (2008).

49. F. Kappel, J. T. A. Proctor, Simulated spotted tentiform leafminer injury and its influence on growth and fruiting of apple trees. *J. Amer. Soc. Hort. Sci.* **111**, 64–69 (1986).

50. T. G. Shanower, A. P. Gutierrez, J. A. Wightman, Impact of the groundnut leafminer, *Aproaerema modicella* (deventer) (Lepidoptera: Gelechiidae) on growth and yield of two groundnut cultivars. *Insect Sci. Appl.* **16**, 87–91 (1995).

51. S. Salleo, A. Nardini, F. Raimondo, M. A. Lo Gullo, F. Pace, P. Giacomich, Effects of defoliation caused by the leaf miner *Cameraria ohridella* on wood production and efficiency in *Aesculus hippocastanum* growing in north-eastern italy. *Trees* **17**, 367–375 (2003).

52. R. E. Foster, C. A. Sanchez, Effect of *Liriomyza trifolii* (Diptera: Agromyzidae) larval damage on growth, yield, and cosmetic quality of celery in Florida. *J. Econ. Entomol.* **81**, 1721–1725 (1988).

53. G. C. Percival, I. Barrow, K. Noviss, I. Keary, P. Pennington, The impact of horse chestnut leaf miner (*Cameraria ohridella* Deschka and Dimic; HCLM) on vitality, growth and reproduction of *Aesculus hippocastanum* L. *Urban For. Urban Green.* **10**, 11–17 (2011).

54. M. X. Jiang, J. A. Cheng, Interactions between the striped stem borer *Chilo suppressalis* (Walk.) (Lep., Pyralidae) larvae and rice plants in response to nitrogen fertilization. *J. Pest Sci.* **76**, 124–128 (2003).

55. M. Nicolas, R. Torres-Pérez, V. Wahl, E. Cruz-Oró, M. L. Rodríguez-Buey, A. M. Zamarreño, B. Martín-Jouve, J. M. García-Mina, J. C. Oliveros, S. Prat, P. Cubas, Spatial control of potato tuberization by the TCP transcription factor BRANCHED1b. *Nat Plants* **8**, 281–294 (2022).

56. L. Q. Chen, B. H. Hou, S. Lalonde, H. Takanaga, M. L. Hartung, X. Q. Qu, W. J. Guo, J. G. Kim, W. Underwood, B. Chaudhuri, D. Chermak, G. Antony, F. F. White, S. C. Somerville, M. B. Mudgett, W. B. Frommer, Sugar transporters for intercellular exchange and nutrition of pathogens. *Nature* **468**, 527–532 (2010).

57. M. Andargie, J. Li, Expression of the *Arabidopsis* SWEET genes during rice false smut infection in the transgenic *Arabidopsis thaliana* containing increased levels of ATP and sucrose. *J. Plant Biochem. Biotechnol.* **28**, 509–520 (2019).

58. R. Breia, A. Conde, D. Pimentel, C. Conde, A. M. Fortes, A. Granell, H. Gerós, VvSWEET7 Is a mono- and disaccharide transporter up-regulated in response to *Botrytis cinerea* infection in grape berries. *Front. Plant Sci.* **10**, 1753 (2020).

59. J. Chong, M. Piron, S. Meyer, D. Merdinoglu, C. Bertsch, P. Mestre, The SWEET family of sugar transporters in grapevine: VvSWEET4 is involved in the interaction with *Botrytis cinerea*. *J. Exp. Bot.* **65**, 6589–6601 (2014).

60. L. Yu, Y. Chen, X. Zeng, Y. Lou, I. T. Baldwin, R. Li, Brown planthoppers manipulate rice sugar transporters to benefit their own feeding. *Curr. Biol.* **34**, 2990–2996.e4 (2024).

61. Y. Wu, S. K. Lee, Y. Yoo, J. Wei, S. Y. Kwon, S. W. Lee, J. S. Jeon, G. An, Rice transcription factor OsDOF11 modulates sugar transport by promoting expression of *Sucrose Transporter* and *SWEET* genes. *Mol. Plant* **11**, 833–845 (2018).

62. T. Qiu, S. Wei, K. Fang, M. Zhang, Y. Li, Y. Feng, Y. Cheng, S. Zhang, J. Tian, A. Gao, Q. Yang, M. Yang, V. Bhadauria, J. Li, Y. Peng, W. Zhao, The atypical Dof transcriptional factor OsDes1 contributes to stay-green, grain yield, and disease resistance in rice. *Sci. Adv.* **10**, eadp0345 (2024).

63. P. Rueda-Romero, C. Barrero-Sicilia, A. Gomez-Cadenas, P. Carbonero, L. Onate-Sanchez, *Arabidopsis thaliana* DOF6 negatively affects germination in non-after-ripened seeds and interacts with TCP14. *J. Exp. Bot.* **63**, 1937–1949 (2012).

64. M. Manna, T. Thakur, O. Chirom, R. Mandlik, R. Deshmukh, P. Salvi, Transcription factors as key molecular target to strengthen the drought stress tolerance in plants. *Physiol. Plant.* **172**, 847–868 (2021).

65. X. Zou, H. Sun, DOF transcription factors: Specific regulators of plant biological processes. *Front. Plant Sci.* **14**, 1044918 (2023).

66. Z. Y. Mao, Y. Ge, Y. D. Zhang, J. Zhong, A. Munawar, Z. R. Zhu, W. W. Zhou, Disentangling the potato tuber moth-induced early-defense response by simulated herbivory in potato plants. *Front. Plant Sci.* **13**, 902342 (2022).

67. Q. Chen, T. Hu, X. Li, C. Song, J. Zhu, L. Chen, Y. Zhao, Phosphorylation of SWEET sucrose transporters regulates plant root:shoot ratio under drought. *Nat Plants* **8**, 68–77 (2022).

68. M. Durrant, J. Boyer, W. W. Zhou, I. T. Baldwin, S. Q. Xu, Evidence of an evolutionary hourglass pattern in herbivory-induced transcriptomic responses. *New Phytol.* **215**, 1264–1273 (2017).

69. W. Jing, S. Uddin, R. Chakraborty, D. T. Van Anh, D. M. Macoy, S. O. Park, G. R. Ryu, Y. H. Kim, J. Cha, W. Kim, M. G. Kim, Molecular characterization of HEXOKINASE1 in plant innate immunity. *Appl. Biol. Chem.* **63**, 63–76 (2020).

70. G. Geniana da Silva, E. Paula Corrêa, B. Maria Cristina, Carboxypeptidase inhibitors from *Solanaceae* as a new subclass of pathogenesis related peptide aiming biotechnological targets for plant defense. *Front. Mol. Biosci.* **10**, 1259026 (2023).

71. R. Lebecka, M. Kistowski, J. Dębski, K. Szajko, Z. Murawska, W. Marczewski, Quantitative proteomic analysis of differentially expressed proteins in tubers of potato plants differing in resistance to *Dickeya solani*. *Plant and Soil* **441**, 317–329 (2019).

72. J. Quilis, B. López-García, D. Meynard, E. Guiderdoni, B. San Segundo, Inducible expression of a fusion gene encoding two proteinase inhibitors leads to insect and pathogen resistance in transgenic rice. *Plant Biotechnol. J.* **12**, 367–377 (2013).

73. B. Molesini, V. Dusi, F. Pennisi, G. P. Di Sansebastiano, S. Zanzoni, A. Manara, A. Furini, F. Martini, G. L. Rotino, T. Pandolfini, TCMP-2 affects tomato flowering and interacts with BBX16, a homolog of the *Arabidopsis* B-box MiP1b. *Plant Direct* **4**, e00283 (2020).

74. T. Zust, A. A. Agrawal, Trade-Offs between plant growth and defense against insect herbivory: An emerging mechanistic synthesis. *Annu. Rev. Plant Biol.* **68**, 513–534 (2017).

75. R. K. Monson, A. M. Trowbridge, R. L. Lindroth, M. T. Lerdau, Coordinated resource allocation to plant growth-defense trade-offs. *New Phytol.* **233**, 1051–1066 (2021).

76. H. Y. Chen, J. H. Huh, Y. C. Yu, L. H. Ho, L. Q. Chen, D. Tholl, W. B. Frommer, W. J. Guo, The *Arabidopsis* vacuolar sugar transporter SWEET2 limits carbon sequestration from roots and restricts *Pythium* infection. *Plant J.* **83**, 1046–1058 (2015).

77. Y. Li, Y. Wang, H. Zhang, Q. Zhang, H. Zhai, Q. Liu, S. He, The plasma membrane-localized sucrose transporter lbSWEET10 contributes to the resistance of sweet potato to *Fusarium oxysporum*. *Front. Plant Sci.* **8**, 197 (2017).

78. X. Wang, Z. Wang, X. Tang, J. Qin, X. Zhou, L. Gu, H. Bian, L. Sun, H. Huang, R. Yang, J. Wang, S. Wang, S. Chen, Z. Yang, W. Zhao, The SlDOF9-SlSWEET17 module: A switch for controlling sugar distribution between nematode induced galls and roots in tomato. *Adv. Sci.* **12**, e2501771 (2025).

79. E. Meteier, S. La Camera, M. Goddard, H. Laloue, P. Mestre, J. Chong, Overexpression of the VvSWEET4 transporter in grapevine hairy roots increases sugar transport and contents and enhances resistance to *Pythium irregulare*, a soilborne pathogen. *Front. Plant Sci.* **10**, 884 (2019).

80. Y. Xiao, Q. Wang, M. Erb, T. C. J. Turlings, L. Ge, L. Hu, J. Li, X. Han, T. Zhang, J. Lu, G. Zhang, Y. Lou, Specific herbivore-induced volatiles defend plants and determine insect community composition in the field. *Ecol. Lett.* **15**, 1130–1139 (2012).

81. X. He, Y. Wang, A. Munawar, J. Zhu, J. Zhong, Y. Zhang, H. Guo, Z. Zhu, I. T. Baldwin, W. Zhou, Manipulating stomatal aperture by silencing *StSLAC1* affects potato plant–herbivore–parasitoid tritrophic interactions under drought stress. *New Phytol.* **245**, 2133–2149 (2025).

82. Y. Qi, J. Wu, Z. Yang, H. Li, L. Liu, H. Wang, X. Sun, X. Wu, J. Nie, J. Zhou, M. Xu, X. Wu, S. Breen, R. Yu, D. Cheng, Q. Sun, H. Qiu, Y. Zuo, P. C. Boevink, P. R. J. Birch, Z. Tian, Chloroplast elongation factors break the growth–immunity trade-off by simultaneously promoting yield and defence. *Nat Plants* **10**, 1576–1591 (2024).

83. B. B. Salam, F. Barbier, R. Danieli, P. Teper-Bamnolker, C. Ziv, L. Spíchal, K. Aruchamy, Y. Shnaider, D. Leibman, F. Shaya, M. Carmeli-Weissberg, A. Gal-On, J. Jiang, N. Ori, C. Beveridge, D. Eshel, Sucrose promotes stem branching through cytokinin. *Plant Physiol.* **185**, 1708–1721 (2021).

84. R. A. R. Machado, I. T. Baldwin, M. Erb, Herbivory-induced jasmonates constrain plant sugar accumulation and growth by antagonizing gibberellin signaling and not by promoting secondary metabolite production. *New Phytol.* **215**, 803–812 (2017).

85. T. Arnold, H. Appel, V. Patel, E. Stocum, A. Kavalier, J. Schultz, Carbohydrate translocation determines the phenolic content of *Populus* foliage: A test of the sink-source model of plant defense. *New Phytol.* **164**, 157–164 (2004).

86. M. Javelle, C. F. Marco, M. Timmermans, Hybridization for the precise localization of transcripts in plants. *J. Vis. Exp.* **57**, e3328 (2011).

87. M. I. Love, W. Huber, S. Anders, Moderated estimation of fold change and dispersion for RNA-seq data with DESeq2. *Genome Biol.* **15**, 550 (2014).

88. J. E. Lunn, R. Feil, J. H. Hendriks, Y. Gibon, R. Morcuende, D. Osuna, W. R. Scheible, P. Carillo, M. R. Hajirezaei, M. Stitt, Sugar-induced increases in trehalose 6-phosphate are correlated with redox activation of ADP glucose pyrophosphorylase and higher rates of starch synthesis in *Arabidopsis thaliana*. *Biochem. J.* **397**, 139–148 (2006).

89. M. Li, H. Xie, M. He, W. Su, Y. Yang, J. Wang, G. Ye, Y. Zhou, Genome-wide identification and expression analysis of the StSWEET family genes in potato (*Solanum tuberosum* L.). *Genes Genom.* **42**, 135–153 (2020).

90. A. T. Mata, T. F. Jorge, J. Ferreira, M. do Rosário Bronze, D. Branco, P. Fevereiro, S. Araújo, C. António, Analysis of low abundant trehalose-6-phosphate and related metabolites in *Medicago truncatula* by hydrophilic interaction liquid chromatography–triple quadrupole mass spectrometry. *J. Chromatogr. A* **1477**, 30–38 (2016).

91. F. Wang, D. Zhu, X. Huang, S. Li, Y. Gong, Q. Yao, X. Fu, L.-M. Fan, X. W. Deng, Biochemical insights on degradation of *Arabidopsis* DELLA proteins gained from a cell-free assay system. *Plant Cell* **21**, 2378–2390 (2009).
